# Sodium-dependent multivitamin transporter deficiency caused by novel *SLC5A6* mutations: a clinical case and personalized mice model study

**DOI:** 10.3389/fcell.2026.1925753

**Published:** 2026-10-09

**Authors:** Anastasia V. Priymak, Olga A. Averina, Margarita A. Novikova, Oleg A. Permyakov, Svetlana A. Garmash, Alexander Yu Rudenko, Vlasta O. Bobrynina, Alexander M. Mazur, Artem V. Artiukhov, Vasily A. Aleshin, Olga A. Bazhanova, Polina A. Zotova, Mikhail O. Bortyazh, Mariia A. Emelianova, Olga O. Grigorieva, Ekaterina A. Nikolaeva, Vasily N. Manskikh, Olga A. Dontsova, Ekaterina Yu Zakharova, Egor B. Prokhortchouk, Petr V. Sergiev

**Affiliations:** 1 Belozersky Institute of Physico-Chemical Biology, Lomonosov Moscow State University, Moscow, Russia; 2 Faculty of Chemistry, Lomonosov Moscow State University, Moscow, Russia; 3 Faculty of Biology, Lomonosov Moscow State University, Moscow, Russia; 4 Genoanalytica Laboratory, Moscow, Russia; 5 Department of Biological Chemistry, Sechenov University, Moscow, Russia; 6 Research Center for Medical Genetics, Moscow, Russia; 7 Center for Bio- and Medical Technologies, Moscow, Russia; 8 Research and Clinical Institute of Pediatrics Named After Yuri Veltischev, Pirogov Russian National Research Medical University of the Russian Ministry of Health, Moscow, Russia; 9 Shemyakin–Ovchinnikov Institute of Bioorganic Chemistry, Moscow, Russia; 10 Federal Research Center “Fundamentals of Biotechnology”, Russian Academy of Sciences, Moscow, Russia; 11 Group of Epigenetic Editing, Research Center for Genetics and Life Sciences, Sirius University of Science and Technology, Moscow, Russia

**Keywords:** biotin, hereditary disease, hSMVT, lipoate, mice model, mutation, pantothenate

## Abstract

The *SLC5A6* gene encodes the sodium-dependent multivitamin transporter (SMVT), which transports biotin, pantothenate, and lipoic acid. Mutations of the *SLC5A6* gene result in a hereditary syndrome whose symptoms range from early-onset, life-threatening systemic disorder to relatively mild, late-onset motor neuropathy. We describe a clinical case of a patient with previously unknown compound heterozygous missense mutations R280W and P437Q in the *SLC5A6* gene. The patient exhibited a severe metabolic disorder accompanied by an elevated blood concentration of 3-hydroxyisovalerylcarnitine (C5OH), likely explained by biotin deficiency. However, treatment with biotin was unsuccessful. To determine the pathogenicity of these new missense *SLC5A6* mutations, we created a personalized mouse model matching the patient’s genotype. The P437Q mutation appears to be hypofunctional, whereas R280W is lethal when homozygous. The mouse model recapitulated the physiological and biochemical manifestations of *SLC5A6* mutations in humans, including increased mortality, reduced weight and strength, and elevated blood C5OH concentration. It allowed us to model the consequences of biotin treatment, which likely exacerbated the pathology by competing with pantothenate for a barely functional transporter, thereby increasing blood fatty acyl carnitine concentrations, indicative of fatty acid oxidation deficiency. We recommend analyzing the *SLC5A6* gene sequence in all cases of elevated C5OH that are not explained by mutations in other known genes in the biotin pathway before prescribing treatment.

## 1 Introduction

SMVT protein plays a crucial role in the uptake of essential water-soluble vitamins, namely, biotin, pantothenate, and lipoate, from dietary sources. It is primarily expressed in the intestinal epithelium, placenta (Grassl, 1992), and brain blood vessels (Park and Sinko, 2005), and is responsible for the intestinal absorption of these vitamins (Said et al., 1987; Balamurugan et al., 2003), as well as for the transfer of these solutes across the blood-brain barrier (Uchida et al., 2015), to the fetus (Ben-Eliezer et al., 2020), and to other tissues. While SMVT is considered to serve as the primary transporter for biotin uptake (Balamurugan et al., 2003), additional biotin transporters were described for keratinocytes (Grafe et al., 2003) and peripheral blood mononuclear cells (Zempleni and Mock, 1998). Also, the biotin salvage pathway via biotinidase (BTD) contributes to the pool of this vitamin, utilizing available biotinylated proteins (Cole et al., 1994). Pantothenate, which is utilized for coenzyme A (CoA) biosynthesis (Barritt et al., 2024), is transported predominantly via SMVT (Uchida et al., 2015); only at supraphysiological concentrations, some unfacilitated transmembrane diffusion of pantothenate may be detected (Shibata et al., 1983; Smith and Milner, 1985). While free lipoate is transported by SMVT, in mammals, the functional lipoyl cofactor is synthesized directly on protein-bound intermediates through the LIPT2–LIAS–LIPT1 pathway (Morikawa et al., 2001; Cao et al., 2018), which could not be substituted by free lipoate uptake (Yi and Maeda, 2005).

SMVT protein consists of 635 amino acids (Wang et al., 1999) and features 13 transmembrane domains (Zhen et al., 2026). It is encoded by the *SLC5A6* gene, located on chromosome 2p23.3. SMVT belongs to the sodium-ion-dependent transporter family SLC5, whose members co-transport cargo with Na^+^ ion(s), thereby using the electrochemical gradient established by the Na^+^/K^+^ ATPase to drive nutrient uptake against their concentration gradients (Wright, 2013).

As the SMVT protein plays a crucial role in the uptake of water-soluble vitamins, mutations in the *SLC5A6* gene can lead to a range of clinical disorders characterized by deficiencies of these vitamins, resulting in various systemic and neurological symptoms (Subramanian et al., 2017; Byrne et al., 2019). Pathogenic mutations in *SLC5A6* are typically compound heterozygous and may include missense, nonsense, and frameshift mutations. These mutations disrupt the function of the SMVT, leading to metabolic disturbances (Subramanian et al., 2017; Montomoli et al., 2023) ranging from severe, early-onset, potentially lethal disease, sodium multivitamin transporter deficiency (SMVTD; MIM 618973), to less dangerous childhood-onset biotin-responsive peripheral motor neuropathy (COMNB; MIM 619903).

Treatment for *SLC5A6* mutations primarily involves high-dose supplementation with biotin, pantothenic acid, and lipoic acid, which has been shown to significantly improve clinical outcomes in most cases (Byrne et al., 2019). Early diagnosis and intervention are essential for optimizing treatment efficacy and improving the quality of life for affected individuals (Schwantje et al., 2019).

In this study, we retrospectively report a case of a deceased patient carrying compound heterozygous c.1294 C>T (NM_021095.4, p.R280W) and c.1310 C>A (NM_021095.4, p.P437Q) mutations (further referred to as R280W and P437Q) in the *SLC5A6* gene. Neither any of these mutations nor the patient has been reported previously, to our knowledge. To elucidate the pathogenic mechanism underlying *SLC5A6* mutations, we created and validated a personalized mouse model carrying the compound heterozygous *SLC5A6* ^P437Q/R280W^ genotype matching the patient’s.

## 2 Materials and methods

### 2.1 Patient acylcarnitine profile and organic acids analysis

Quantification of amino acids and acylcarnitines in whole blood is conducted using MS/MS (QSight 225MD UHPLC Screening System (United States); NeoBase NBS kit (PerkinElmer, Finland). Organic acids are measured using GC-MS (GCMS-TQ8050 EI, SHIMADZU). The preparation of urine samples involves extraction with organic solvents at pH 1–2, followed by derivatization with methoxyamine hydrochloride and N, O-bis (trimethylsilyl)trifluoroacetamide.

### 2.2 Mice

Experiments with mice were conducted in strict compliance with national and international guidelines for the Care and Use of Laboratory Animals. The animal study was carried out in accordance with the ARRIVE guidelines. Group sizes for each experiment are indicated in the corresponding Figure legends.

Animals were kept in individually ventilated cages (IVC system, TECNIPLAST S. p.A., Italy) with free access to food and water purified by reverse osmosis, in an environment free of specific pathogens, with a light regime of 12/12 (light on at 09:00), in rooms with an air exchange rate of more than 15 r/h, at 20 °C–24 °C, humidity 30%–70%. Wood chips with minimal dust formation were used as bedding. Shelters and nest-building materials made of natural materials were used to enrich the environment. All materials supplied to the animals were sterilized by autoclaving.

All invasive procedures were carried out under anesthesia—isoflurane inhalation (Bodnar et al., 2023) or general anesthesia using a Zoletil-Xylazine combination (Gurova et al., 2022).

The animals were euthanized under general anesthesia with subsequent cervical dislocation according to NIH guidelines (Carbone et al., 2012).

### 2.3 Mouse genome editing

Desired mutations were introduced by the CRISPR/Cas9 system. SgRNA was designed using the CHOPCHOP web resource (Labun K et al., 2019). SgRNAs were obtained by *in vitro* transcription with T7 phage RNA polymerase (Thermo Fisher) from PCR-amplified templates generated by PCR amplification of the plasmid pX458 (Ran et al., 2013). Spacer sequences along with the T7 promoter were introduced via PCR primers (see Supplementary Table S10 for primer sequences). The obtained sgRNA was mixed with Cas9 mRNA (Thermofisher) and diluted in the filtered microinjection buffer (10 mM Tris, 0.1 mM EDTA, pH 8) to final concentrations of 25 ng/µL sgRNA, 50 ng/μL Cas9 mRNA, and 10 ng/μL ssDNA template for homologous recombination (see Supplementary Table S10 for primer sequences).

C57BL/6J and CBA mice (Federal Research Center Institute of Cytology and Genetics, Siberian Branch, Russian Academy of Sciences (ICG SB RAS), Novosibirsk, Russia), with SPF status, were mated to obtain F1 hybrids (C57BL/6J × CBA). Zygotes were obtained by mating the hybrid superovulated female mice with males using a standard procedure (Averina et al., 2020). Mouse zygotes were microinjected using Eppendorf TransferMan NK4-R micromanipulators, FemtoJet 4i injector mounted on a Nikon Ti-E microscope equipped with differential interference contrast optics into the pronucleus and transferred into the oviducts of pseudo-pregnant female F1 hybrids C57BL/6J × CBA mice. In total, 3,552 zygotes were microinjected with the construct for the R280W mutation; 778 survived (22%) and were transferred to the oviducts of 45 recipients, giving birth to 23 pups, 1 of which carried the desired mutation (4%). To produce the P437Q mutation, 1,466 zygotes were microinjected; 575 survived (39%) and were transferred to the oviducts of 32 recipients, giving birth to 18 pups, 4 of which carried the desired mutation (22%). To avoid potential confounding effects of unlikely secondary mutations, the founder pups were backcrossed to the C57BL/6J strain for 3 generations. Mice C57BL/6J *Slc5a6* ^P437Q^, C57BL/6J *Slc5a6* ^P437Q/R280W^ were obtained by breeding of C57BL/6J *Slc5a6* ^WT/P437Q^, C57BL/6J *Slc5a6* ^P437Q^ and C57BL/6J *Slc5a6* ^WT/R280W^ mice. To offset for remaining unlikely secondary mutations, wild-type littermates of mice from the last backcross were used to establish a control mouse cohort.

### 2.4 Genotyping

Genotyping of the mice was performed from tiny pieces of tail. DNA was extracted with QuickExtract™ DNA Extraction Solution (Lucigen) according to the manufacturer’s instructions and analyzed by PCR with Taq DNA polymerase (Evrogen) (see Supplementary Table S10 for primer sequences) and by Sanger sequencing of the amplicon.

### 2.5 Immunoblot analysis

Mice liver tissues were homogenized in 5 volumes of ice-cold RIPA buffer (50 mM Tris/HCl pH 7.5, 150 mM NaCl, 1% (w/v) IGEPAL CA-630, 1% (w/v) sodium deoxycholate, 0.1% (w/v) SDS, 2 mM EDTA) supplemented with HaltTM Protease Inhibitor Cocktail (Thermo Fisher Scientific, United States) with 1.6 mm stainless steel beads using Precellys Evolution Touch Homogenizer (Bertin Technologies, France) at 5800 rpm for three 20 s cycles, separated by 20 s cooling intervals on ice. Homogenates were incubated on ice for 20–30 min, transferred to 1.5 mL tubes, and centrifuged at 16,100 g for 10 min at +4 °C. Supernatants were transferred to a new tube, and centrifugation was repeated two more times at 16,100 g for 10 min at +4 °C. Final supernatants, diluted 5-fold in RIPA buffer, were used for protein quantification and subsequent Western blot analysis. Total protein concentration was measured by DCTM Protein Assay (Bio-Rad, United States). Lysates containing 10 μg of total protein were adjusted to equal volumes with RIPA buffer, mixed with 4x Laemmli buffer (250 mM Tris/HCl pH 6.8, 8% (w/v) SDS, 40% (v/v) glycerol, 0.2%–0.4% BPB, 20% (v/v) β-mercaptoethanol), and heated at 95 °C for 4–5 min. Samples were loaded and resolved on a 1.5 mm, 8% (29:1 (w/w) acrylamide: bisacrylamide) Tris-Gly SDS-polyacrylamide gel containing 0.5% (v/v) of 2,2,2-trichloroethanol (TCE) (Macklin, China) in the resolving gel for subsequent total protein visualization (Ladner et al., 2004). Prestained Protein Ladder Rav-10 (Biolabmix, Russia) was used to estimate molecular weights. After disassembly, gel was illuminated under UV for 2.5 min using Stain-Free Gel imaging program ChemiDoc MP Universal Hood III (Bio-Rad, United States), and proteins were transferred onto Pierce PVDF Transfer Membrane (Thermo Fisher Scientific, United States) or Invitrogen™ Low Fluorescence PVDF Membrane (Thermo Fisher Scientific, United States) in Dunn carbonate buffer (10 mM NaHCO_3_, 3 mM Na_2_CO_3_, 15% (v/v) EtOH, with or without 1 mM SDS) (Dunn, 1986) at 40–50 V for 1.5 h using Mini Trans-Blot® Cell (Bio-Rad, United States). After transfer, the total protein was visualized on the membrane using the Stain-Free Blot imaging program on the ChemiDoc MP Universal Hood III (Bio-Rad, United States). Membrane was blocked in 3% BSA (MP Biomedicals, United States) in TBST (50 mM Tris/HCl pH 7.6, 150 mM NaCl, 0.05% (v/v) Tween 20) for 1.5 h at room temperature and incubated overnight at +4 °C with primary anti-lipoic acid antibody (1:5,000, Sigma-Aldrich Cat# 437695-100UL, United States) or Str-HRP conjugate (1:10,000, Imtek Cat# P-S Avs, Russia), diluted in 1% BSA in TBST. After 4–5 washes in TBST for 5–10 min, the membrane was incubated with HRP-conjugated secondary antibodies (1:10,000, Thermo Fisher Scientific, Cat# 31460, United States) for 1 h at room temperature; this step was omitted for biotinylated protein detection with Str-HRP conjugate. After 4–5 washes in TBST for 5–10 min, chemiluminescent signals were developed using Clarity Western ECL Substrate (Bio-Rad, United States) and visualized with ChemiDoc MP Universal Hood III (Bio-Rad, United States). For quantification, specific band signals were normalized to TCE-based total protein visualization using Image Lab Software (version 6.1) (Bio-Rad, United States). Four biological replicates were used for each mouse type. Significance was calculated with a Kruskal-Wallis test with Dunn’s multiple comparisons.

### 2.6 Analysis of homozygous mouse strains’ embryonic lethality

At all stages of the study, heterozygous mice carrying either *Slc5a6* ^P437Q^ or *Slc5a6* ^wt/R280W^ mutations were crossed with each other. The evaluation is based on the distribution of genotypes across the litter.

### 2.7 Mouse weight monitoring

The weight assessment was conducted once a week for each mouse. Mice were placed on the PA-2102 scale (OHAUS, United States), and their weight was recorded.

### 2.8 Grip strength

The test was designed to evaluate maximum voluntary strength (grip force) and is commonly used to assess limb strength and neurological deficits in mice and rats (Rogers et al., 2001). The experiment was performed after 6 h of food deprivation to avoid differences due to each animal’s feeding time. The mouse was gently pulled back by its tail, ensuring it gripped the top portion of the grid, with the torso remaining horizontal, and the maximum grip strength displayed on the screen was recorded. This procedure was repeated with a 10 s range until 10 results were obtained. After selecting the five highest values, the mean was calculated.

### 2.9 Open field test

To conduct the test, each mouse was placed in the center of a 24 × 24 × 24 cm arena for 3 min under illumination at 580–600 lux. Behavior was recorded from above using a MindVision MV-GE134GM camera at 60.50 frames/s and analyzed using EthoVision XT version 14.0.1326 (Noldus Information Technology, Wageningen, the Netherlands). The animal’s center point was used to track it. The central region of interest (ROI) was defined as a 10 × 10 cm square located in the center of the arena. Mean movement speed, total distance traveled, frequency of entries into the central ROI, total time spent in the central ROI, number of grooming acts, and total grooming duration were recorded (Bailey and Crawley, 2009). No additional user-defined tracking filters were applied.

### 2.10 Box maze test

To assess associative memory, we used a box-maze task based on the one-day learning procedure described by Darvas et al. (2020) and modified in our laboratory. The apparatus consisted of a circular arena (30 cm internal diameter) with eight visually identical holes (5 cm diameter). During testing, one hole was connected to a dark shelter via a 20-cm tube, while the remaining seven served as false holes. At the beginning of each trial, the mouse was placed in a central start cylinder for 30 s, then allowed to explore the arena for up to 120 s. Animals underwent four training trials separated by 1-h intervals. If the target hole was not found within 120 s, the mouse was gently guided to it. A test trial using the same procedure was conducted 48 h after training, and latency to enter the target tube was recorded.

### 2.11 Analysis of whole blood amino acid and acylcarnitine profile

Blood was collected via submandibular vein puncture in anesthetized mice, with samples fixed on polystyrene plates (Golde et al., 2005). Blood analysis was performed using tandem mass spectrometry (MS/MS) by flow injection (Sciex API3200, Framingham, MA, United States). C0 concentrations and individual acylcarnitine concentrations were quantified by multiple reaction monitoring. Data evaluation was performed with Analyst® 1.5.2 Instrument Control and Data Processing Software (Sciex). A plate template was prepared to identify dried blood spot (DBS) samples, blanks, and controls. Using a manual or automated puncher, 3 mm DBS, blanks, and controls were punched into the wells of a polystyrene plate according to the predefined template. Daily Solution was prepared from internal standards as follows: 50 µL each of AA IS and AC IS (stored at −20 °C throughout the expiration period indicated on the package) were added to 9.9 mL of Extraction Solution, then gently mixed. Using a multichannel pipette, 90 µL of the Daily Solution was dispensed into each well of the plate containing DBS samples. The plate was sealed with an adhesive film and incubated for 30 min at 30 °C with orbital shaking (650–750 rpm). After incubation, the adhesive film was removed, and 75 µL of each sample was transferred to a new polypropylene plate. Samples were dried under a stream of nitrogen or air, then 50 µL of 3 N HCl in butanol was added. The plate was resealed using heat-stable film with a plate sealer and incubated for 30 min at 60 °C. After incubation, the film was removed, and samples were dried again under a nitrogen or an air stream. Next, 75 µL of Reconstruction Solution was added to each well, and the plate was sealed with aluminum foil. The plate was incubated for an additional 10 min at 27 °C (650–750 rpm). Following incubation, the plate was loaded into the autosampler. Prior to analysis, the mobile phase (Flow Solvent) was verified to ensure sufficient volume for the run, and the analysis was then initiated using Analyst software.

### 2.12 Assay of coenzyme A in the mouse liver

Methanol extraction of metabolites from the liver was performed as previously described, with modifications (Aleshin et al., 2025). Frozen tissue samples were homogenized in an 8-fold volume of ice-cold methanol and incubated on ice for 30 min with shaking at 180 rpm using an Excella E24 shaker (New Brunswick Scientific, Germany). Proteins were precipitated at 20,000 g for 20 min at 4 °C.

The assay is based on the CoASH conversion to succinyl-CoA in the α-KGDHC reaction, which produces NADH, as described by Hammar H and Hammar L (Hammar and Hammar, 1979). Instead of an additional luminogenic reaction, NADH fluorescence (340/470 nm) was directly monitored. The liver methanol extracts (5–20 µL) or CoASH standards in methanol (0–2.5 µM) were mixed with the α-KGDHC assay medium, omitting CoASH. After 10-min equilibration, the reaction was initiated by the addition of 0.003 units of α-KGDHC, enriched from rat heart by polyethylene glycol precipitation (Artiukhov et al., 2020). To correct for the blank, the reactions were also started with samples and standards incubated with 15 mM N-ethylmaleimide to mask all SH-group-containing compounds. The reaction was analyzed until complete CoASH conversion, i.e., until fluorescence plateaued. The resulting CoASH levels in control livers were within the range (50–200 nmol/g fresh weight) reported in the literature (Allred and Guy, 1969; Kato, 1975; Mantel and Butenandt, 1984).

### 2.13 Quantification and statistical analysis

Analyses were performed using GraphPad Prism v10.4.0 and Excel. Data are presented as medians with 95% confidence intervals unless otherwise stated. Box plots show the interquartile range, whiskers show the full range, and dashes indicate medians. Survival distributions were compared using the log-rank (Mantel–Cox) test. For analyses containing more than two survival curves, an overall log-rank test was followed by pairwise log-rank comparisons; pairwise p-values were adjusted using the two-stage Benjamini, Krieger, and Yekutieli false-discovery-rate procedure. A mixed-effects model was used to assess the effects of genotype on body weight over time. For comparisons of three independent groups, one-way ANOVA was used when the stated assumptions were met; otherwise, the Kruskal–Wallis test was used. Each metabolic parameter was analyzed separately using an ordinary two-way ANOVA with genotype and supplementation as factors. To account for testing across the complete metabolomics panel, the omnibus p-values for genotype, supplementation, and genotype × supplementation interaction were adjusted separately across all 76 analyzed metabolic parameters using the two-stage Benjamini, Krieger, and Yekutieli procedure. Pairwise *post hoc* comparisons were additionally adjusted within each metabolic parameter using the same procedure. Statistical significance was defined as p < 0.05 for unadjusted omnibus tests or q < 0.05 after multiple-testing correction.

Animals of different genotypes were tested in random order. Investigators were blinded to genotypes whenever feasible. No animals were excluded unless pre-defined criteria were met (e.g., technical failure, distress). Sample sizes were determined by power calculations or maximized within ethical constraints.

Exact n values, test statistics, and p/q-values are provided in the figures, results, and Supplementary Table S4 and Supplementary Tables S5-S9.

## 3 Results

### 3.1 Clinical case

The patient, a girl, was born to non-consanguineous parents in 2011. She was delivered at term with a birth weight of 4320 g and height of 55 cm (Table 1). From the age of 1 month, the patient began to exhibit psychomotor development delay and frequent vomiting up to 5 times daily. At 9 months of age, laboratory tests revealed elevated levels of C5OH (1.159 μmol/L, normal <0.8 μmol/L) (see Supplementary Material: Supplementary Table S1 for complete data on all blood metabolites analyzed). Urine analysis showed increased excretion of isovalerylglycine (IV), 3-hydroxyisovaleric acid (3-HIVA), 2-methyl-3-hydroxybutyric acid (2-M-3-HB), and suberylglycine (SG) (see Supplementary Table S2 for complete data on all urine metabolites analyzed). Biotinidase (BTD) activity was normal, at 10.8 nmol/min/mL (ref. 4.4–15). A comprehensive examination at 12 months confirmed disharmonic physical development, such as height at the 50th percentile, weight below the 3rd percentile, microcephaly (head circumference below the 10th percentile), and hepatomegaly (liver enlarged by 2 cm). Neurological assessment revealed severe global developmental delay (equivalent to a 7-month-old), muscular hypotonia and dystonia, pseudobulbar syndrome, and vision impairment due to partial optic nerve atrophy. Brain MRI showed reduced cerebral white matter volume.

**TABLE 1:** Clinical characteristics of the patient.

Clinical characteristic	Patient data
Personal and Family History	Proband: Girl, 3rd pregnancy, non-consanguineous marriage. Family History: Elder sister died at 2.5 years with similar clinical manifestations. Elder brother is healthy. Suspected autosomal recessive inheritance
Perinatal history	Antenatal Period: Pregnancy with gestosis (1st trimester), threatened miscarriage (7–8 weeks), and maternal respiratory viral infection at 3 months Birth: Full-term delivery. Birth weight 4320 g (macrosomic), length 55 cm. Apgar score 8–9. Early Neonatal Period: Head control from 1 month, but subsequent severe psychomotor developmental delay and recurrent vomiting (up to 5x/day) were noted
Endocrine phenotype	No overt endocrine abnormalities were reported in the case
Skeletal phenotype	Thoracic kyphosis was noted at 2.5 years. No other specific bone dysplasia or radiographic abnormalities were detailed
Metabolic phenotype	Main features: Recurrent vomiting, triggered by protein load (meat) or intercurrent illness, «sweaty feet » odor Laboratory Hallmarks: 1. *Acylcarnitines and amino acids analysis (Blood):* Consistently elevated C5OH acylcarnitine (1.159–1.594 μmol/L; ref. <0.8) (see Supplementary Table S1 for exact values). 2. *Organic Acid Analysis (Urine):* Elevated 3-hydroxyisovaleric, 3-hydroxybutyric, 2-hydroxyisobutyric, suberic, 2-hydroxyisocaproic, 3-hydroxypropionic acids, and methylcitrate (see Supplementary Table S2 for exact values). 3. *Biochemistry:* Intermittent mild elevations of lactate, pyruvate, CK, AST, LDH. Absent Features: No sustained metabolic acidosis, hypoglycemia, hyperammonemia, or ketosis
Physical findings at 2.5 years overall	General: Developmental delay. Neurological: Muscle hypotonia, spastic paresis (predominantly lower limbs). Skeletal: Thoracic kyphosis. Ophthalmological: Partial optic nerve atrophy
Investigations and Differential Diagnosis	Genetic Testing: No mutations found in HMGCL (3-hydroxy-3-methylglutaryl-CoA lyase deficiency), HLCS (holocarboxylase synthetase deficiency). No pathogenic common mtDNA mutations. Differential diagnosis: Late-onset multiple carboxylase deficiency was excluded based on normal biotinidase activity; 3-Methylcrotonyl-CoA Carboxylase deficiency, and 3-hydroxy-3-methylglutaric aciduria were excluded based on specific urinary metabolite profiles, acylcarnitine analysis
Management and Course	Therapies Tried: 1. Biotin (1 mg/day): No response. 2. Dietary: Leucine restriction (70–100 mg/kg/day), reduced protein (2 g/kg/day), and lipid (25% calories). 3. L-Carnitine (100 mg/kg/day): Short-term benefit (vomiting ceased for 2 months). 4. Symptomatic: Treatment for GERD (Motilium, Phosphalugel). Diagnostic Outcome: Remains unspecified organic acidemia/disorder of branched-chain amino acid metabolism/mitochondrial disease
Current physical findings	died at age 3.5 years

Persistently elevated blood C5OH levels (up to 1.594 μmol/L) and increased urinary excretion of 3-hydroxyisovaleric acid (3-HIVA) and 2-ethyl-3-hydroxyhexanoic acid (2-EHHA) were documented, which are commonly observed in patients with BTD and holocarboxylase synthetase (HLCS) mutations. The extent of C5OH elevation in BTD and HLCS mutations varies across reports. A review summarizing several experimental reports provides a C5OH concentration range for BTD deficiency of 0.2–2.9 μmol/L (median 1.5 μmol/L), while that for HLCS is in the range 0.2–9 μmol/L (median 1.1–2.8 μmol/L across several publications) (Aukes et al., 2025). An extensive differential diagnosis for organic acidemias was performed. Later-onset multiple carboxylase deficiency was ruled out due to normal BTD activity. Other organic acidemias were considered unlikely due to the absence of key clinical features and characteristic blood and urinary markers. No pathogenic mutations were found in the 3-Hydroxy-3-methylglutaryl-CoA lyase (HMGCL) or HLCS genes, and mitochondrial DNA sequencing revealed no pathogenic mutations.

Whole-exome sequencing revealed compound heterozygous R280W and P437Q substitutions in the *SLC5A6* gene, predicted to be pathological based on computational predictions and conservation analyses (see below). Mutations were confirmed by amplifying the genomic DNA fragment and performing Sanger sequencing (Figure 1A). The mother appeared to be heterozygous for the R280W allele, while the father was heterozygous for the P437Q allele. Unfortunately, at the time of this diagnosis, the currently accepted multivitamin supplementation schemes had not yet been published. Since biochemical manifestations, such as elevated blood C5OH and urinary excretion of 3-HIVA, indicated biotin deficiency and a malfunction of the leucine degradation pathway as the primary problem, treatment with biotin (1 mg/day), L-carnitine (100 mg/kg/day), and a diet restricted in leucine and lipids was prescribed. Treatment led to a temporary resolution of vomiting for 2 months. At 19 months, gastroesophageal reflux disease (GERD) was diagnosed and treated. A follow-up examination at 2.5 years confirmed the persistence of developmental delay, muscular hypotonia, spastic paresis, kyphosis, and optic nerve atrophy. Elevated levels of creatine kinase (CK), aspartate transaminase (AST), lactate, and C5OH acylcarnitine were still present. The patient died at the age of 3.5 years.

**FIGURE 1:**
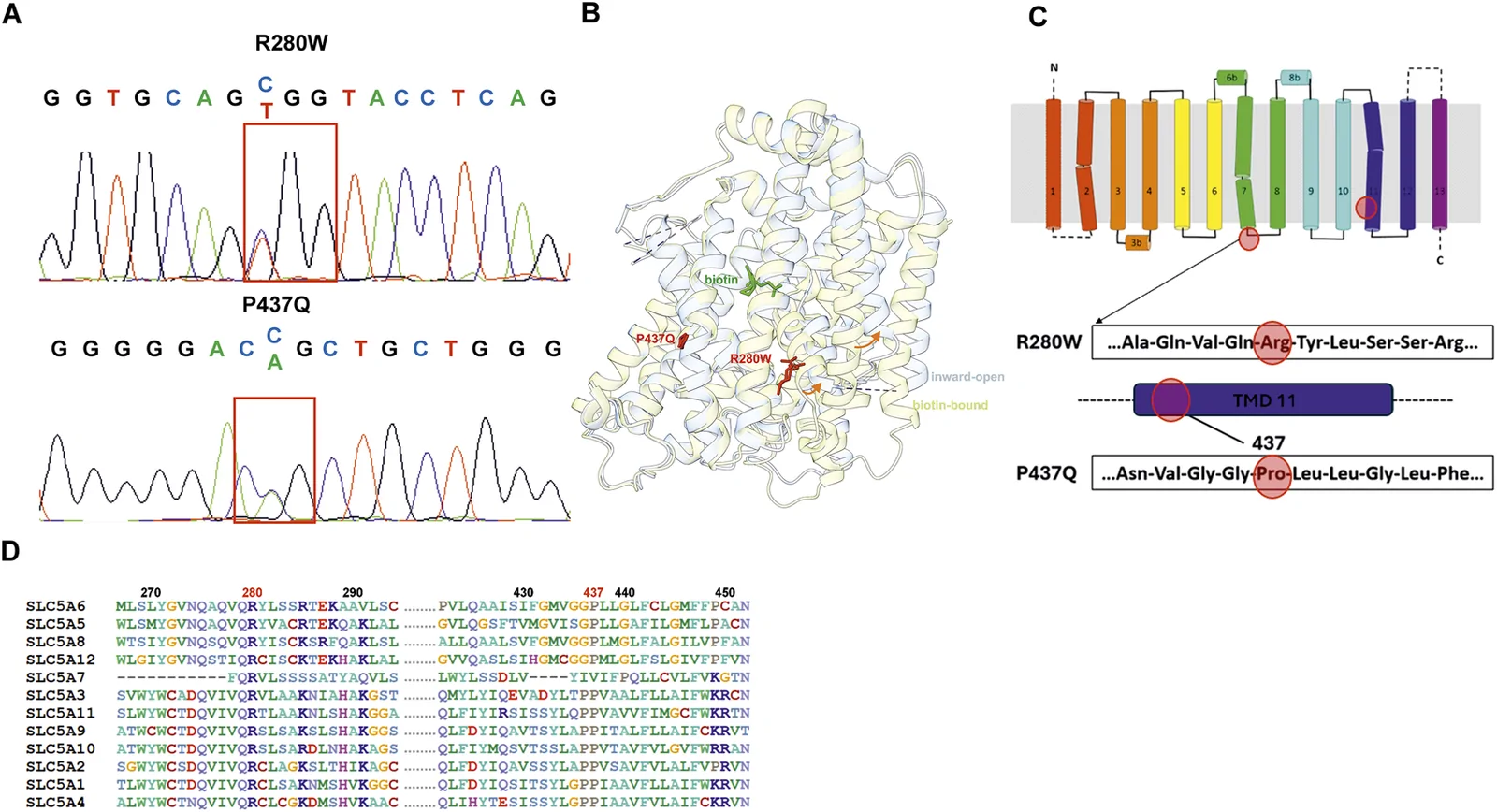
**(A)** Patient’s genotype. Shown are the results of Sanger sequencing of the amplicons corresponding to the regions encoding amino acids 280 (upper panel) and 437 (lower panel) of the patient’s genome. Mutated codons are marked by the red rectangles. **(B)** SMVT protein structure in the biotin-bound (green) and inward-open (blue) state (Zhen et al., 2026), visualized by Chimera (Pettersen et al., 2004). Orange arrows indicate movements accompanying inward opening. Residues mutated in the protein variants reported here are colored red and labeled. Biotin is shown in green and labeled. **(C)** The 13 TMD domains of the SMVT protein are colored in rainbow colors. The red ovals indicate the locations of the substituted amino acids: P437Q in TMD 11 and R280W in the loop between TMD 7 and TMD 8. **(D)** Multiple sequence alignment of human proteins of the SLC5 family (The UniProt Consortium et al., 2023). Red numbers indicate amino acid residues that were substituted in the patient’s genome and studied in this work. Visualized by BioEdit (Hall, 1999).

### 3.2 Potential pathogenicity of the *SLC5A6* ^P437Q^ and *SLC5A6* ^R280W^ mutations

A retrospective analysis of this case prompted us to study the pathogenicity of the *SLC5A6* P437Q and R280W mutations. The missense mutations R280W and P437Q observed in the patient’s *SLC5A6* gene have not been previously reported. To assess computationally their potential pathogenicity, we analyzed the structural context of the affected residues within SMVT protein (Figure 1B). Additionally we studied the conservation of the affected residues in a broader context of related SLC5 family encompassing 12 transporters: biotin, pantothenate and lipoic acid SLC5A6, glucose/galactose SLC5A1, glucose SLC5A2 and SLC5A4, myoinositol SLC5A3 and SLC5A11, iodide SLC5A5, choline SLC5A7, short chain fatty acids SLC5A8 and SLC5A12, mannose, fructose and glucose SLC5A9 and SLC5A10 transporters (Wright, 2013) (Figure 1D).

The R280W mutation, with a high predicted pathogenicity score of 0.933 by the AlphaMissense web resource (Cheng et al., 2023), is of particular interest. The arginine at position 280 is located in an intracellular loop between transmembrane domains (TMDs) 7 and 8 (Figures 1B,C) and is absolutely conserved not only in the orthologous SMVT proteins, but also across the entire family of SLC5 transporters (Figure 1D). Arginine 280 is located close to the cytosolic loop, containing a regulatory PKC phosphorylation site at T287 (Ghosal et al., 2011). Although it seems unlikely, we cannot rule out that the R280W mutation exerts its phenotypic effects by modulating SMVT phosphorylation. More likely, but still hypothetically, the R280W substitution leads to obstruction of the substrate-release gate. Conformational rearrangement leading to the inward-open state of the SMVT transporter involves displacement of helices in direct contact with R280 (Figure 1B) (Zhen et al., 2026). Neighboring Y281 serves as a lid for the cytosolic release path of the transported substrates in similar transporters (Wright, 2013).

The P437Q mutation substitutes the proline residue within the eleventh transmembrane domain (TMD 11) of SMVT (Figures 1B,C). Prolines are uniquely important in transmembrane helices due to their rigid, cyclic structure, which often induces kinks that are crucial for the correct folding and architecture of the transport channel. The proline residue at this position is present not only in all members of SMVT proteins, but also in all SLC5 transporters except the significantly diverged SLC5A7 choline transporter (Figure 1D). The substitution of this structurally critical proline with glutamine, a polar and flexible amino acid, is predicted to alter the helix’s kink angle and stability. The corresponding Pro426 residue of the experimentally determined structure of the related SLC5A5 iodide transporter (Ravera et al., 2022) introduces a kink into TMD11. Given sequence conservation, it is likely that this kink is present in other SLC5 transporters, except possibly SLC5A7. The structure of SLC5A6, which was published while this manuscript was in preparation, revealed that Pro437 indeed introduces a kink in the transmembrane helix (Zhen et al., 2026).

Thus, based on their locations in functionally critical regions, both the R280W mutation, predicted to impede substrate release, and the P437Q mutation, predicted to disrupt transmembrane helix architecture, are computationally predicted to have high pathogenic potential. Their compound-heterozygous status is consistent with the observed severe clinical phenotype of SMVT deficiency.

### 3.3 Creation of the personalized mouse model carrying *Slc5a6* mutations of the patient

To investigate the pathogenic mechanisms of the human compound heterozygous mutations R280W and P437Q in the *SLC5A6* gene, we employed CRISPR/Cas9-mediated genome editing to create two independent mouse lines, each carrying one of the patient-specific mutations (Supplementary Figure S1). The first strain was engineered to carry a point mutation resulting in the P437Q amino acid substitution. Founder animals heterozygous for the P437Q mutation were successfully obtained (Supplementary Figure S1). Subsequent crossing of these heterozygous individuals yielded viable offspring with the expected Mendelian ratio, confirming the successful generation of mice homozygous for the P437Q mutation (Supplementary Figure S1). The second line was designed to carry a substitution leading to the R280W mutation. While heterozygous carriers were viable and fertile, crosses between them failed to produce homozygous R280W offspring (Supplementary Figure S1). Genotyping of progeny from heterozygous crosses revealed a non-Mendelian distribution, with no homozygous mutants, indicating that homozygosity for the R280W mutation results in embryonic lethality. Consequently, this line was maintained in a heterozygous state.

To model the patient’s compound heterozygous genotype, we crossed mice from the two established lines. Specifically, heterozygous *Slc5a6* ^R280W/wt^ mice were crossed with homozygous *Slc5a6* ^P437Q/P437Q^ mice to generate offspring carrying one copy of each mutation (Supplementary Figure S1).

The genotypes of all experimental lines, including the wild-type control group, were rigorously confirmed by Sanger sequencing (Supplementary Figure S1). The wild-type control group was established from the wild-type littermates obtained by crossing heterozygous *Slc5a6* ^P437Q/wt^ mice, ensuring a matched genetic background for all subsequent experiments.

### 3.4 Compound heterozygous *Slc5a6* ^R280W/P437Q^ mutation abruptly reduces survival, while homozygous *Slc5a6* ^P437Q^ exhibits an intermediate phenotype

For the phenotyping of the *Slc5a6* mutant mice, we compared three experimental groups, namely, the heterozygous mice carrying *Slc5a6* ^R280W^ and *Slc5a6* ^P437Q^ mutations, designated *Slc5a6* ^R280W/P437Q^, mice carrying homozygous *Slc5a6* ^P437Q/P437Q^ mutation, designated *Slc5a6* ^P437Q^, and the wild type C57BL/6J, littermates of the animals from other experimental groups carrying the wild type *Slc5a6* genotype and their progeny. A comprehensive survival analysis was conducted across all three experimental groups (Figure 2A). Kaplan-Meier analysis revealed significant differences in survival between genotypes (overall log-rank (Mantel–Cox) test, χ^2^ (2) = 62.83, p < 0.0001). The highest mortality was observed for the *Slc5a6* ^R280W/P437Q^ mice (pairwise log-rank test versus WT, two-stage BKY-adjusted q = 0.0001). Survival of the *Slc5a6* ^P437Q^ mice declines after ca. 2 years of age, whereas the wild-type mice have a significantly longer lifespan (pairwise log-rank test versus WT, two-stage BKY-adjusted q = 0.0001). It is important to note that mice with the *Slc5a6* ^R280W/P437Q^ mutation did not survive beyond 6–7 months of age (Figure 2A), a sad reminder of the patient carrying the same mutations.

**FIGURE 2:**
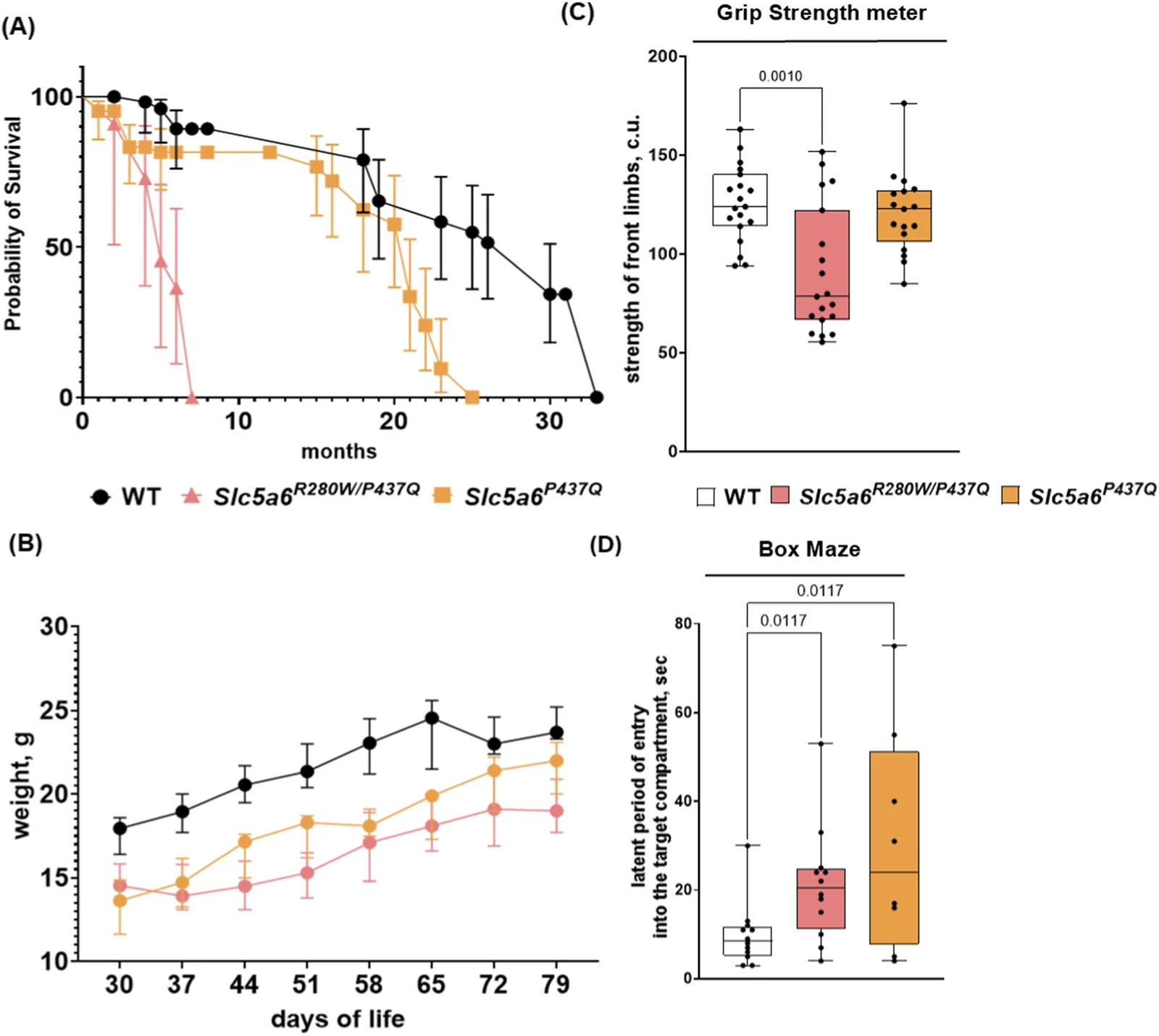
**(A)** Comparison of the survival of male mice with the *Slc5a6* gene mutation with the survival of wild-type mice. WT–wild-type mice (n = 57), *Slc5a6* ^P437Q^–mice with homozygous *Slc5a6* ^P437Q^ mutation (n = 62), *Slc5a6* ^R280W/P437Q^–mice carrying heterozygous genotype *Slc5a6* ^R280W^ and *Slc5a6* ^P437Q^ (n = 16). An overall log-rank (Mantel–Cox) test detected a difference among the three survival curves (χ^2^ (2) = 62.83, p < 0.0001). The three pairwise log-rank comparisons were adjusted using the two-stage Benjamini, Krieger, and Yekutieli procedure; all three comparisons had q = 0.0001. Survival probabilities are presented as Kaplan–Meier curves. **(B)** Comparison of weight gain dynamics in the early life of male mice with *Slc5a6* gene mutations and the WT mice. WT–wild type mice (n = 10), *Slc5a6* ^P437Q^–mice with homozygous *Slc5a6* ^P437Q^ mutation (n = 10), *Slc5a6* ^R280W/P437Q^–mice carrying heterozygous genotype *Slc5a6* ^R280W^ and *Slc5a6* ^P437Q^ (n = 18). Mixed-effects model: χ^2^ value = 102.3 and p < 0.0001. The data are presented as medians ± 95% confidence intervals. **(C)** Comparison of muscle strength based on the maximum grip force of the front paws in the force meter test of mice with a mutation in the *Slc5a6* gene, compared to the muscle strength of wild-type male mice. WT–wild-type mice (C, n = 9), *Slc5a6* ^P437Q^ (C, n = 9), *Slc5a6* ^R280W/P437Q^ (C, n = 7). Kruskal–Wallis statistic: 13.13; p-value: 0,0014. **(D)** Comparison of the associative memory of mice with mutations in the *Slc5a6* gene with the associative memory of wild-type mice in the “Box maze” test. WT–wild-type mice (n = 12), *Slc5a6* ^P437Q^ (n = 12), *Slc5a6* ^R280W/P437Q^ (n = 8). Kruskal–Wallis statistic: 7.291; p-value: 0.0261. Patient reference values are presented above the green line for each metabolite. Data are presented as the interquartile range ± range, with dashes indicating the medians and dots indicating individual values.

### 3.5 Mice with mutations in the *Slc5a6* gene exhibit delayed weight gain

We analyzed body weight gain from 30 to 79 days of life (Figure 2B). Statistical analysis revealed a significant effect of genotype on body weight (mixed-effects analysis: χ^2^ = 102.3, p < 0.0001). Mice with the compound *Slc5a6* ^R280W/P437Q^ mutation had a significantly lower body weight compared to wild-type mice at every time point. Mice carrying the homozygous *Slc*5a6^P437Q^ mutation had reduced body weight compared to wild-type mice, while the degree of growth impairment was less pronounced relative to that of the compound heterozygous mutant mice (Figure 2B).

Thus, both mutations in the *Slc5a6* gene affect weight-gain dynamics, but the phenotype is graded: *Slc5a6* ^P437Q^ mice show a moderate reduction in weight gain, whereas *Slc5a6* ^R280W/P437Q^ mice exhibit a pronounced weight deficit.

### 3.6 A compound heterozygous *Slc5a6* ^R280W/P437Q^ mutation leads to reduced muscle strength and cognitive abilities, while *Slc5a6* ^P437Q^ exhibits an intermediate phenotype

Mice were subjected to a “Grip strength meter” test to assess the maximum grip force of their forelimbs. A significant difference (Kruskal–Wallis statistic = 13.13; p = 0.0014) was found between the group of compound heterozygous *Slc5a6* ^R280W/P437Q^ mice, which demonstrated the lowest grip strength (78 ± 17 arbitrary units; data are presented as median ±95% confidence interval throughout and wild-type mice (119 ± 19 a.u.) (Figure 2C).

To evaluate the influence of the mutations in the *Slc5a6* gene on the cognitive abilities of mice, we applied the “Box maze” test (Figure 2D). In this test, mice have several attempts to learn the location of a shelter cage; after the learning period, the latency to enter the shelter indicates learning efficiency. We showed that mice with *Slc5a6* mutations take longer to find the shelter during the test trial. Latency to enter the shelter was 30 ± 25 s for *Slc5a6* ^P437Q^ mice and 21 ± 13 s for *Slc5a6* ^R280W/P437Q^ mice compared to wild-type mice, with a latency of 10 ± 7 s (Kruskal-Wallis statistic = 7.3, p = 0.026).

In addition to physical strength and cognitive abilities, we analyzed several other behavioral parameters, including general locomotor activity in the “Open field” test, anxiety-like behavior in the elevated plus maze test, the “Marble burying” test, and the hyponeophagia test. The results of these experiments are not definitive and may be found in the Supplementary Material (Supplementary results, Supplementary Figures S2, S3, S5).

Cumulative analysis of all behavioral and physiological tests indicates that mutations in the *Slc5a6* gene result in graded phenotypic changes. The presence of a single mutation (*Slc5a6* ^P437Q^) causes moderately reduced body weight, moderate reduction in muscle strength and associative memory, whereas the compound heterozygous mutation *Slc5a6* ^R280W/P437Q^ leads to a severe multisystemic phenotype, including deficits in muscle strength, pronounced delay in body weight gain, significantly reduced survival, and also impaired associative memory (Supplementary Table S3).

### 3.7 Mutations in the *Slc5a6* gene lead to systemic metabolic imbalance

A patient carrying *SLC5A6* ^R280W/P437Q^ mutations demonstrated deviations from the normal concentration range of certain metabolites (see Supplementary Tables S1-S2). Most notable is the elevated concentration of C5OH in the patient’s blood. We analyzed the concentration of amino acids and acylcarnitines in the blood of mice carrying homozygous *Slc5a6* ^P437Q^ and *Slc5a6* ^R280W/P437Q^ mutations (see Supplementary Table S4, left part, designated as “no biotin” or “placebo”). Only two metabolites’ concentrations were found to increase more than two-fold in the blood of *Slc5a6* ^R280W/P437Q^ mutant mice (Figures 3A,B; left part, designated as “no biotin” or “placebo”); both changes are statistically significant. All metabolites’ concentrations in the blood of mice carrying the homozygous *Slc5a6* ^P437Q^ mutation were within a 1.5-fold range from that of the wild type (Figures 3A,B), in line with the significantly milder phenotype of this mutant. As expected, C5OH levels were found to be elevated most significantly, 3-fold (p-value = 0.0015), in the blood of *Slc5a6* ^R280W/P437Q^ mutant mice compared to the wild type (Figure 3A; left part, designated as “placebo”, i.e., without biotin supplementation; see green line for patient’s level provided for the reference). Concentration of cysteine is 2-fold higher (p value <0.0001) for *Slc5a6* ^R280W/P437Q^ mutant mice (Figure 3B; left part, designated as “placebo”, i.e., without biotin supplementation), whereas no elevation was observed in the patient (Figure 3B, see green line for patient’s level provided for the reference).

**FIGURE 3:**
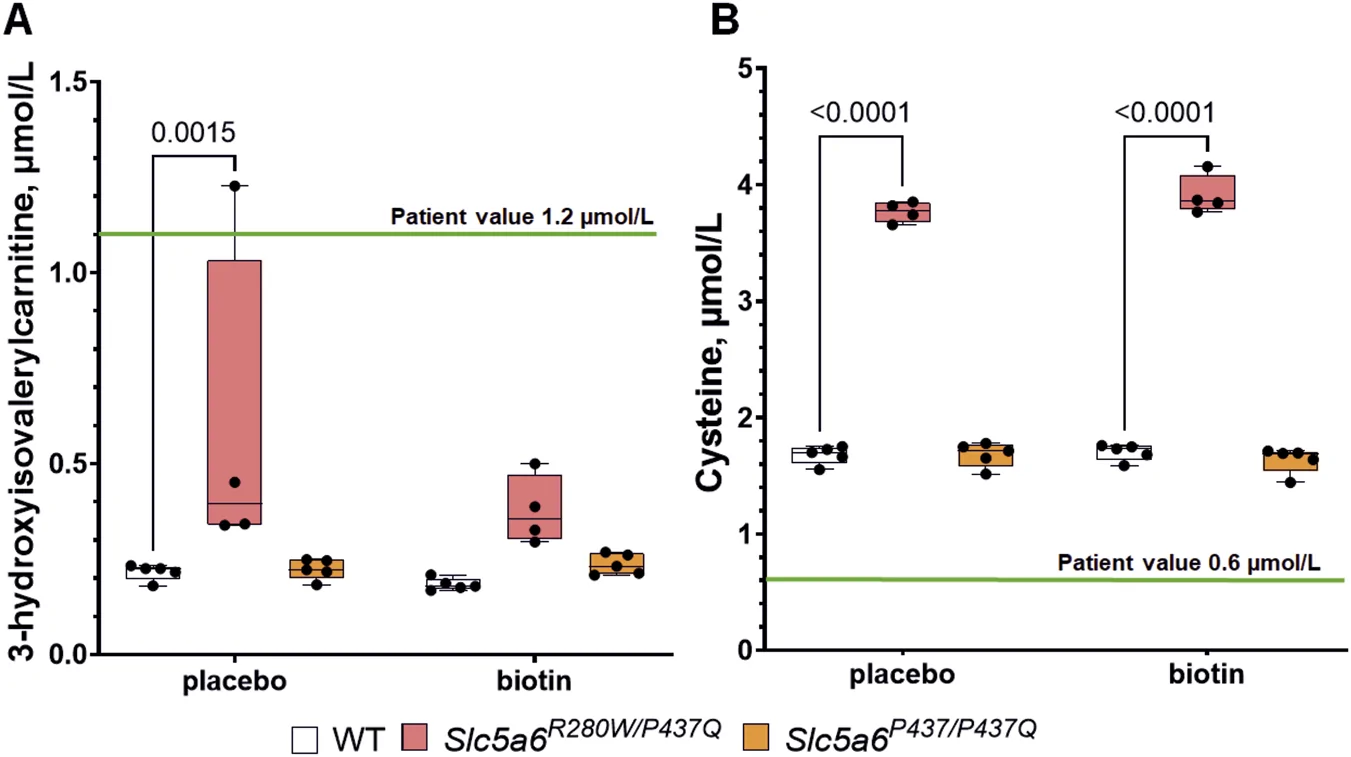
Comparison of 3-hydroxyisovalerylcarnitine (C5OH) **(A)** (Interaction: p = 0.3318, panel q = 0.2113, F (2, 22) = 1.160; Supplement factor: p = 0.2244, panel q = 0.1347, F (1, 22) = 1.563; Genotype factor: p = 0.0026, panel q = 0.0003, F (2, 22) = 7.866) and cysteine **(B)** (Interaction: p = 0.1966, panel q = 0.1469, F (2, 22) = 1.753; Supplement factor: p = 0.3388, panel q = 0.1836, F (1, 22) = 0.9563; Genotype factor: p < 0.0001, panel q < 0.0001, F (2, 22) = 1,188) concentration in blood of male mice with *Slc5a6* gene mutation with survival of wild-type mice upon biotin supplementation. WT–wild-type mice (control n = 5, biotin supplementation n = 5), *Slc5a6* ^*P437Q*^–mice with homozygous *Slc5a6* ^*P437Q*^ mutation (control n = 5, biotin supplementation n = 5), *Slc5a6* ^*R280W/P437Q*^–mice carrying heterozygous genotype *Slc5a6* ^*R280W*^ and *Slc5a6* ^*P437Q*^ (control n = 4, biotin supplementation n = 4). Data are presented as interquartile range ± range of values, with dashes indicating medians and dots indicating individual values. Green bars indicate the values for the patient.

### 3.8 *Slc5a6* mutations influence the availability of cofactors for major metabolic enzymes

SMVT is responsible for transporting biotin, pantothenate, and lipoate across cell membranes. Biotin is covalently attached to the set of four carboxylases, pyruvate carboxylase (PC), Propionyl-CoA carboxylase (PCC), acetyl-CoA carboxylase (ACC), and 3-methylcrotonyl-CoA carboxylase (MCC), where it serves as a carrier for carboxyl group transfer (Figure 4). Lipoic acid is likewise covalently attached to a family of dehydrogenase complexes, such as pyruvate dehydrogenase complex (PDHC), α-ketoglutarate dehydrogenase complex (α-KGDHC), branched-chain α-ketoacid dehydrogenase complex (BCKDC), 2-oxoadipate dehydrogenase complex (OADHC), and glycine cleavage system (GLDC). Pantothenate, when transferred into a cell, is converted to CoA via a set of reactions requiring cysteine and ATP. Thus, mutations in *Slc5a6* might lead to insufficient biotinylation, lipoylation, and CoA synthesis.

**FIGURE 4:**
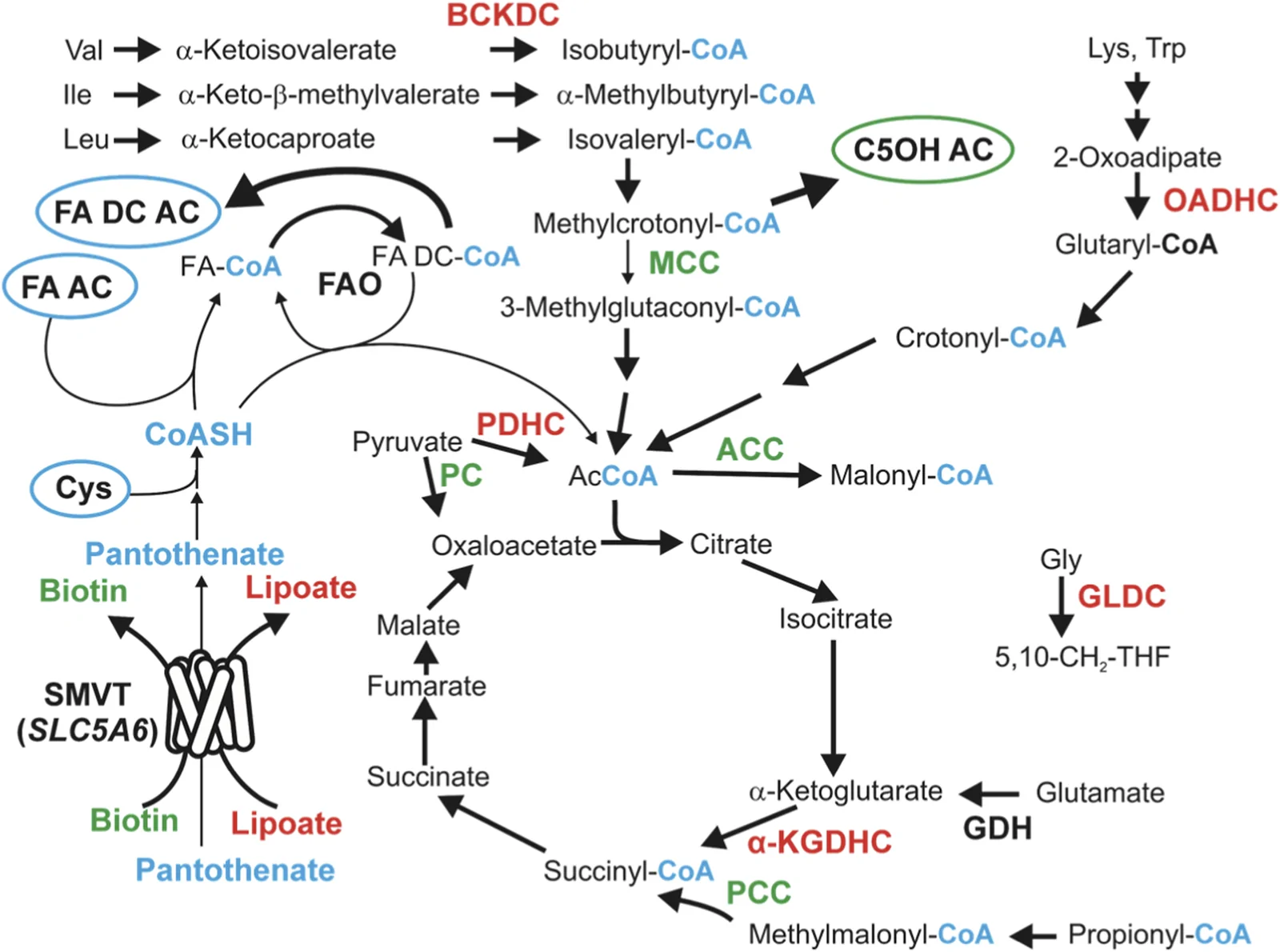
Visualization of major biochemical processes and enzymes involving biotin (green), pantothenate (blue), and lipoate (red). Encircled are metabolites whose concentration was found to be affected by the mutations in SMVT. FA DC AC—Fatty Acid ketoacylcarnitines, FA AC—Fatty Acid Acylcarnitines, FA CoA—Fatty acid Acyl-CoA, FAO—Fatty Acid Oxidation, BCKDC—Branched-chain a-Ketoacid Dehydrogenase complex, MCC—Methylcrotonyl-CoA Carboxylase, PDHC—Pyruvate Dehydrogenase complex, PC—Pyruvate Carboxylase, α-KGDHC—α-Ketoglutarate Dehydrogenase complex, GDH—Glutamate Dehydrogenase, PCC—Propionyl-CoA Carboxylase, OADHC — 2-Oxoadipate Dehydrogenase complex, GLDC—Glycine cleavage system, SMVT (SLC5A6) — Sodium-Dependent Multivitamin Transporter (Solute carrier family member 5A6), Lys—Lysine, Trp—Tryptophan, Gly—Glycine, Ser—Serine, Val—Valine, Ile—Isoleucine, Leu—Leucine, Cys—Cysteine, THF—Tetrahydrofolate, 5,10-CH2-THF—n5, n10-methylene-THF.

To this end, we analyzed the concentration of a free cofactor, CoASH, in the liver extract of both mouse lines, homozygous *Slc5a6* ^P437Q^ and compound heterozygous *Slc5a6* ^R280W/P437Q^ (Figure 5A). In agreement with expectations, we observed a ca. 2-fold reduction in CoASH concentration in the *Slc5a6* ^R280W/P437Q^ mutant (Kruskal-Wallis statistic = 12.66, p = 0.0018) and no change in the *Slc5a6* ^P437Q^ mutant, which has a significantly milder phenotype.

**FIGURE 5:**
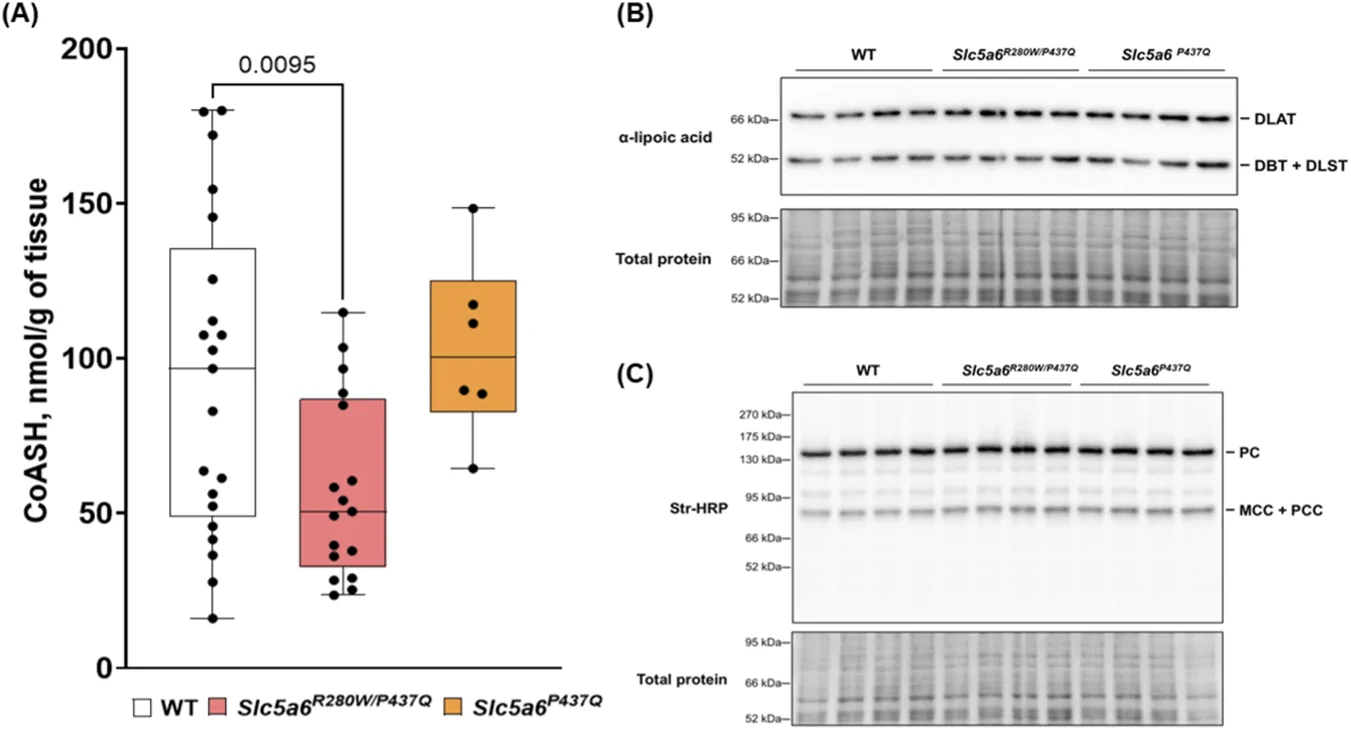
**(A)** Comparison of CoASH levels in liver extracts of male mice with *Slc5a6* gene mutation with that of wild-type mice (Kruskal–Wallis statistic: 12.66; p-value: 0,0018); the average CoASH level of WT mice, set as 100%, was 93.8 ± 11.2 nmol per g FW. WT–wild-type mice (n = 21), *Slc5a6* ^*P437Q*^–mice with homozygous *Slc5a6* ^*P437Q*^ mutation (n = 6), *Slc5a6* ^*R280W/P437Q*^–mice carrying heterozygous genotype *Slc5a6* ^*R280W*^ and *Slc5a6* ^*P437Q*^ (n = 17). Data are presented as interquartile range ± range of values, with dashes indicating medians and dots indicating individual values. **(B,C)** Immunoblotting of total protein extracts from the liver of wild-type and both mutant mouse lines using **(B)** anti-lipoic acid antibodies or **(C)** streptavidin-HRP conjugate. Four biological replicates were used for each mouse type. TCE-based total protein visualization served as a loading control. PC–pyruvate carboxylase, MCC – 3-methylcrotonyl-CoA carboxylase, PCC–propionyl-CoA carboxylase, DLAT–dihydrolipoamide S-acetyltransferase, DBT–dihydrolipoamide branched-chain transacylase E2, DLST–dihydrolipoamide S-succinyltransferase.

To assess the extent of protein biotinylation and lipoylation, we performed immunoblotting of total protein extracts from the liver of both mutant mouse lines and the wild type, using anti-lipoic acid antibodies (Figure 5B) and streptavidin (Figure 5C), which selectively binds biotin. Two bands of lipoic acid-containing proteins, ca. 70 kDa, corresponding to the DLAT component of PDHC, and ca. 50 kDa, corresponding to the DBT and DLST components of BCKDC and α-KGDHC (Figure 5B, see Figure 4 for the scheme). The major biotin-containing protein observed on a blot migrates with mobility matching that of the PC (120 kDa); ACC could not be detected due to the high molecular mass (250–300 kDa), limiting the efficiency of its transfer to the blotting membrane, while MCC (75 kDa) and PCC (72 kDa) could not be resolved (Figure 5C).

None of the major biotinylated and lipoylated enzymes demonstrated a decreased modification in both mutant mouse lines analyzed. This might be expected, since the only major difference between blood acylcarnitine profiles is the elevation of C5OH in the heterozygous *Slc5a6* ^R280W/P437Q^ mice, which is indicative of suboptimal MCC function. Unfortunately, the status of MCC biotinylation could not be resolved by our methods.

The notion that PC biotinylation level is sufficient was corroborated by a direct measurement of liver homogenate PC activity (Supplementary Figure S4A). Neither *Slc5a6* ^R280W/P437Q^ mutant nor *Slc5a6* ^P437Q^ mutant mice demonstrated any significant deviation from the wild-type mice in PC activity. Measurement of α-KGDHC activity (Supplementary Figure S4B) in liver extracts revealed that it was increased in mice with the *Slc5a6* ^R280W/P437Q^ compound mutation compared to the wild type (Kruskal–Wallis statistic = 8.365, p = 0.0153). It might reflect a compensatory activation of α-KGDHC synthesis in mutant mice. No differences in α-KGDHC activity between the *Slc5a6* ^P437Q^ strain and the control were observed. The activity of glutamate dehydrogenase (Supplementary Figure S4C) was used as a control, since we didn’t expect it to depend on any substrates transferred by the SMVT. Accordingly, the enzyme showed no alterations in mutant lines compared to the control.

### 3.9 Excessive biotin supplementation exacerbates some *Slc5a6* mutations’ phenotype

The major difference in the blood metabolome of compound-heterozygous *Slc5a6* ^R280W/P437Q^ mutant mice was an elevation in C5OH. It suggests that biotin deficiency, which reduces MCC activity, is the primary problem caused by the *Slc5a6* ^R280W/P437Q^ mutation. Elevation of C5OH in the patient’s blood prior to whole-exome sequencing led to the prescription of biotin, which proved ineffective. To delve into this problem, we monitored compound heterozygous *Slc5a6* ^R280W/P437Q^ and homozygous *Slc5a6* ^P437Q^ mutant mice, along with wild-type mice, that were fed a diet supplemented with biotin at a high dose (50 mg/kg) roughly matching the one previously used, albeit with pantothenate, for the conditional *Slc5a6* knockout mice (Sabui S et al., 2018). Survival curve (Figure 6A) revealed that biotin supplementation does not increase survival of the heterozygous *Slc5a6* ^R280W/P437Q^ mutant mice, while homozygous *Slc5a6* ^P437Q^ mutant mice demonstrated a substantial reduction of viability if food is supplemented by biotin (overall log-rank (Mantel–Cox) test across all six curves, χ^2^ (5) = 34.47, p < 0.0001; pairwise log-rank tests were adjusted across all 15 comparisons using the two-stage BKY procedure: WT placebo versus biotin, q = 0.1532; *Slc5a6* ^P437Q^ placebo versus biotin, q = 0.0064; *Slc5a6* ^R280W/P437Q^ placebo versus biotin, q = 0.1549).

**FIGURE 6:**
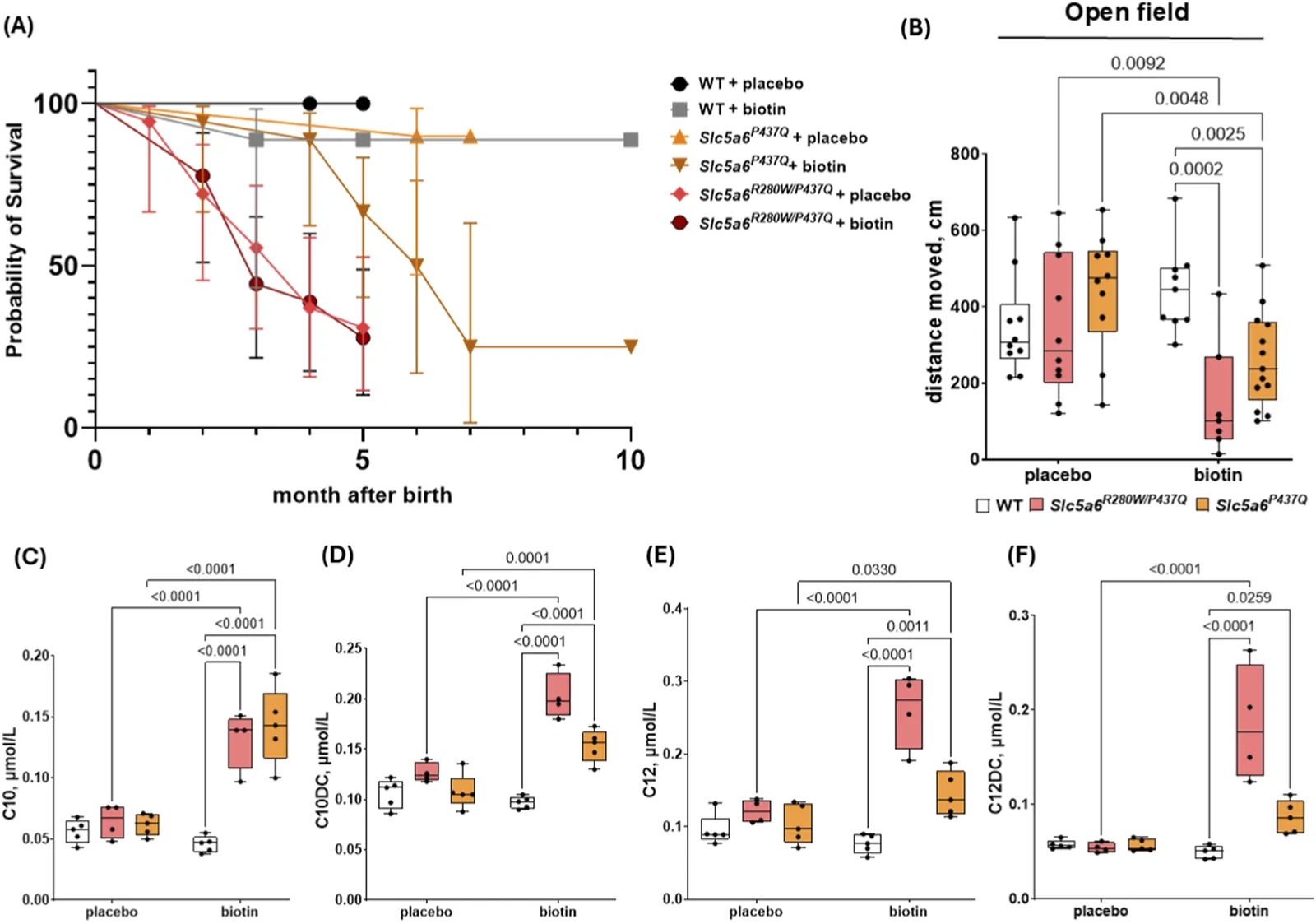
**(A)** Comparison of the survival of male mice with an *Slc5a6* gene mutation with the survival of wild-type mice upon biotin supplementation. WT–wild-type mice (control n = 10, biotin supplementation n = 9), *Slc5a6* ^*P437Q*^–mice with homozygous *Slc5a6* ^*P437Q*^ mutation (control n = 10, biotin supplementation n = 18), *Slc5a6* ^*R280W/P437Q*^–mice carrying heterozygous genotype *Slc5a6* ^*R280W*^ and *Slc5a6* ^*P437Q*^ (control n = 18, biotin supplementation n = 18). An overall log-rank (Mantel–Cox) test detected a difference among the six survival curves (χ^2^ (5) = 34.47, p < 0.0001). All 15 pairwise log-rank comparisons were adjusted using the two-stage Benjamini, Krieger, and Yekutieli procedure. The within-genotype comparisons were: WT placebo versus biotin, q = 0.1532; P437Q placebo versus biotin, q = 0.0064; and R280W/P437Q placebo versus biotin, q = 0.1549. Survival probabilities are presented as Kaplan–Meier curves. **(B)** Comparison of the distance moved in the “Open field” test of male mice with *Slc5a6* gene mutation with wild-type mice upon biotin supplementation. WT–wild-type mice (control n = 10, biotin supplementation n = 9), *Slc5a6* ^*P437Q*^–mice with homozygous *Slc5a6* ^*P437Q*^ mutation (control n = 10, biotin supplementation n = 13), *Slc5a6* ^*R280W/P437Q*^–mice carrying heterozygous genotype *Slc5a6* ^*R280W*^ and *Slc5a6* ^*P437Q*^ (control n = 10, biotin supplementation n = 7) (Interaction: p = 0.0041, F (2, 53) = 6.121; Supplement factor: p = 0.0193, F (1, 53) = 5.828; Genotype factor: p = 0.0118, F (2, 53) = 4.830). Data are presented as interquartile range ± range of values, with dashes indicating medians and dots indicating individual values. **(C–F)** Comparison of decanoylcarnitine (C10) **(C)** (Interaction: p < 0.0001, panel q < 0.0001, F (2, 22) = 18.86; Supplement factor: p < 0.0001, panel q < 0.0001, F (1, 22) = 45.91; Genotype factor: p < 0.0001, panel q < 0.0001, F (2, 22) = 24.81), sebacylcarnitine (C10DC) **(D)** (Interaction: p < 0.0001, panel q < 0.0001, F (2, 22) = 18.34; Supplement factor: p < 0.0001, panel q < 0.0001, F (1, 22) = 42.09; Genotype factor: p < 0.0001, panel q < 0.0001, F (2, 22) = 38.00), dodecanoylcarnitine (C12) **(E)** (Interaction: p < 0.0001, panel q < 0.0001, F (2, 22) = 17.25; Supplement factor: p < 0.0001, panel q = 0.0001, F (1, 22) = 24.83; Genotype factor: p < 0.0001, panel q < 0.0001, F (2, 22) = 30.39) and dodecanedioylcarnitine (C12DC) **(F)** (Interaction: p < 0.0001, panel q < 0.0001, F (2, 22) = 18.75; Supplement factor: p < 0.0001, panel q < 0.0001, F (1, 22) = 30.19; Genotype factor: p < 0.0001, panel q < 0.0001, F (2, 22) = 17.08) concentration in blood of male mice with *Slc5a6* gene mutation compared with wild-type mice upon biotin supplementation. WT–wild-type mice (control n = 5, biotin supplementation n = 5), *Slc5a6* ^*P437Q*^–mice with homozygous *Slc5a6* ^*P437Q*^ mutation (control n = 5, biotin supplementation n = 5), *Slc5a6* ^*R280W/P437Q*^–mice carrying heterozygous genotype *Slc5a6* ^*R280W*^ and *Slc5a6* ^*P437Q*^ (control n = 4, biotin supplementation n = 4). Data are presented as interquartile range ± range of values, with dashes indicating medians and dots indicating individual values.

In agreement with the results of the viability test, weight gain dynamics also revealed that both heterozygous *Slc5a6* ^R280W/P437Q^ and homozygous *Slc5a6* ^P437Q^ mutant mice demonstrate slower weight gain upon supplementation with biotin (Supplementary Figure S5A) (Mixed effects analysis: χ^2^ value = 243.0 and adjusted q-value <0.0001). Influence of biotin supplementation on grip strength (Supplementary Figure S5B (RM ANOVA: Interaction: p = 0.0019, F (2, 53) = 7.168; Supplement factor: p = 0.6985, F (1, 53) = 0.1518; Genotype factor: p = 0.0041, F (2, 53) = 6.160)) is bidirectional for two mutant mouse lines, and thus inconclusive. At the same time, we observed a remarkable negative influence of biotin supplementation on the general locomotor activity of both the compound heterozygous *Slc5a6* ^R280W/P437Q^ and homozygous *Slc5a6* ^P437Q^ mutant mice (Figure 6B (RM ANOVA: Interaction: p = 0.0041, F (2, 53) = 6.121; Supplement factor: p = 0.0193, F (1, 53) = 5.828; Genotype factor: p = 0.0118, F (2, 53) = 4.830); Supplementary Figure S5C (RM ANOVA: Interaction: p = 0.0037, F (2, 53) = 6.229; Supplement factor: p = 0.0161, F (1, 53) = 6.182; Genotype factor: p = 0.0124, F (2, 53) = 4.778)).

What might be the reason for the negative influence of biotin supplementation on the fitness of *Slc5a6* mutant mice? To get an insight into the biochemical basis of this phenomenon, we analyzed blood amino acid and acylcarnitine profiles of heterozygous *Slc5a6* ^R280W/P437Q^ and homozygous *Slc5a6* ^P437Q^ mutant mice fed a biotin-supplemented chow (Supplementary Table S4, columns “+ biotin”). Expectedly, biotin supplementation resulted in a decrease in the C5OH concentration, which was elevated for heterozygous *Slc5a6* ^R280W/P437Q^ mice (Figure 3A, right part, designated as “biotin”). However, blood cysteine concentration for heterozygous *Slc5a6* ^R280W/P437Q^ mice did not decrease upon biotin treatment (Figure 3B, right part, designated as “biotin”). Most notably, the addition of biotin into the food of mice resulted in a dramatic increase in several fatty acid acylcarnitines and their ketoacyl derivatives’ concentrations for both heterozygous *Slc5a6* ^R280W/P437Q^ and homozygous *Slc5a6* ^P437Q^ mutant mice (Figures 6C–F; Supplementary Table S5, columns “+ biotin”).

## 4 Discussion

In this work, we retrospectively describe a new case of human sodium multivitamin transporter deficiency (SMVTD; MIM 618973) caused by previously unpublished compound heterozygous mutations R280W and P437Q in the *SLC5A6* gene, and the creation and study of a personalized mouse model of this hereditary disease. In recent years, a substantial number of published cases of SMVT transporter deficiency have been reported worldwide (Table 2), which were unavailable at the time of the clinical case described and at the start of our work to create the mouse model. To our knowledge, the complete set of published cases of *SLC5A6* mutations in patients is described in detail in the supplementary discussion (Supplementary Material) and summarized in Table 2. In brief, clinical cases associated with *SLC5A6* mutations can be roughly divided into severe, early-onset, potentially lethal disease, SMVTD (MIM 618973), and a much milder, childhood-onset biotin-responsive peripheral motor neuropathy, COMNB (MIM 619903).

**TABLE 2:** Clinical cases with *SLC5A6* mutations, described in the literature.

*SLC5A6* genotype	Clinical presentation	Treatment and result	Age of patient	Citation
Compound Heterozygous: R94X and R123L	Multisystemic disorder: failure to thrive, microcephaly, developmental delay, immunodeficiency, severe gastro-esophageal reflux, osteoporosis, and bone fractures	Patient responded favorably to supplemental pharmacologic doses of biotin, pantothenic acid, and lipoic acid	“Young child”	(Subramanian et al., 2017)
Compound Heterozygous: V141fs and R400T	Sibling pair with early infantile-onset, progressive neurodegenerative phenotype, developmental delay, and epileptic encephalopathy	Triple-vitamin (biotin, pantothenate, lipoic acid) therapy resulted in clinical improvement in neurocognitive and neuromotor function	Onset at 12–14 months	(Byrne et al., 2019)
Compound Heterozygous: V141fs and Q622fs	Presented with hypoglycemia, severe metabolic acidosis, feeding problems, and poor weight gain, mimicking biotinidase deficiency	An increased biotin dose and the introduction of pantothenic acid resulted in a striking clinical improvement	Presented at 17 months old	(Schwantje et al., 2019)
Compound Heterozygous: (two missense mutations, unspecified)	Cyclical vomiting, faltering growth, perioral rash, and bilateral optic atrophy	Weekly vitamin replacement (Biotin, Dexpanthenol, α-lipoic acid) led to significant improvement; vomiting and rashes resolved	Presented at 13 months, alive at 2 years old	(Rupasinghe and Onyeador, 2021)
Compound Heterozygous: c.1005 + 1G>A and Q622fs	Mild phenotype with failure to thrive, frequent vomiting, metabolic acidosis with hypoglycemia, and mild osteopenia	Treatment with biotin, pantothenic acid, and lipoic acid resulted in clinical improvement across all aspects of the disease	Feeding difficulties and vomiting were reported soon after birth	(Hauth et al., 2022)
Compound Heterozygous: R94X and Y162C	Motor neuropathy	Biotin, pantothenic, and lipoic acid supplementation resulted in improvement	Onset at 7 years	(Holling et al., 2022)
Homozygous: S429G	Motor neuropathy in four individuals from two families	Biotin treatment was initially prescribed to 3 patients with no positive outcome. Later, supplementation with biotin, pantothenic acid, and lipoic acid resulted in improvement in 3 of 4 patients	Onset at 6–13 years	(Holling et al., 2022)
Compound Heterozygous: M61K and P349L	Primary immunodeficiency with very low immunoglobulin levels. Recurrent infections, sparse hair, skin erosion	Biotin replenishment ameliorated the immunodeficient phenotypes	Onset at 1 year old. Older sister died at 3 years old	(Hsieh et al., 2023)
Homozygous: L566fs	Three patients from one family with a severe disorder: developmental delay, sensory polyneuropathy, optic atrophy, recurrent infections, and intestinal pseudo-obstruction	Two untreated patients died. A third patient treated with early supplementation with biotin and pantothenic acid had a stabilized clinical course	Two died in early infancy. The third patient’s age is not specified	(Montomoli et al., 2023)
Compound Heterozygous: S74F and Q214H	A mild psychomotor development delay and brain cysts, which were spontaneously resorbed	No treatment required	Brain cysts diagnosed at 1 month	(Utsuno et al., 2024)
Homozygous: c.460–19T>G (intronic variant)	Four patients from three families with severe mixed axonal and demyelinating sensory motor neuropathy, systemic optic atrophy, and severe cyclic vomiting	Supplementation treatment is reported to be effective, but specific outcomes for these patients are not detailed	Onset at age 3–4 years	(Mansour-Hendili et al., 2024)
Compound Heterozygous: S429G and c.393 + 2T>C	Infections, diarrhea, cytopenia, and at the age of 8–10 years, developed upper limb motor neuropathy	Biotin, pantothenic, and lipoic acid supplementation resulted in improvement	Onset at age 3 years	(Riley et al., 2024)
Homozygous: S429G	Weakness	Multivitamin therapy was prescribed, but the outcome is unknown	14 years of age	(Riley et al., 2024)
Homozygous: P437 L	Refusal to eat and vomiting, severe hypogammaglobulinemia, and lactic acidosis Cardiac insufficiency	Biotin and pantothenic acid supplementation resulted in improvement	Onset at the age of 5 months	(Van Vyve et al., 2024; Fullerton et al., 2026)
Compound Heterozygous: P70fs and L11fs	A child with developmental delay, microcephaly, neurological symptoms, skin lesions, and frequent upper respiratory tract infections	Treatment with increased doses of oral biotin, pantothenic acid, and lipoic acid resulted in significant clinical improvement	1 year	(Kononets et al., 2024)
Compound Heterozygous: (unspecified)	Fetal onset with ventriculomegaly; postnatal developmental regression and acute metabolic shock	High-dose biotin and pantothenic acid led to dramatic improvements in systemic symptoms, but significant psychomotor disability remained	Developed metabolic shock at 6 months of age	(Ogawa et al., 2025)
Compound Heterozygous: R94X and F522S	Cyclic vomiting, sparse hair, recurrent infections, and visual impairment	Not reported	Onset at 2 years old	(Pi et al., 2025)
Compound Heterozygous: C443Y and c.1539_1544 + 14del (exon + intron)	Developmental delay, spasticity, easy fatigue, sparse hair, and recurrent infections in childhood; later, seizures, eczematous skin lesions, vomiting, cardiomyopathy, osteopenia, and optic atrophy	Multivitamin therapy relieved most symptoms	Onset at 2 years old, some symptoms much later, up to 28 years	(Pi et al., 2025)
Compound Heterozygous: G272R and Q622fs	Autism spectrum disorder, intellectual disability, and epilepsy	Multivitamin therapy was prescribed, but the outcome is unknown	Symptoms developed at 2–28 years old	(Walimbe et al., 2025)
Homozygous: R253W	Dilated cardiomyopathy, developmental delay, respiratory failure.	Patient 1 died at age 2 years Patient 2 received a donor heart at 7 years old. Biotin, pantothenic acid, and lipoic acid supplementation started at age 14 resulted in improvement	Patient 2 developed symptoms at 9 months	(Fullerton et al., 2026)
Compound Heterozygous: R280W and P437Q	Developmental delay, muscle hypotonia, spastic paresis (predominantly lower limbs), thoracic kyphosis, partial optic nerve atrophy	Biotin (1 mg/day): No response. Leucine restriction (70–100 mg/kg/day), reduced protein (2 g/kg/day), and lipid (25% calories). Short-term benefit (vomiting ceased for 2 months). Treatment for GERD (Motilium, Phosphalugel)	Presented at 9 months, 2.5 years deceased	This study

COMNB manifests at ages 6–13 years as distal limb axonal neuropathy, most frequently with upper-limb weakness and fatigability. Remarkably, all patients with this form of the disease originated from Kerala, India, and 6 of 7 patients carried the same S429G allelic variant, of which 5 were homozygous (Holling et al., 2022; Riley et al., 2024). The SMVT residue S429 is located in transmembrane helix 11, the same as the one carrying the P437 residue, whose mutation is reported in this work. Both S429 and P437 residues face helix 12, away from the internal cavity of the transporter. Both the S429G (Holling et al., 2022; Riley et al., 2024) and P437Q mutations alone (mouse model, this study) resulted in relatively mild symptoms. However, yet another mutation at the same residue, P437L, which is hypothetically more disruptive to the transporter’s tertiary structure, resulted in an early-onset severe disease (Van Vyve et al., 2024). Other cases of mild pathology caused by *SLC5A6* mutations were described for compound heterozygous S64F/Q214H mutations, which manifested as mild psychomotor delay and spontaneously resorbed brain cysts (Utsuno et al., 2024), and for the Y162C mutation, which was presented as a compound heterozygote with a nonsense R94X mutation (Holling et al., 2022).

The severe form of SMVT deficiency manifests itself at ca. 1 year of age with failure to thrive, frequent vomiting and gastroesophageal reflux, developmental delay, neuropathy and microcephaly, optic nerve atrophy, skin rash, immunodeficiency with frequent infections, and osteoporosis with bone fractures (Table 2, Supplementary discussion). Recently, dilated cardiomyopathy has been described in a number of patients carrying SMVT mutations, a phenotype further supported by cardiac-specific conditional inactivation of the *SLC5A6* gene in a mouse model (Fullerton et al., 2026). The majority of reported *SLC5A6* mutations lead to the severe form of the disease. Three cases were reported for the same amino acid-to-stop codon substitution, R94X (Subramanian et al., 2017; Holling et al., 2022; Pi et al., 2025); all were compound heterozygotes with missense mutations. Several frameshift variants, such as V141fs (Schwantje et al., 2019), L11fs and P70fs (Kononets et al., 2024), L566fs (Montomoli et al., 2023), and Q622fs (Schwantje et al., 2019; Walimbe et al., 2025), have been published; the latter leads to an extended protein form. Three intronic variants (Hauth et al., 2022; Mansour-Hendili et al., 2024; Riley et al., 2024) and one combined intronic and exonic variant (Pi et al., 2025) were also found. No patients are known to have one mutant and one wild-type allele, indicating that a single functional SLC5A6 gene copy is sufficient for a healthy life and that no dominant toxic “gain of function” mutations have been documented for this gene. It is likely that if one mutation is inactivating, the severity of clinical outcome should be determined by the functionality of the second allele; e.g., R94X/Y162C mutations (Holling et al., 2022) demonstrate a milder phenotype.

Only two publications reported cases of apparent complete inactivation of the gene (Table 2): compound heterozygous frameshift mutations at amino acid residues 11 and 70 (Kononets et al., 2024) and a homozygous frameshift mutation at residue 566 (Montomoli et al., 2023). The latter frameshift truncates the C-terminal region beyond the protein’s transmembrane domain and does not necessarily result in complete inactivation. Another reported case of potentially complete *SLC5A6* inactivation described a frameshift at codon 11 (Kononets et al., 2024). We hypothesize that, in this case, a leaky scanning mechanism may increase the probability of translation initiation at downstream AUG codons, e.g., Met21 or Met27, as supported by ribosome profiling data on the transcriptome-wide distribution of initiating ribosomes (Michel et al., 2014). In our view, it is unlikely that complete inactivation of the *SLC5A6* gene is compatible with viability in humans, since mouse models (Hsieh et al., 2023; CAM-SU, 2026; Fullerton et al., 2026) (this study) have demonstrated that individuals homozygous for inactivating *Slc5a6* mutations are embryonic lethal. Assessment of the pathogenicity of SMVT missense mutations is rarely possible from clinical data alone, since most clinical cases involve heterozygous mutations.

The two missense mutations reported here have very different consequences when present separately. Mutation P437Q is viable in a homozygous state in mice and demonstrated elevated mortality only at an age of more than 2 years. It also has minimal phenotypic manifestations. Proline 437 is conserved in all but one SLC5 family transporter, and according to available structures and structure modeling (Figures 1B–D), it facilitates transmembrane segment 11 bending. It is likely that the mutation slightly destabilizes the SMVT protein structure but still allows it to function at a suboptimal level. Another reported mutation, R280W, is embryonic lethal and thus likely to inactivate the function. Residue R280 is totally conserved not only in the orthologous SLC5A6 proteins, but also among the paralogous transporters (Figure 1D). According to the model of the SLC5 transporter family mechanism of action (Wright, 2013), arginine 280 is located next to Y271 and F79 (Y259 and F67 in the available structure PDB:7UV0 of related iodide transporter SLC5A5), acting as an internal gate lid, opening to release the transported substrates into the cytoplasm (Ravera et al., 2022). In a recently published study of the structures of the SMVT in several functional states, the R280 residue contacts the helices undergoing conformational changes upon formation of the inward-open state (Zhen et al., 2026). Substitution of R280 with a bulky tryptophan might either block the pathway of substrate release or destabilize the structure of SLC5A6 due to a steric clash. The severe phenotype of compound heterozygous R280/P437Q mutations might be explained by the presence of only one copy of partially functional SLC5A6 and a complete inactivation of the second allele.

The treacherous aspect of the pathology caused by *SLC5A6* gene mutations is that its clinical and biochemical manifestations mimic those of other hereditary metabolic diseases. The most frequent biochemical manifestation of SMVT deficiency is elevated blood C5OH, which has been reported in all cases for which analysis has been performed (this study, Schwantje et al., 2019; Hauth et al., 2022; Riley et al., 2024; Fullerton et al., 2026). In addition, in some cases (Schwantje et al., 2019; Riley et al., 2024) but not in others (this study, patient data) (Hauth et al., 2022; Fullerton et al., 2026), elevated blood concentrations of C3 acylcarnitine were noted. In fact, in our study, *Slc5a6* ^R280W/P437Q^ and *Slc5a6* ^P437Q^ mutant mice demonstrated even somewhat (less than 2-fold) lower blood C3 acylcarnitine concentration (Supplementary Table S4).

Biotin deficiency, albeit incomplete, is likely to result first in MCC malfunction and, second, in suboptimal PCC activity. Whether C3 would be elevated in a particular clinical case might depend on other factors, such as genetic background beyond *SLC5A6* or diet. Mutations in holocarboxylase synthetase (HLCS), which is responsible for attaching biotin to PC, ACC, MCC, and PCC, also result in elevations in C5OH and C3, sometimes leading to misdiagnosis (Donti et al., 2016). SMVTD may also be misdiagnosed as an MCC deficiency, resulting in a rise in C5OH (Rutledge et al., 1995; Baykal et al., 2005). Mutations in PCC result specifically in the elevation of C3 in blood samples (Wongkittichote et al., 2017), so it may more readily be distinguished from the *SLC5A6* mutant phenotype. Biotinidase (BTD) deficiency is also manifested by an increase in blood C5OH concentration (Strovel et al., 2017; Funghini et al., 2020). Due to the much higher prevalence of BTD deficiency in the population (Karachaliou and Livaniou, 2024), misdiagnosis of *SLC5A6* mutations as *BTD* mutations is possible when genetic testing and enzymatic assays are unavailable.

Rare cases of ACC deficiency (Lou et al., 2021) are easily distinguished from *SLC5A6* mutations by acylcarnitine profiles, since ACC catalyzes the first step in fatty acid synthesis; thus, ACC mutations decrease blood fatty acid carnitine concentrations. Pyruvate carboxylase (PC) deficiency is extremely rare (Habarou F et al., 2015) and manifests as lactic acidosis and hyperammonemia (Ahmad et al., 1999; Habarou et al., 2015). Acylcarnitine profiles are not very informative in the diagnosis of *PC* mutations.

In the present case, C5OH was elevated to 1.159 μmol/L at 9 months and later to 1.594 μmol/L, with a clinical cutoff of <0.8 μmol/L. These values exceed the reference range and reasonably match those for C5OH levels in BTD (0.2–2.9 μmol/L) and HLCS (0.2–9 μmol/L) disorders (Aukes et al., 2025). Some reports claim substantially higher C5OH concentrations, such as 3–15 μmol/L for HLCS deficiency (Ling et al., 2023).

To sum up this part, *SLC5A6* mutations result in elevated blood C5OH and, more rarely, C3, which might be misdiagnosed as mutations in *BTD*, *HLCS*, *MCC*, and, less likely, *PCC*. Biochemical changes caused by transporter mutations do not resemble those seen in PC or ACC deficiency. Dissimilarity in biochemical features also indicates that biotin scarcity primarily affects the activities of MCC and PCC, while PC and ACC compete for available biotin.

SMVT transports not only biotin but also pantothenate and lipoic acid (Wang et al., 1999), thus mutations in *SLC5A6* might result in a shortage of these metabolites. In mammalian cells, lipoic acid is covalently attached to PDHС, α-KGDHС, BCKDС, OADHC, and GLDC. PDHС deficiency leads to elevated lactate, but normal acylcarnitine profiles (Ma et al., 2021). α-Ketoglutarate dehydrogenase deficiency does not alter acylcarnitine profile (Yap et al., 2021). Inactivating mutations in GLDC cause glycine encephalopathy, characterized primarily by elevated glycine levels (Applegarth and Toone, 2001). Mutations in genes encoding BCKDС subunits are diagnosed by an increase in concentrations of branched-chain amino acids Val, Ile, and Leu and corresponding branched-chain α-keto acids (Tangeraas et al., 2023), while OADHС (DHTKD1) deficiency results in elevated 2-aminoadipate and 2-oxoadipate (Danhauser et al., 2012; Stiles et al., 2016). None of these features were detected for clinical cases of the *SLC5A6* mutations, including the one reported here. It is thus likely that the transporter deficiency does not affect lipoate availability, at least as a primary mechanism of pathogenicity. This notion is consistent with the presence of the lipoic acid synthesis pathway in mammals (Morikawa et al., 2001; Pourcel et al., 2020), which is independent of the SMVT transporter.

Pantothenate is a precursor of CoA, a universal carrier of acetyl, fatty acyl, and many other carboxyl-containing substrates of cellular oxidation (Figure 4). The conversion of pantothenate to CoASH is a multistep process that requires cysteine and ATP (Figure 4). With some reservations, the biochemical consequences of pantothenate deficiency might resemble those of pantothenate kinase deficiency, the first enzyme in the pathway of CoA synthesis. A major biochemical manifestation of PANK2 deficiency, one of the human pantothenate kinases, is the accumulation of cysteine (Pourcel et al., 2020), which is used in the next step of CoA synthesis (Barritt et al., 2024). Cysteine accumulation was observed in our mouse model of SMVT deficiency, but not in a patient. Knockout of a paralogous enzyme gene, *PANK1*, resulted in the accumulation of fatty acid carnitines, although this effect was not observed in patients deficient in PANK2. It is likely that particular SMVTD biochemical manifestations might resemble biotin or pantothenate deficiency, depending on the availability of the corresponding vitamins in one’s diet.

In addition to describing a new clinical case of SMVTD caused by two previously undescribed SLC5A6 amino acid substitutions, we created the first viable personalized mouse SMVTD model, suitable for recapitulating the severe and mild forms of SLC5A6 deficiency using heterozygous *Slc5a6* ^R280W/P437Q^ and homozygous *Slc5a6* ^P437Q^ mutant mice, respectively. Several mouse models with genetically altered *Slc5a6* have been previously reported. Since complete inactivation of the transporter gene is lethal, the first published model used the Cre/lox system to generate an intestinal *Slc5a6* cKO (Ghosal et al., 2013). Such tissue-specific knockout resulted in decreased body weight, inflammation of the intestine, and death at the age of 6–10 weeks. Supplementation with biotin and pantothenate normalized weight dynamics and relieved inflammation (Sabui et al., 2018). Recently, a cardiac-specific *Slc5a6* knockout mouse model has been created to study dilated cardiomyopathy, which has been described in patients carrying homozygous R253W or P437L mutations (Fullerton et al., 2026). The model recapitulated both biochemical defects (i.e., elevated blood C5OH levels) and cardiac-specific fibrosis, cardiac enlargement, conduction abnormalities, and ultrastructural defects observed in patients. Likewise, supplementation with biotin and pantothenic acid in mice alleviated the symptoms. Cardiac-specific *Slc5a6* knockout also resulted in a statistically significant elevation of several fatty acyl carnitines in blood plasma and a reduction of pantothenate and its derivatives, including CoA-glutathione, in heart tissue, suggesting a fatty acid oxidation defect (Fullerton et al., 2026). These results nicely complement our data, while the peculiar differences might be explained by genetic context, i.e., tissue-specific KO vs. whole-body mutations that reduce functionality.

Another mouse model of Slc5a6 deficiency has been created by introducing a patient-derived M61K mutation (Hsieh et al., 2023). However, this mutation appeared to be embryonically lethal in a homozygous state, and the immune phenotype, which was the focus of that study, could only be recapitulated by subjecting a heterozygous *Slc5a6* ^WT/M61K^ mouse to a biotin-restricted diet. Unfortunately, the second allelic variant of the same patient, P349L, has not been introduced to mice. While tissue-specific knockouts are undoubtedly useful, they cannot fully recapitulate pathogenesis, since the affected cell type would be entirely devoid of the SMVT, which is not the case in patients, and systemic-level pathology characteristic of patients will not be adequately modeled.

The creation of heterozygous *Slc5a6* ^R280W/P437Q^ and homozygous *Slc5a6* ^P437Q^ mutant mouse lines allowed us to observe the characteristic rise in C5OH for the severe form of the pathology. We also observed a decrease in CoASH concentration and an increase in cysteine concentration, which likely reflects pantothenate deficiency. A decrease in CoA concentration results in statistically significant alteration of CoA-dependent processes, such as fatty acid oxidation, only after biotin treatment. It is likely a consequence of further competition between pantothenate and biotin for the SMVT, resulting in depletion of the residual, perhaps fatty acid-bound, CoA pool.

A retrospective analysis of the clinical case presented here indicates that treating SMVT deficiency with sole biotin supplementation does not yield a positive clinical outcome. This case is not the only one in which SMVTD was initially treated with biotin. A case reported in 2019 (Schwantje et al., 2019) illustrates that initial treatment with biotin, which was prescribed on the basis of biochemical but not genetic data, resulted in limited improvement, while motor development delay, feeding problems, poor weight gain, chronic diarrhea, and vomiting were not relieved by biotin. Only supplementation with pantothenate and biotin resulted in an overall improvement. Three patients with *SLC5A6* S429G mutation (Holling et al., 2022) were initially treated solely with biotin, with no clinical improvement, whereas the addition of pantothenate and lipoic acid supplementation resulted in significant improvements in two of the three individuals.

We recapitulated the processes that occur during attempts to treat both severe and mild SMVT deficiency with biotin in our mouse model. We demonstrated that biotin treatment does not extend the survival of mice carrying the severe disease-associated *Slc5a6* ^R280W/P437Q^ genotype. We observed increased mortality in mice homozygous for the *Slc5a6* ^P437Q^ mutation when treated with biotin. Weight gain and locomotor activity both showed a negative trend in heterozygous *Slc5a6* ^R280W/P437Q^ and homozygous *Slc5a6* ^P437Q^ mutant mice treated with biotin. Analysis of blood amino acids and acylcarnitines showed a partial decrease in the initially elevated C5OH concentration following biotin treatment in heterozygous *Slc5a6* ^R280W/P437Q^ mice; this was the only good news. At the same time, both mouse lines showed increased concentrations of several fatty acids and ketoacyl carnitines, reminiscent of a deficiency in fatty acid β-oxidation (Yamada et al., 2019). Cysteine concentration stayed high upon the biotin treatment of the heterozygous *Slc5a6* ^R280W/P437Q^ mice. The likely interpretation of these findings is that biotin, at excessive concentrations, competes for the SMVT transport capacity, which is itself limited by mutations. Such competition among substrates for SMVT has previously been described (Uchida et al., 2015; Pourcel et al., 2020), although for the specific mutant variants of the transporter reported in our work, this competition model requires future experimental validation.

## 5 Conclusion

Our study provides evidence of the danger of misdiagnosing SMVT malfunction as a biotin supply deficiency, as in BTD, HLCS, or MCC mutations; however, similar symptoms and biochemical findings may be observed. We created a personalized mouse model of the patient carrying previously undescribed *SLC5A6* mutations, R280W and P437Q, and demonstrated that biotin treatment alone is insufficient, if not dangerous, for treating this disease, unlike a treatment with a combination of biotin, pantothenate, and lipoic acid, resulting in a substantial relief of the symptoms in the majority of published cases. A straightforward recommendation for clinicians and medical geneticists is to analyze the *SLC5A6* gene sequence in all cases of elevated C5OH not explained by mutations in other known biotin pathway genes, and, if the SLC5A6 gene is indeed affected, consider prescribing a combination of biotin, pantothenate, and lipoic acid.

## Data Availability

The original contributions presented in the study are included in the article/Supplementary Material; further inquiries can be directed to the corresponding authors. The mouse lines described are available upon request to the Lomonosov Moscow State University biocollection of genome-edited mice (https://micecoll.msu.ru/, 2025).
