# Supplementary material for: Sodium-dependent multivitamin transporter deficiency caused by novel SLC5A6 mutations: a clinical case and personalized mice model study
Source: Front Cell Dev Biol. 2026 Oct 9;14:1925753. doi: 10.3389/fcell.2026.1925753 (PMC13690556; doi:10.3389/fcell.2026.1925753)
Supplement: Supplementary file 1 [file DataSheet1.pdf]

Supplementary Material

1 Supplementary methods

1.1 Phenotyping

1.1.1 Elevated Plus Maze test

The test space is placed approximately 1 m above the floor and consists of two sets of opposing arms (30×5 cm): two dark arms are enclosed by 15 cm-high walls, and the remaining two arms are open (OpenScience, Russia). The mice are given the choice to spend time in open, unprotected arms or in enclosed, protected arms. The approach-avoidance conflict between the innate desire to explore and the fear of open spaces elicits behaviors correlated with increased physiological stress indicators (Bailey and Crawley, 2009).

1.1.2 Hyponeophagia test

The food was removed from the cage hopper the evening 18 hours before testing. The test was conducted the next morning, and their food was replaced as soon as testing was finished. The chosen hyponeophagia test apparatus, as well as the row of cages in which mice from the group home cage are placed individually before and during testing, were set up. This avoids increased anxiety in the remaining mice in the group cage, which can occur if you progressively remove mice from a group, as well as the social transmission of food preferences. It also assists in standardizing tests between group- and singly housed animals and ensures that the animals are optimally alert. The mouse was placed in the test apparatus. The latency before first contact with food, total time while consuming food, and contact with food were measured (Deacon, 2011; Perepelkina and Poletaeva, 2022).

1.1.3 Marble burying test

The conditional defensive digging of glass balls allows assessment of the animal's anxiety level. In this test, we compare the number of balls buried in the bedding (more than 2/3 of their diameter) with that of the control group to assess (Angoa-Pérez et al., 2013).

1.1.4 Assays of Enzyme Activities

The enzyme activities were measured in liver homogenates prepared as described previously. α-KGDHC and GDH were assayed using 2- 2.5 µl of liver homogenate diluted 5-fold with 50 mM MOPS, pH 7.0 (Aleshin et al., 2025). Activities of these enzymes were measured against blanks using identical assay mixtures that omitted 2-ketoglutarate. PC activity was measured using 2 µl of liver homogenate, diluted 10-fold in 50 mM MOPS, pH 7.0, according to (Warren and Tipton, 1974; Fahien et al., 1993), with the following modifications. The blank medium contained 150 mM Tris-lactate buffer, 5 mM $MgCl_2$, 0.12% BSA, 7 mM pyruvate, 0.23 mM NADH, 0.88 U/mL malate dehydrogenase, pH 8.8 adjusted with KOH. The assay medium also included 15 mM $NaHCO_3$, 1 mM ATP, and 50 µM acetyl-CoA. All reactions were initiated by mixing homogenates with assay buffers at room temperature, and analysis was performed at 25 °C. Activities were blank-corrected using assay media omitting 2-ketoglutarate or pyruvate and expressed as µmol of product formed per min per g of tissue fresh weight, using a calibration curve for NADH, linear at 0.01–1 nmol/well interval, for the fluorometric assays or its molar extinction coefficient of 6220 $M^{-1} \times cm^{-1}$ for the spectrophotometric ones.

1.2 Quantification and statistical analysis

All statistical analyses were performed using GraphPad Prism (version 10.4.0) and Excel. Specific software packages used for individual analyses are indicated in the corresponding figure legends and methods sections. Throughout the manuscript, data are presented as medians ± 95% confidence intervals unless otherwise specified.

In figure panels, individual data points are shown where appropriate, with box plots indicating the interquartile range, whiskers representing the full range, and dashes indicating medians. Survival probabilities are presented as Kaplan–Meier curves. Statistical significance was defined as $p < 0.05$ or $q < 0.05$ after correction for multiple comparisons. Survival distributions were compared using the log-rank (Mantel–Cox) test. For analyses containing more than two survival curves, an overall log-rank test was followed by pairwise log-rank comparisons; pairwise p-values were adjusted using the two-stage Benjamini, Krieger, and Yekutieli false-discovery-rate procedure. A mixed-effects model was used to assess body weight over time. For comparisons of three independent groups, one-way ANOVA was used when the stated assumptions were met; otherwise, the Kruskal–Wallis test was used. Experiments with genotype and supplementation as independent factors were analyzed using ordinary two-way ANOVA. Each metabolic parameter was analyzed separately using genotype and supplementation as factors. To account for testing across the complete metabolomics panel, the omnibus p-values for genotype, supplementation, and genotype × supplementation interaction were adjusted separately across all 76 analyzed metabolic parameters using the two-stage Benjamini, Krieger, and Yekutieli procedure. Pairwise post hoc comparisons were additionally adjusted within each metabolic parameter using the same procedure. Raw omnibus p-values, panel-wide q-values, F statistics, and within-parameter post hoc q-values are reported in Supplementary Tables S5–S9.

The exact number of experimental units (n) for each analysis is provided in the corresponding figure legends. In all experiments, n represents the number of individual animals per experimental group, unless otherwise specified. For metabolite analyses, group sizes are additionally reported in Supplementary Table S4; group sizes for other biochemical assays are given in the corresponding figure legends.

Animals were randomly assigned to experimental groups based on genotype. Where possible, experiments included age- and sex-matched animals to control for potential confounding variables.

For behavioral tests, animals were tested in a random order to minimize potential circadian or order effects. Investigators conducting behavioral assessments and biochemical analyses were blinded to genotype whenever feasible.

All animals meeting the specified genotype criteria were included in the survival and phenotyping analyses. No animals were excluded from the primary analyses unless specified in the corresponding figure legends. For survival studies, sample sizes were maximized within the constraints of animal availability and ethical considerations.

All statistical details, including exact n values, test statistics, and p/q-values, are provided in the figure legends, results section, and supplementary materials (Supplementary tables 5-9).

2. Supplementary results

2.1 Anxiety-like behavior of the *Slc5a6* mutant mice

To rule out the influence of reduced baseline locomotor activity on the results of other tests, the "Open field" test was conducted. No differences were found between male mice of different genotypes in either the total distance traveled (Kruskal-Wallis statistic = 4.4, p=0.1), time spent in the center of the arena (Kruskal-Wallis statistic = 0.47, p=0.8), or grooming time (Kruskal-Wallis statistic = 4.6, p=0.1) (Supplementary figure S2A-C). Thus, physical activity levels in this test in mice with the *Slc5a6* mutation are comparable to those observed in wild-type mice, allowing us to exclude reduced mobility as a contributor to the results of other behavioral tests.

For the "Elevated Plus Maze" test, we analyzed the following parameters: time spent in the open arms, time spent in the center, and grooming time (Supplementary figure S2D-F). We found significant differences in time spent in the open arms among mice of different genotypes (Kruskal-Wallis statistic = 14.3, p = 0.0008). Mice with the *Slc5a6*$^{R280W/P437Q}$ mutations spent more time on the open arms (60 ± 23.2 seconds), whereas *Slc5a6*^{P437Q} mice spent the least time (12 ± 13.4 seconds),

while the wild-type mice demonstrated an intermediate value (30 ± 14.5 seconds) (Supplementary figure S2D). Differences were also observed in the time spent in the central zone (Kruskal-Wallis statistic = 8.3, p = 0.016). *Slc5a6*$^{R280W/P437Q}$ mice spent more time in the center (169 ± 47 seconds) compared to wild-type mice (93 ± 42 seconds) (Supplementary figure S2E). Differences between the count of head dippings on the open arms (Kruskal-Wallis statistic = 7.3, p=0.0264) were statistically significant for mice *Slc5a6*^{P437Q} (19 ± 9) in comparison with the wild-type mice (31 ± 11) (Supplementary figure S2F). However, we could not provide a rationale for this observed effect.

In the hyponeophagia test (Supplementary figure S3A-C), when presented with a piece of cheese, a novel food unfamiliar to the mice, a significant difference was found between genotypes in the amount of cheese consumed (Kruskal-Wallis statistic = 8.1, p=0.017): *Slc5a6*$^{R280W/P437Q}$ mice consumed the least (0.057 ± 0.04 grams), whereas *Slc5a6*^{P437Q} mice (0.12 ± 0.04 grams) and wild-type mice (0.113 ± 0.06 grams) consumed comparable amounts of the novel food. At the same time, the latency period to approach the novel food (Kruskal-Wallis statistic = 1.39, p=0.5) and the time spent interacting with the novel food (Kruskal-Wallis statistic = 4.5, p=0.1) did not differ between groups. This indicates a reduction in novel food consumption rather than an increase in avoidance. We also studied anxiety levels in mice from different groups using the marble burying test (Supplementary figure S3E). We found that the number of marbles buried did not differ between the groups (Kruskal-Wallis statistic = 0.88, p = 0.6).

3. Supplementary discussion

Since 2017, a substantial number of clinical cases have been reported in which illness has been caused by *SLC5A6* mutations. Here we present, to our knowledge, a complete description of published cases of SMVT deficiency in chronological order.

Compound heterozygous mutations R94X and R123L resulted in microcephaly and brain changes, developmental delay, immunodeficiency, gastro-esophageal reflux, osteoporosis, and pathologic bone fractures. Acylcarnitine profiles have not been reported. The patient benefits from high-dose supplementation with biotin, pantothenic acid, and lipoic acid (Subramanian et al., 2017).

The second case reported for *SLC5A6* mutations described two siblings with compound heterozygous mutations V141fs and R400T. Patients developed severe neurologic symptoms and cyclic vomiting. Acylcarnitine profiles have not been reported in this case either. Before recognition of the causative mutations, one patient died due to acute gastrointestinal hemorrhage. After *SLC5A6* mutations were identified, a surviving sibling was prescribed high-dose supplementation with biotin, pantothenic acid, and lipoic acid (Byrne et al., 2019), resulting in significant improvement.

Vomiting, psychomotor retardation, acidosis, osteoporosis with bone fractures, along with elevated blood C5OH acylcarnitine and urine 3-OH-isovaleric acid were observed in a child carrying *SLC5A6* V141fs and Q622fs mutations (Schwantje et al., 2019). Before whole-exome sequencing, because of the resemblance in biochemical and clinical manifestations to biotinidase deficiency, biotin supplementation was prescribed. However, only partial effects were observed. Sporadic vomiting and diarrhea, feeding problems, and motor development delay remained until the diagnosis was clarified by whole-exome sequencing. Pantothenate supplementation was added to biotin supplementation, resulting in increased appetite, resolution of diarrhea, and improved growth.

Frequent vomiting and feeding difficulties were reported in a patient carrying a splicing donor-site mutation, c.1005+1G>A, and a frameshift mutation, Q622fs, in the C-terminal region of the *SLC5A6* gene (Hauth et al., 2022). Remarkably, in this case, biochemical analysis revealed an elevated

concentration of C5OH-acylcarnitine in the blood. In this case, as well, supplementation with biotin, pantothenate, and lipoic acid ceased vomiting and reversed other clinical symptoms.

Five individuals from three families, likely from Kerala, India, with *SLC5A6* pathogenic mutations were reported by an international consortium in 2022 (Holling et al., 2022). Late-onset neurologic symptoms, such as distal upper-limb weakness, were detected at ages 8-14 years. A homozygous S429G mutation was identified in four patients from two families, while the remaining patient carried compound heterozygous mutations R94X and Y162C. Three patients were initially treated with biotin, but without marked improvement. When supplemented with pantothenate and lipoic acid in addition to biotin, patients showed significant improvement.

Recurrent infections, sparse hair, and dyskeratotic skin erosion, along with a substantial decrease in immunoglobulin levels, have been described for a carrier of compound heterozygous *SLC5A6* mutations M61K and P349L (Hsieh et al., 2023). Interestingly, in this case, supplementation with biotin alone resulted in the relief of skin dyskeratosis and normalization of antibody levels.

A mild psychomotor development delay and brain cysts, which were spontaneously resorbed, were found in three patients with a mild form of SLC5A6 deficiency caused by a compound heterozygous mutation S74F and Q214H (Utsuno et al., 2024).

Three patients were products of marriages between two couples of siblings who originated from the same village in Italy (Montomoli et al., 2023). The first patient demonstrated developmental delay, hypotonia, muscle wasting, strabismus, nystagmus, optic atrophy, alopecia, dermatitis, recurrent diarrhea, vomiting, and infections. Biochemical analysis revealed lactic acidosis and increased 3-hydroxyisovaleric acid in urine. The patient died at age 3. Patient 2 experienced dermatitis, diarrhea, cardiomyopathy, and visual impairment. Multivitamin treatment relieved most symptoms. Patient 3

experienced infections, diarrhea, dermatitis, and reflux and died at the age of 17 months. All three patients carried a homozygous *SLC5A6* mutation L566fs (Montomoli et al., 2023).

Frequent infections, developmental delays, low weight gain, anemia, microcephaly, and non-growing hair have been described in a patient with a heterozygous *SLC5A6* mutation, P70fs and L11fs (Kononets et al., 2024). The symptoms were accompanied by an elevated blood concentration of C4DC/C5OH and increased urinary excretion of 3-OH-isovaleric acid. Biotin was initially prescribed to the patient as a sole therapy with limited outcomes. Following the genotype analysis, biotin supplementation was combined with pantothenic and lipoic acids, resulting in marked improvement.

A patient from Kerala, India, was diagnosed in Australia; the sequencing revealed a compound heterozygous mutation S429G and c.393+2T>C (Riley et al., 2024). The intronic mutation resulted in retention of intron 3 or use of cryptic exon 3 splice donors. The patient suffered frequent infections, diarrhea, cytopenia, and, at the age of 8-10 years, developed upper limb motor neuropathy. Acylcarnitine profiles revealed an elevation of C5OH AC. Biotin, pantothenic acid, and lipoic acid supplementation resulted in improvement. An unrelated patient who shared an Indian origin was found to carry a homozygous S429G variant (Riley et al., 2024). Multivitamin therapy was likewise prescribed to the patient.

A homozygous *SLC5A6* P437L mutation was found in a patient with refusal to eat and vomiting, severe hypogammaglobulinemia, lactic acidosis with elevated urine excretion of 3-OH-isovaleric acid, but normal blood C5OH AC level (Van Vyve et al., 2024). Complete recovery has been observed following combined biotin and pantothenate treatment.

Four patients of Algerian origin had a homozygous *SLC5A6* mutation c.460–19T>G, duplicating the splicing acceptor site (Mansour-Hendili et al., 2024). Patients demonstrated walking disorder,

neuropathy, optic atrophy, and cyclic vomiting. Three patients who received multivitamin therapy survived, and their condition stabilized.

Compound heterozygous *SLC5A6* mutations D610fs and Q152P have been described for a patient who was diagnosed at age 6 months with hypogammaglobulinemia, lactic acidosis, cerebral atrophy, bacterial infections, skin rash, gastroesophageal reflux disease, and left ventricular hypertrophy (Ogawa et al., 2025). Treatment with biotin and pantothenic acid resulted in a dramatic improvement.

A set of three cases in which SMVT dysfunction resulted in dilated cardiomyopathy, elevated blood C5OH acylcarnitine, and 3-OH-isovaleric acid in the urine has recently been described (Fullerton et al., 2026). Reported patients carried either homozygous R253W or homozygous P437L mutations.

Early onset (2 y.o.) disease caused by *SLC5A6* mutations R94X/F522S with cyclic vomiting, sparse hair, recurrent infections, and vision impairment has been described for a Korean patient (Pi et al., 2025). Another Korean patient with developmental delay, spasticity, easy fatigue, sparse hair, and recurrent infections in childhood. Later, seizures appeared, and the patient demonstrated eczematous skin lesions, vomiting, cardiomyopathy, osteopenia, and optic atrophy at the age of 28 years. Compound heterozygous mutations C443Y and 20-bp deletion variants, including six exonic and 14 intronic nucleotides, c.1539_1544+14del, were found in the *SLC5A6* gene of the patient (Pi et al., 2025). Multivitamin therapy with biotin, lipoic acid, and pantothenic acid relieved most symptoms.

Autism spectrum disorder, intellectual disability, and epilepsy with elevated urine excretion of lactic acid, ketones, 3-HIV, glutaric and dicarboxylic acids, hyperammonemia, and acidosis of blood were diagnosed in a 25-year-old female with *SLC5A6* mutation variants G272R/Q622fs (Walimbe et al., 2025).

Overall, the range of symptoms associated with mutations in the *SLC5A6* gene is very wide. The harsh edge of it manifests as early onset severe life-threatening illness, sodium-dependent multivitamin transporter deficiency (SMVTD; MIM 618973) affecting brain, immunity, vision, and leading to frequent vomiting, recurrent infections, and hemorrhages. On the contrary, the mild form of the SMVT deficiency is childhood-onset biotin-responsive peripheral motor neuropathy (COMNB; MIM 619903). It manifests at an age of ca. 10 years or later as distal limb weakness due to muscle denervation.

4. Supplementary Figures and Tables

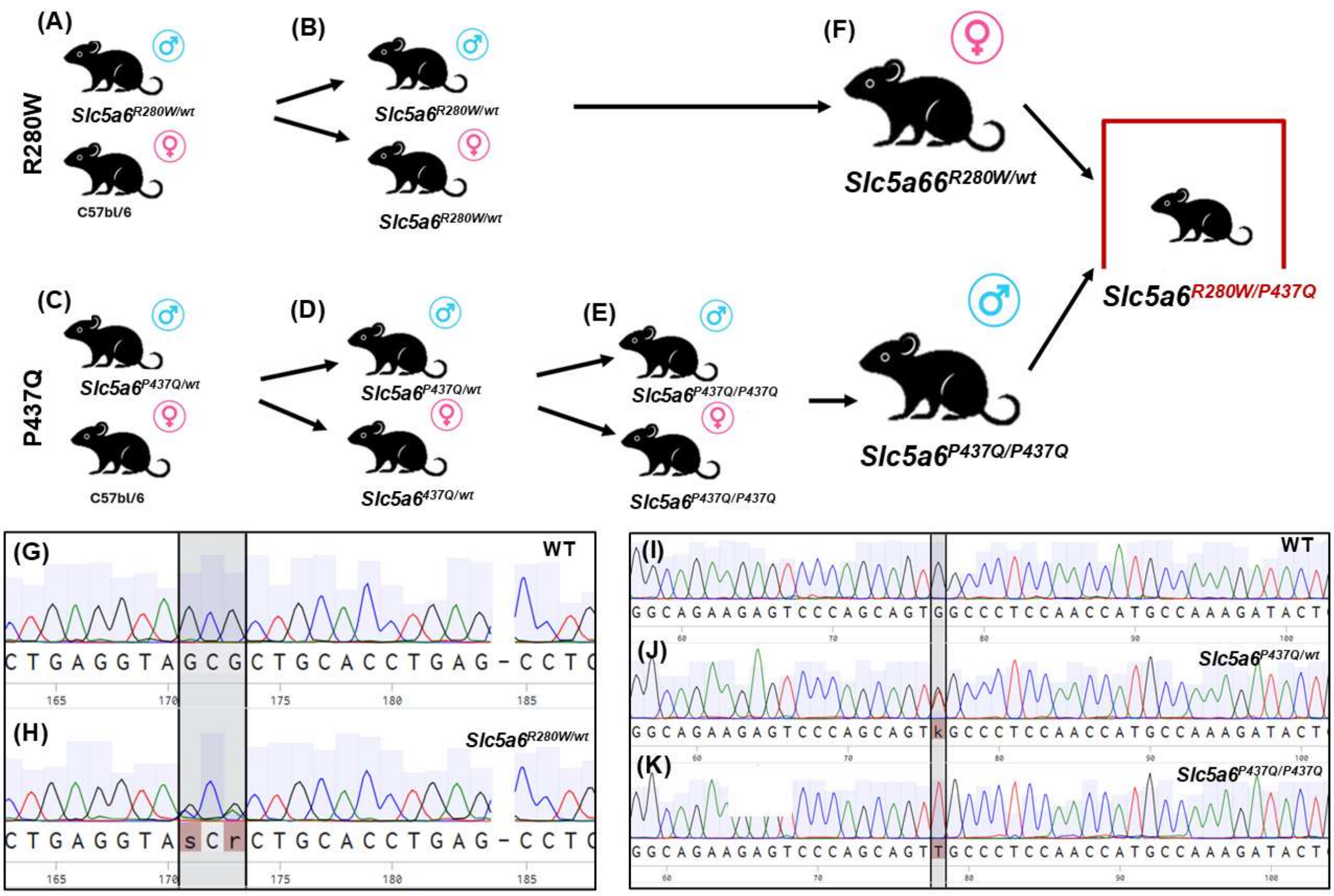

Supplementary Figure 1. Scheme for creating compound heterozygous mice carrying the R280W and P437Q missense mutations in the *Slc5a6* gene. (**A**) Mating between a male carrying the R280W missense mutation in a heterozygous state and a female C57Bl/6 wild-type mouse. (**B**) Maintenance of the mouse line carrying the R280W missense mutation in a heterozygous state. (**C**) Mating between a male carrying the P437Q missense mutation in a heterozygous state and a female C57Bl/6 wild-type mouse. (**D**) Mating between mice heterozygous for the P437Q missense mutation.

(**E**) Mating between mice carrying the P437Q missense mutation in a homozygous state. (**F**) Creation of the compound heterozygous mouse line carrying both the R280W and P437Q missense mutations in the *Slc5a6* gene by crossing a female carrying the R280W missense mutation in a heterozygous state and a male carrying the P437Q missense mutation in a homozygous state. (**G-K**) Sequence of the *Slc5a6* allelic variants created. **(G**) Sequence of the wild-type murine *Slc5a6* gene in the region coding for amino acid 280. (**H**) Sequence of the heterozygous WT/R280W mutant murine *Slc5a6* gene in the region coding for amino acid 280. (**I**) Sequence of the wild-type murine *Slc5a6* gene in the region coding for amino acid 437. (**J**) Sequence of the heterozygous WT/P437Q mutant murine *Slc5a6* gene in the region coding for amino acid 437. (**K**) Sequence of the homozygous P437Q mutant murine *Slc5a6* gene in the region coding for amino acid 437. Visualized by (https://benchling.com/).

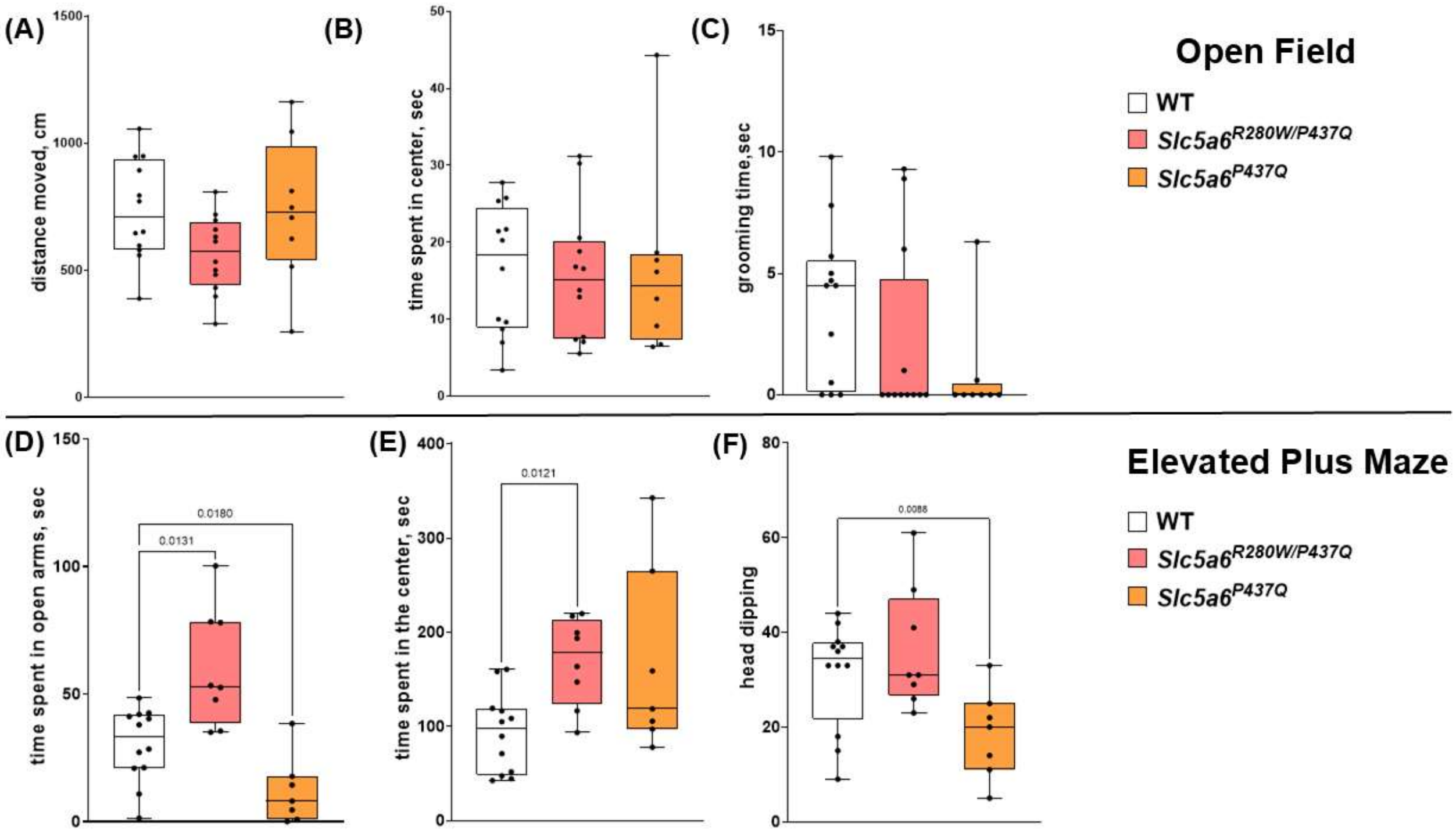

Supplementary Figure 2. (**A-C**) Comparison of motor activity in the "Open field" test based on (**A**) total distance moved (Kruskal-Wallis statistic: 4.419; p value: 0.1098), (**B**) time spent in the center of the arena (Kruskal-Wallis statistic: 0.4736; p value: 0.7892), and (**C**) grooming time of mice with a mutation in the *Slc5a6* gene compared to wild-type mice (Kruskal-Wallis statistic: 4.640; p value: 0.0983). WT – wild-type mice (n=12), $Slc5a6^{P437Q}$ – mice with the homozygous $Slc5a6^{P437Q}$ mutation (n=12), $Slc5a6^{R280W/P437Q}$ – heterozygous mice with the $Slc5a6^{R280W}$ and $Slc5a6^{P437Q}$ alleles (n=8). The data are presented as interquartile range ± range, with dashes indicating medians and dots indicating individual values. (**D-F**) Comparison of the following indicators of anxious behavior in the "Elevated plus maze" test of mice with a mutation in the Slc5a6 gene with those of wild-type mice: (**D**) time spent in the open arms (Kruskal-Wallis statistic: 14.29; p value: 0.0008), (**E**) time spent in the center (Kruskal-Wallis statistic: 8.308; p value: 0.0157), and (**F**) head dipping (Kruskal-Wallis statistic: 7.270; p value: 0.0264),. WT – wild-type mice (n=12), $Slc5a6^{P437Q}$ – mice with the $Slc5a6^{P437Q}$ mutation (n=8), $Slc5a6^{R280W/P437Q}$ – heterozygous mice with the $Slc5a6^{R280W}$ and

Slc5a6^{P437Q} alleles (n=7). The data are presented as interquartile range ± range, with dashes indicating medians and dots indicating individual values.

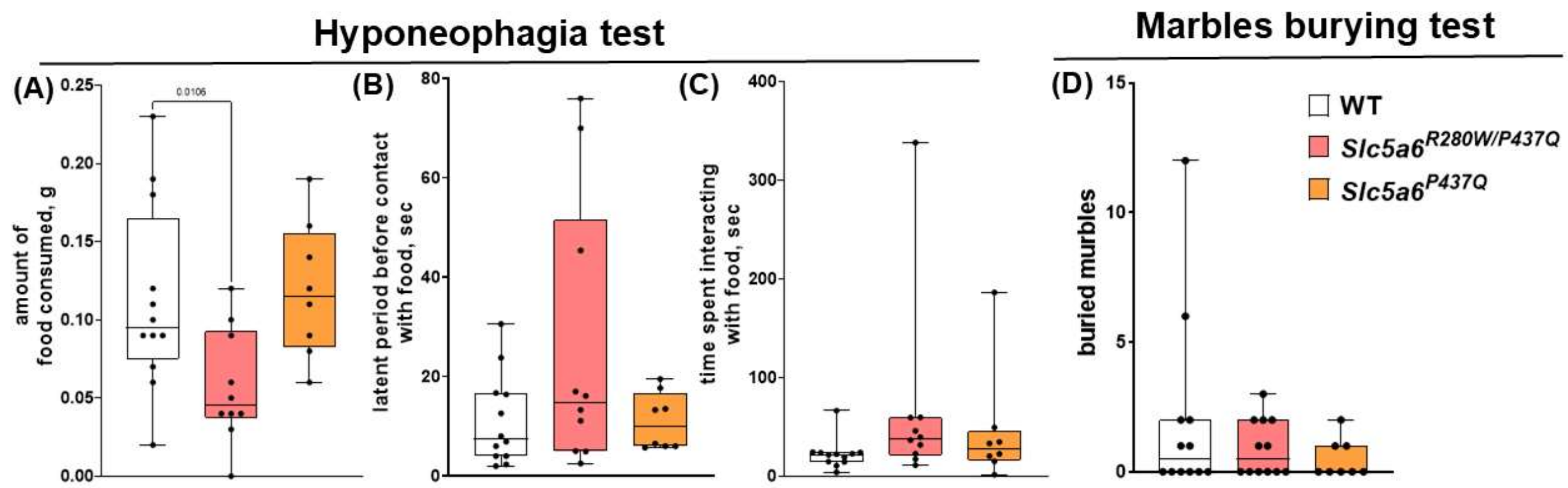

Supplementary Figure 3. (**A-C**) Comparison of the following parameters in the hyponeophagia test of mice with a mutation in the *Slc5a6* gene with those of wild-type mice: (**A**) amount of new food consumed (Kruskal-Wallis statistic: 8.115; p value: 0.0173), (**B**) latent period before approaching the new food (Kruskal-Wallis statistic: 1.390; p value: 0.4990), (**C**) time spent interacting with the new food (Kruskal-Wallis statistic: 4.467; p value: 0.1071). WT – wild-type mice (n=12), *Slc5a6*^{P437Q} – mice with the *Slc5a6*^{P437Q} mutation (n=10), *Slc5a6*$^{R280W/P437Q}$ – heterozygous mice with the *Slc5a6*^{R280W} and *Slc5a6*^{P437Q} alleles (n=8). The data are presented as the interquartile range ± range, with dashes indicating the medians and dots indicating individual values. (**D**) Comparison of the anxiety behavior in the “Marble burying" test of mice with a mutation in the *Slc5a6* gene with the performance of wild-type mice (Kruskal-Wallis statistic: 0.8837; p-value: 0.6429). WT – wild-type mice (n=12), *Slc5a6*^{P437Q} – mice with the *Slc5a6*^{P437Q} mutation (n=12), *Slc5a6*$^{R280W/P437Q}$ – heterozygous mice with the *Slc5a6*^{R280W} and *Slc5a6*^{P437Q} alleles (n=8). The data are presented as interquartile range ± range, with dashes indicating medians and dots indicating individual values.

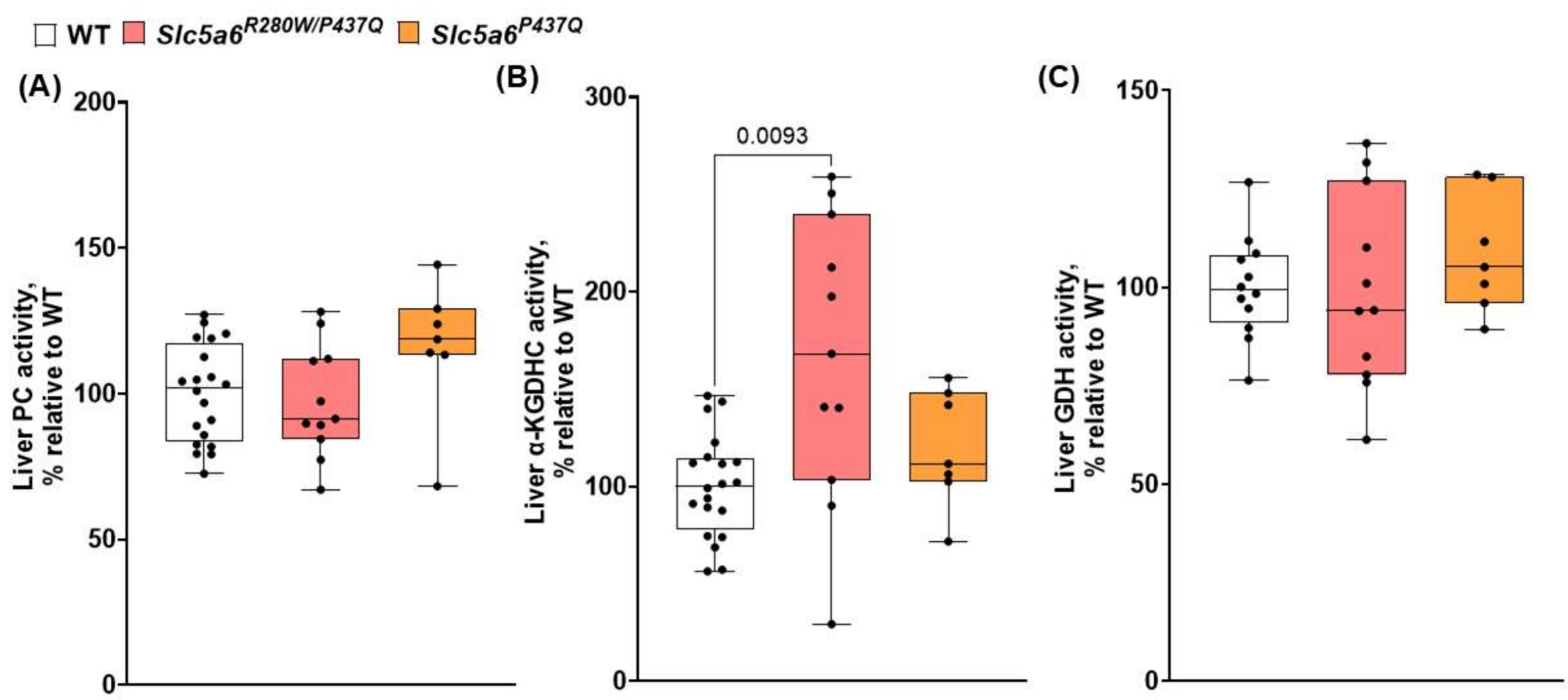

Supplementary Figure 4. (**A-C**) Comparison of activity of main biotin-dependent protein complexes. (**A**) Measurement of liver homogenate PC activity (Kruskal-Wallis statistic: 4.141; p

value: 0.1261), average PC activity of WT mice set as 100% was 2.37±0.16 µmol/min per g FW; (**B**) Measurement of liver a-KGDHC activity (Kruskal-Wallis statistic: 8.365; p value: 0.0153), average a-KGDHC activity of WT mice set as 100% was 0.74±0.07 µmol/min per g FW; (**C**) Measurement of liver glutamate dehydrogenase (GDH) activity (Kruskal-Wallis statistic: 1.369; p value: 0.5044), average GDH activity of WT mice set as 100% was 20.5±0.8 µmol/min per g FW. WT – wild-type mice (n=12), *Slc5a6*$^{R280W/P437Q}$ – heterozygous mice with the *Slc5a6*^{R280W} and *Slc5a6*^{P437Q} alleles (n=11), *Slc5a6*^{P437Q} – homozygous mice with *Slc5a6*^{P437Q} allele (n=7). The data are presented as interquartile range ± range, with dashes indicating medians and dots indicating individual values.

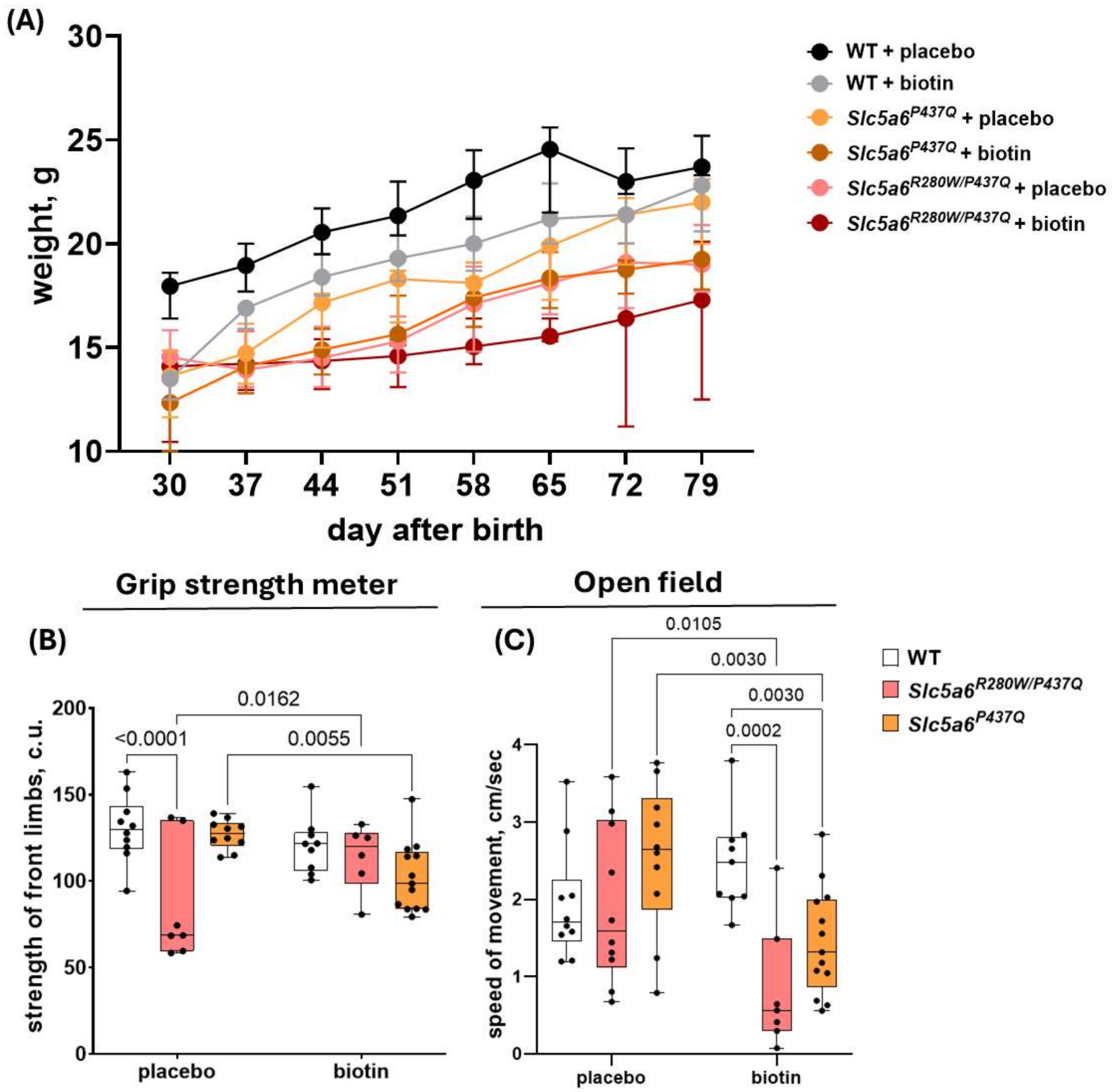

Supplementary Figure 5. (**A**) Comparison of the weight of male mice with *Slc5a6* gene mutation with the weight of wild-type mice with biotin supplementation or with a placebo. WT – wild-type mice (control n=10, biotin supplementation n=9), *Slc5a6*^{P437Q} – mice with homozygous *Slc5a6*^{P437Q}

mutation (control n=10, biotin supplementation n=18), *Slc5a6*$^{R280W/P437Q}$ – mice carrying heterozygous genotype *Slc5a6*^{R280W} and *Slc5a6*^{P437Q} (control n=18, biotin supplementation n=18). The Benjamini, Krieger, and Yekutieli correction was applied for multiple comparisons (χ^2 value = 243.0 and adjusted q-value < 0.0001). The data are presented as medians ± 95% confidence intervals. (**B**) Comparison of the strength of male mice with *Slc5a6* gene mutation with the weight of wild-type mice with biotin supplementation or with a placebo in "Grip strength" test (Interaction: p=0.0019, F (2, 49) = 7.168; Supplement factor: p=0.6985, F (1, 49) = 0.1518; Genotype factor: p=0.0041, F (2, 49) = 6.160). WT – wild-type mice (control n=10, biotin supplementation n=9), *Slc5a6*^{P437Q} – mice with homozygous *Slc5a6*^{P437Q} mutation (control n=10, biotin supplementation n=13), *Slc5a6*$^{R280W/P437Q}$ – mice carrying heterozygous genotype *Slc5a6*^{R280W} and *Slc5a6*^{P437Q} (control n=7, biotin supplementation n=6). (**C**) Comparison of the speed of male mice with *Slc5a6* gene mutation with the weight of wild-type mice with biotin supplementation or with placebo in "Open field" test (Interaction: p=0.0037, F (2, 53) = 6.229; Supplement factor: p=0.0161, F (1, 53) = 6.182; Genotype factor: p=0.0124, F (2, 53) = 4.778). WT – wild-type mice (control n=10, biotin supplementation n=9), *Slc5a6*^{P437Q} – mice with homozygous *Slc5a6*^{P437Q} mutation (control n=10, biotin supplementation n=13), *Slc5a6*$^{R280W/P437Q}$ – mice carrying heterozygous genotype *Slc5a6*^{R280W} and *Slc5a6*^{P437Q} (control n=10, biotin supplementation n=7). The data are presented as interquartile range ± range, with dashes indicating medians and dots indicating individual values.

Supplementary Table 1. Blood amino acid and acylcarnitine levels of the patient at 9 months of age. Values exceeding the reference range are highlighted in red.

**Parameter**	**Concentration, µmol/L**	**Reference range, µmol/L**
5-Oxoproline (5-Oxo Pro)	23	12-170
Alanine (Ala)	251	78-850
Arginine (Arg)	14	2-113
Aspartic acid (Asp)	91	26-500
Citrulline (Cit)	26	5-60
Cysteine (Cys)	0.6	0-14
Glutamic acid (Glu)	207	70-755

Glycine (Gly)	200	110-855
Homocysteine (Hcy)	10	6-35
Histidine (His)	20	2-90
Leucine (Leu)	133	25-300
Methionine (Met)	23	6-110
Ornithine (Orn)	116	25-400
Phenylalanine (Phe)	46	19-180
Proline (Pro)	95	30-420
Serine (Ser)	205	65-1000
Threonine (Thr)	52	10-135
Tryptophan (Trp)	9	3-30
Tyrosine (Tyr)	78	15-200
Valine (Val)	155	40-380
Free Carnitine (C0 MRM)	50	10-135
Decanoylcarnitine (C10)	0.076	0-0.4
Decenoylcarnitine (C10:1)	0.063	0-0.5
Decadienoylcarnitine (C10:2)	0.027	0-0.22

Dodecanoylcarnitine (C12)	0.039	0-0.41
Dodecenoylcarnitine (C12:1)	0.03	0-0.32
Tetradecanoylcarnitine (C14)	0.093	0-0.53
3-Hydroxytetradecanoylcarnitine (C14 OH)	0.016	0-0.22
Tetradecenoylcarnitine (C14:1)	0.1	0-0.44
Tetradecadienoylcarnitine (C14:2)	0.04	0-0.37
Hexadecanoylcarnitine (C16)	1.04	0.16-4.2
3-Hydroxyhexadecanoylcarnitine (C16 OH)	0.022	0-0.2
Hexadecenoylcarnitine (C16:1)	0.11	0-0.39
3-Hydroxyhexadecenoylcarnitine (C16:1 OH)	0.07	0-0.3
Octadecanoylcarnitine (C18)	0.55	0.1-2
3-Hydroxyoctadecanoylcarnitine (C18 OH)	0.014	0-0.17
Octadecenoylcarnitine (C18:1)	1.8	0.12-3.75
3-Hydroxyoctadecenoylcarnitine (C18:1 OH)	0.023	0-0.19
Octadecadienoylcarnitine (C18:2)	0.41	0-1.29
Acetylcarnitine (C2 MRM)	26	7-50

Propionylcarnitine (C3 MRM)	1.6	0.2-6.5
Malonylcarnitine / 3-Hydroxybutyrylcarnitine (C3DC)	0.035	0-0.34
Butyrylcarnitine / Isobutyrylcarnitine (C4)	0.14	0-1.1
Succinylcarnitine / Methylmalonylcarnitine (C4DC)	0.5	0-2
Isovalerylcarnitine / 2-Methylbutyrylcarnitine (C5)	0.2	0-0.7
3-Hydroxyisovalerylcarnitine (C5OH)	1.2	0-0.9
Tiglylcarnitine (C5:1)	0.027	0-0.17
Glutarylcarnitine (C5DC)	0.077	0-0.4
Hexanoylcarnitine (C6)	0.056	0-0.36
Hexanoylcarnitine (C6DC)	0.04	0-1
Octanoylcarnitine (C8)	0.078	0-0.4
Octenoylcarnitine (C8:1)	0.13	0-0.42

Supplementary Table 2. Urine organic acids level of the patient at 9 months of age. Values exceeding the reference range are highlighted in red.

**Parameter**	**Concentration, mM**	**Reference range, mM**
2-Hydroxyisobutyrate	15	0.00-2.00

2-Methyl-3-hydroxybutyrate	3.4	0.00-11.00
2-Methylsuccinate	5.2	0.00-3.00
2-Ketoglutarate	48	0.00-152.0
3-Hydroxybutyrate (3-OHB)	10.5	0.00-3.00
3-Hydroxyisovaleric acid (3-HIVA)	169	0.00-46.00
4-Hydroxyphenylacetate (4-HPA)	16.2	6.00-28.00
Adipic acid	5.2	0.00-12.00
Hippuric acid	1160	170.00-390.00
Glycolic acid	28	11.00-103.00
Homovanillic acid (HVA)	7.4	2.00-15.00
Lactate (Lac)	6.4	0.00-25.00
Methylmalonic acid (MMA)	1.6	0.00-2.00
Urea	18	2.00-22.00
Pyruvate (Pyr)	4.1	0.00-12.00
Suberic acid	1.1	0.00-2.00
Succinate	8.5	0.50-16.00

Vanillylmandelic acid (VMA)	1.5	1.00-15.00
Ethylmalonic acid (EMA)	1.7	0.00-7.00

Supplementary Table 3. Comparison of phenotypes in mice with mutations in the *Slc5a6* gene.

**Parameter**	***Slc5a6***^{P437Q}	***Slc5a6***$^{\mathbf{R280W/P437Q}}$
Survival	Slightly reduced	Dramatic reduction
Weight gain	Reduced	Pronounced deficit
Muscle strength	Slightly reduced	Dramatic reduction
Anxiety	Slightly increased	Reduced threat response
Associative memory	Deficit	Deficit
Hyponeophagia	Indistinguishable from wild type	Eat less

Supplementary Table 4. Blood amino acid and acylcarnitine levels of mice with mutations in the *Slc5a6* gene fed standard chow or biotin-supplemented chow, presented as mean ± SD. Group sizes were: WT + placebo, n = 5; *Slc5a6*$^{R280W/P437Q}$ + placebo, n = 4; *Slc5a6*^{P437Q} + placebo, n = 5; WT + biotin, n = 5; *Slc5a6*$^{R280W/P437Q}$ + biotin, n = 4; and *Slc5a6*^{P437Q} + biotin, n = 5. Statistical significance is indicated as follows: * $q < 0.05$, ** $q < 0.01$, *** $q < 0.001$ versus the corresponding WT group within the same supplementation condition; # $q < 0.05$, ## $q < 0.01$, ### $q < 0.001$ for placebo versus biotin within the same genotype. Full statistical results are presented in Supplementary Tables S5–S9.

Parameter	***WT + Placebo, n=5***	***Slc5a6***$^{R280W/P437Q}$ ***+ placebo, n=4***	***Slc5a6***^{P437Q} ***+ placebo, n=5***	***WT + biotin, n=5***	***Slc5a6***$^{R280W/P437Q}$ ***+ biotin, n=4***	***Slc5a6***^{P437Q} ***+ biotin, n=5***

**Amino Acids (AA)**						
**5-Oxoproline (AA 5-Oxo Pro)**	54±5#	51±2	52±6	47±4	49±9	51±2
**Alanine (AA Ala)**	396±50	214±48**	241±38**###	436±29	251±95***	492±122
**Arginine (AA Arg)**	225±28#	105±4***	154±8**	172±46	128±48	153±48
**Aspartic acid (AA Asp)**	126±13	91±10**#	94±9**	112±10	68±15***	102±24
**Citrulline (AA Cit)**	72±10	67±11	72±3###	64±7	58±3	105±19***
**Cysteine (AA Cys)**	2±0.1	4±0***	2±0	2±0	4±0***	2±0
**Glutamic acid (AA Glu)**	292±25	170±3***	272±39	273±29	172±30***	266±27
**Glycine (AA Gly)**	428±69	293±7**	334±32*	437±36	372±122	365±43
**Homocysteine (AA Hcy)**	9±0.2	7±0***	9±0*	9±0	7±0***	9±0*
**Histidine (AA His)**	30±3	14±2***	24±5**	28±4	14±3***	22±2*
**Leucine (AA Leu)**	280±32	217±33*	179±50**	262±26	235±52	161±49**

**Methionine (AA Met)**	77±17	38±7***	47±5***	77±5	43±15***	58±18*
**Ornithine (AA Orn)**	67±5	51±18	57±4	87±21	53±19	79±46
**Phenylalanine (AA Phe)**	75±10	63±10$^{\#}$	60±9	83±9	80±19	65±7
**Proline (AA Pro)**	89±17	62±6	64±6$^{\#\#}$	102±20	69±21*	101±30
**Serine (AA Ser)**	298±38	167±6***	233±22$^{*\#\#}$	279±29	177±26***	306±60
**Threonine (AA Thr)**	71±11	40±6**	46±4$^{**\#}$	75±3	42±12**	68±25
**Tryptophan (AA Trp)**	45±6	19±3**	41±9	38±15	20±9**	41±14
**Tyrosine (AA Tyr)**	98±15	48±9$^{***\#}$	88±10	106±9	72±12***	91±13*
**Valine (AA Val)**	264±26	170±27***	170±29***	260±13	180±40***	164±34***
**Acylcarnitines (AC)**						
**Free Carnitine (AC C0)**	36±2	36±3$^{\#\#}$	39±3$^{\#}$	37±4	27±6**	33±6
**Acetylcarnitine (AC C2)**	42±4	55±5$^{**\#}$	48±7	42±2	45±8	45±4

**Propionylcarnitine (AC C3)**	1.07±0.1	0.77±0.1**	0.88±0.2*###	1,04±0.1	0.7±0.1***	0.59±0.1***
**Malonylcarnitine (AC C3DC)**	0.24±0.02	0.23±0.03#	0.28±0.04###	0.22±0.02	0.29±0.05**	0.38±0.03***
**Butyrylcarnitine / Isobutyrylcarnitine (AC C4)**	1±0.1###	0.4±0.1	1±0.15	2±1	0.3±0.05***	1±0.2**
**Succinylcarnitine / Methylmalonylcarnitine (AC C4DC)**	0.5±0.05	1±0.1**	1±0.1	0.4±0.04	1±0.2***	0.5±0.1
**3-Hydroxybutyrylcarnitine (AC C4OH)**	1±0.1	0.5±0.1	1±0.1**###	1±0.03	0.4±0.1	2±0.3***
**Isovalerylcarnitine / 2-Methylbutyrylcarnitine (AC C5)**	0.1±0.004	0.15±0.01#	0.1±0.02	0.2±0.03	0.1±0.01*	0.1±0.04
**Tiglylcarnitine (AC C5:1)**	0.02±0.004	0.02±0.003	0.02±0.004	0.02±0.002	0.02±0.003	0.02±0.001
**Glutarylcarnitine (AC C5DC)**	0.1±0.02	0.1±0.02#	0.1±0.03###	0.1±0.01	0.16±0.04*	0.2±0.05***

**3-Hydroxyisovalerylcarnitine (AC C5OH)**	0.2±0.02	0.6±0.4**	0.2±0.03	0.2±0.02	0.4±0.1	0.2±0.03
**Hexanoylcarnitine (AC C6)**	0.1±0.01	0.1±0.03	0.1±0.02#	0.1±0.05	0.1±0.02**	0.2±0.02
**Hexanedioylcarnitine (AC C6DC)**	0.03±0.01	0.1±0.02###	0.04±0.01	0.04±0.01	0.1±0.02***	0.04±0.004
**3-Hydroxyhexanoylcarnitine (AC C6OH)**	0.1±0.01	0.1±0.01	0.1±0.01#	0.1±0.02	0.1±0.02	0.1±0.01
**Octanoylcarnitine (AC C8)**	0.1±0.01	0.1±0.02##	0.1±0.01###	0.05±0.01	0.1±0.02***	0.2±0.05***
**Octenoylcarnitine (AC C8:1)**	0.02±0.004	0.02±0.004##	0.015±0.002###	0.02±0.01	0.04±0.01***	0.03±0.01**
**Octanedioylcarnitine (AC C8DC)**	0.04±0.01	0.04±0.01###	0.04±0.004	0.04±0.01	0.1±0.02***	0.05±0.01
**Decanoylcarnitine (AC C10)**	0.1±0.01	0.1±0.01###	0.1±0.01###	0.05±0.01	0.1±0.02***	0.1±0.03***
**Decenoylcarnitine (AC C10:1)**	0.05±0.01	0.1±0.01###	0.04±0.01	0.04±0.01	0.1±0.04***	0.1±0.01*

**Decadienoylcarnitine (AC C10:2)**	0.02±0.003	0.02±0.01##	0.01±0.002	0.01±0.001	0.03±0.01***	0.02±0.002
**Decanedioylcarnitine (AC C10DC)**	0.1±0.01	0.1±0.01###	0.1±0.02###	0.1±0.01	0.2±0.02***	0.15±0.02***
**Dodecanoylcarnitine (AC C12)**	0.1±0.02	0.1±0.02###	0.1±0.03#	0.1±0.01	0.3±0.05***	0.15±0.03**
**Dodecenoylcarnitine (AC C12:1)**	0.04±0.01	0.04±0.01###	0.04±0.02	0.04±0.01	0.3±0.1***	0.1±0.04*
**Dodecanedioylcarnitine (AC C12DC)**	0.1±0.005	0.05±0.01###	0.1±0.01	0.05±0.01	0.2±0.1***	0.1±0.02*
**Tetradecanoylcarnitine (AC C14)**	0.3±0.03#	0.3±0.1###	0.3±0.1	0.2±0.03	0.5±0.04***	0.3±0.05**
**Tetradecenoylcarnitine (AC C14:1)**	0.1±0.03	0.1±0.04###	0.1±0.03	0.1±0.02	1±0.2***	0.2±0.05
**Tetradecadienoylcarnitine (AC C14:2)**	0.03±0.004	0.04±0.01###	0.03±0.01	0.02±0.005	0.2±0.1***	0.03±0.01
**3-Hydroxytetradecanoylcarnitine (AC C14OH)**	0.05±0.01#	0.05±0.01	0.03±0.01*	0.03±0.01	0.1±0.003***	0.04±0.01

**Hexadecanoyl carnitine (AC C16)**	1.54±0.1	1.85±0.2	1.7±0.2$^{\#\#\#}$	1.3±0.1	2.0±0.2***	2.47±0.3***
**Hexadecenoyl carnitine (AC C16:1)**	0.3±0.05	0.2±0.1$^{\#\#\#}$	0.3±0.1	0.2±0.03	1±0.3***	0.3±0.1*
**3-Hydroxyhexadecenoylcarnitine (AC C16:1OH)**	0.081±0.02	0.069±0.01$^{\#\#\#}$	0.062±0.02$^{\#}$	0.062±0.02	0.13±0.02***	0.084±0.01*
**3-Hydroxyhexadecanoylcarnitine (AC C16OH)**	0.2±0.04#	0.15±0.03	0.1±0.03**	0.1±0.01	0.2±0.04*	0.1±0.03
**Octadecanoyl carnitine (AC C18)**	0.331±0.01	0.325±0.01	0.334±0.04$^{\#\#\#}$	0.297±0.04	0.342±0.03*	0.784±0.1***
**Octadecenoyl carnitine (AC C18:1)**	0.683±0.04	0.733±0.1$^{\#\#\#}$	0.791±0.1$^{\#\#\#}$	0.642±0.05	1.23±0.1***	1.214±0.1***
**3-Hydroxyoctadecenoylcarnitine (AC C18:1OH)**	0.1±0.02	0.1±0.02$^{\#\#}$	0.04±0.01*	0.05±0.01	0.1±0.03***	0.1±0.01
**Octadecadienoylcarnitine (AC C18:2)**	0.2±0.02	0.3±0.05$^{\#\#\#}$	0.2±0.05	0.1±0.01	1±0.3***	0.2±0.05

**3-Hydroxyoctadecanoylcarnitine (AC C18OH)**	0.034±0.01	0.045±0.01$^{###}$	0.035±0.01$^{###}$	0.025±0.004	0.075±0.01***	0.071±0.02***
**Organic Acids (OA)**						
**3-O-methyldopa (OA 3-OMD)**	0.5±0.1	0.3±0.04**	0.5±0.1	0.4±0.1	0.4±0.1	0.5±0.1
**Succinylacetone (OA SuAc)**	0.91±0.04$^{##}$	0.98±0.05$^{*#}$	0.86±0.03$^{*#}$	0.82±0.1	0.90±0.04**	0.78±0.04
**Very Long-Chain Fatty Acids**						
**Eicosanoic acid / Arachidic acid (C20)**	0.02±0.004	0.01±0.002$^{###}$	0.02±0.005$^{###}$	0.02±0.01	0.2±0.1***	0.2±0.1***
**Docosanoic acid / Behenic acid (C22)**	0.02±0.002	0.03±0.01$^{###}$	0.02±0.01	0.02±0.01	0.3±0.2***	0.1±0.04*
**Tetracosanoic acid / Lignoceric acid (C24)**	0.01±0.002	0.02±0.003$^{###}$	0.02±0.004	0.01±0.004	0.2±0.1***	0.03±0.01
**Hexacosanoic acid / Cerotic acid (C26)**	0.009±0.002#	0.007±0.001$^{#}$	0.009±0.003	0.006±0.0004	0.012±0.004**	0.01±0.003**
**Ratios**						

**(C14 + C14:1 + C16:1) / C0 Ratio**	0.02±0.002	0.02±0.005$^{\#\#\#}$	0.02±0.003	0.01±0.003	0.1±0.02***	0.03±0.01*
**C0 / (C16 + C18) Ratio**	19±1$^{\#\#}$	16±1$^{\#\#\#}$	19±2$^{\#\#\#}$	23±3	12±1***	10±2***
**C14:1 / C12:1 Ratio**	4±1	3±1	5±2$^{\#}$	3±1	3±0.3	2±0.4
**C14:1 / C16 Ratio**	0.1±0.02	0.1±0.02$^{\#\#\#}$	0.1±0.02	0.1±0.01	0.4±0.1***	0.1±0.02
**C14:1 / C2 Ratio**	0.003±0.0004	0.003±0.001$^{\#\#\#}$	0.003±0.001	0.002±0.001	0.02±0.005***	0.004±0.001
**C3 / C0 Ratio**	0.03±0.003	0.02±0.001**	0.02±0.004**	0.03±0.01	0.03±0.004	0.02±0.003***
**C3 / C2 Ratio**	0.03±0.003	0.01±0.003***	0.02±0.005$^{**\#\#}$	0.03±0.004	0.02±0.003***	0.01±0.002***
**C3 / Methionine Ratio**	0.01±0.003	0.02±0.003	0.02±0.004$^{\#}$	0.01±0.002	0.02±0.01	0.01±0.004
**HADHA Ratio**	0.01±0.002	0.01±0.002$^{\#\#\#}$	0.004±0.001*#	0.01±0.001	0.01±0.004***	0.01±0.002
**MCAD Ratio**	0.1±0.005	0.1±0.01$^{\#}$	0.1±0.01	0.1±0.02	0.1±0.01	0.1±0.02
**Phenylalanine / Tyrosine (Phe/Tyr) Ratio**	1±0.1	1±0.1$^{***\#}$	1±0.1	1±0.1	1±0.2***	1±0.1

**Methionine / Phenylalanine (Met/Phe) Ratio**	1 ± 0.1	$1\pm0.1^{***}$	$1\pm0.05^{**}$	1 ± 0.1	$0.5\pm0.1^{***}$	1 ± 0.2
**(C16 + C18:1) / C2 Ratio**	0.05 ± 0.003	0.05 ± 0.01###	$0.05\pm0.004^{\#\#\#}$	0.05 ± 0.004	$0.1\pm0.01^{***}$	$0.1\pm0.005^{***}$

Supplementary Table 5. Statistical data for amino acid blood levels of mice with mutations in the *Slc5a6* gene fed standard chow or biotin-supplemented chow. For each parameter, raw two-way ANOVA p-values and panel-wide BKY-adjusted q-values are reported for the genotype × supplementation interaction, supplementation, and genotype effects. Pairwise comparisons are reported as within-parameter BKY-adjusted q-values. Panel-wide correction was applied separately to each omnibus effect across all 76 analyzed metabolic parameters. Pairwise q-values are color-coded as follows: green — q < 0.05, orange — q < 0.01, red — q < 0.001.

		q values								
		**placebo**			**biotin**			**WT**	***Slc5a6**$^{R280W/P437Q}$*	***Slc5a6**^{P437Q}*
**Parameter**		WT vs. *Slc5a6*$^{R280W/P437Q}$	WT vs. *Slc5a6*^{P437Q}	*Slc5a6*$^{R280W/P437Q}$ vs. *Slc5a6*^{P437Q}	WT vs. *Slc5a6*$^{R280W/P437Q}$	WT vs. *Slc5a6*^{P437Q}	*Slc5a6*$^{R280W/P437Q}$ vs. *Slc5a6*^{P437Q}	placebo vs. biotin	placebo vs. biotin	placebo vs. biotin
**AA 5-Oxo Pro**	Interaction: P=0.4789, panel q=0.2871, $F(2, 22) = 0.7616$ Supplement factor: P=0.0692, panel	0.8181	0.8181	0.8181	0.7262	0.7262	0.7262	0.0465	0.4715	0.6906

	q=0.0495, F (1, 22) = 3.650 Genotype factor: P=0.7920, panel q=0.0776, F (2, 22) = 0.2357									
**AA Ala**	Interaction: P=0.0043, panel q=0.0051, F (2, 22) = 7.040 Supplement factor: P=0.0006, panel q=0.0007, F (1, 22) = 16.07 Genotype factor: P<0.0001, panel q<0.0001, F (2, 22) = 15.25	0.001	0.0013	0.2031	0.0005	0.0821	<0.0001	0.4086	0.496	<0.0001
**AA Arg**	Interaction: P=0.0835, panel q=0.0705, F (2, 22) = 2.786 Supplement factor: P=0.4538, panel q=0.2346, F (1, 22) = 0.5815 Genotype factor: P=0.0003, panel	<0.0001	0.0023	0.0188	0.2555	0.4386	0.4386	0.0282	0.3885	>0.9999

	q<0.0001, F (2, 22) = 11.94									
**AA Asp**	Interaction: P=0.0866, panel q=0.0716, F (2, 22) = 2.740 Supplement factor: P=0.0883, panel q=0.0593, F (1, 22) = 3.182 Genotype factor: P<0.0001, panel q<0.0001, F (2, 22) = 16.67	0.0011	0.0011	0.2658	0.0002	0.0971	0.0012	0.1426	0.0373	0.4326
**AA Cit**	Interaction: P=0.0003, panel q=0.0004, F (2, 22) = 12.38 Supplement factor: P=0.2222, panel q=0.1347, F (1, 22) = 1.578 Genotype factor: P<0.0001, panel q<0.0001, F (2, 22) = 15.79	0.85	>0.9999	0.85	0.1471	<0.0001	<0.0001	0.2505	0.2275	<0.0001
**AA Cys**	Interaction: P=0.1966, panel q=0.1469, F (2, 22) = 1.753	<0.0001	0.3368	<0.0001	<0.0001	0.1176	<0.0001	0.7718	0.0762	0.5233

	Supplement factor: P=0.3388, panel q=0.1836, F (1, 22) = 0.9563 Genotype factor: P<0.0001, panel q<0.0001, F (2, 22) = 1188									
**AA Glu**	Interaction: P=0.7430, panel q=0.4009, F (2, 22) = 0.301 Supplement factor: P=0.4798, panel q=0.2442, F (1, 22) = 0.5168 Genotype factor: P<0.0001, panel q<0.0001, F (2, 22) = 39.03	<0.0001	0.1033	<0.0001	<0.0001	0.2504	<0.0001	0.3221	0.9808	0.7714
**AA Gly**	Interaction: P=0.4810, panel q=0.2871, F (2, 22) = 0.7569 Supplement factor: P=0.0977, panel q=0.0644, F (1, 22) = 2.991 Genotype	0.0032	0.0116	0.1128	0.19	0.19	0.9114	0.852	0.0822	0.4467

	factor: P=0.0034, panel q=0.0004, F (2, 22) = 7.431									
**AA Hcy**	Interaction: P=0.3619, panel q=0.2268, F (2, 22) = 1.065 Supplement factor: P=0.7904, panel q=0.366, F (1, 22) = 0.07237 Genotype factor: P<0.0001, panel q<0.0001, F (2, 22) = 155.2	<0.0001	0.0236	<0.0001	<0.0001	0.0424	<0.0001	0.5542	0.3479	0.368
**AA His**	Interaction: P=0.8366, panel q=0.4414, F (2, 22) = 0.1798 Supplement factor: P=0.4309, panel q=0.2262, F (1, 22) = 0.6438 Genotype factor: P<0.0001, panel q<0.0001, F (2, 22) = 45.23	<0.0001	0.0085	0.0004	<0.0001	0.0117	0.002	0.4461	>0.9999	0.5334

**AA Leu**	Interaction: P=0.6070, panel q=0.352, F (2, 22) = 0.5107 Supplement factor: P=0.7027, panel q=0.3325, F (1, 22) = 0.1495 Genotype factor: P<0.0001, panel q<0.0001, F (2, 22) = 14.73	0.0376	0.0019	0.1265	0.12	0.001	0.0079	0.5258	0.5897	0.5344
**AA Met**	Interaction: P=0.6490, panel q=0.3644, F (2, 22) = 0.4409 Supplement factor: P=0.2473, panel q=0.1458, F (1, 22) = 1.412 Genotype factor: P<0.0001, panel q<0.0001, F (2, 22) = 20.59	0.0001	0.0006	0.1001	0.0005	0.0123	0.028	0.9706	0.6081	0.179
**AA Orn**	Interaction: P=0.6566, panel q=0.3644, F (2, 22) = 0.4289 Supplement	0.7499	0.7499	0.7499	0.1571	0.6362	0.2016	0.2092	0.9249	0.1681

	factor: P=0.1164, panel q=0.0752, F (1, 22) = 2.672 Genotype factor: P=0.1172, panel q=0.0123, F (2, 22) = 2.368									
**AA Phe**	Interaction: P=0.4877, panel q=0.2871, F (2, 22) = 0.7420 Supplement factor: P=0.0235, panel q=0.0188, F (1, 22) = 5.930 Genotype factor: P=0.0104, panel q=0.0012, F (2, 22) = 5.662	0.147	0.1008	0.7241	0.7851	0.0691	0.0915	0.3361	0.0354	0.43
**AA Pro**	Interaction: P=0.2410, panel q=0.1587, F (2, 22) = 1.519 Supplement factor: P=0.0175, panel q=0.0147, F (1, 22) = 6.595 Genotype factor: P=0.0116,	0.09	0.09	0.8734	0.011	0.3235	0.011	0.3097	0.6444	0.0072

	panel q=0.0013, F (2, 22) = 5.495									
**AA Ser**	Interaction: P=0.0259, panel q=0.0251, F (2, 22) = 4.335 Supplement factor: P=0.1244, panel q=0.0789, F (1, 22) = 2.552 Genotype factor: P<0.0001, panel q<0.0001, F (2, 22) = 26.66	<0.0001	0.0112	0.0112	0.0002	0.0826	<0.0001	0.435	0.7202	0.0038
**AA Thr**	Interaction: P=0.2271, panel q=0.1548, F (2, 22) = 1.587 Supplement factor: P=0.0738, panel q=0.0517, F (1, 22) = 3.524 Genotype factor: P=0.0001, panel q<0.0001, F (2, 22) = 13.83	0.0017	0.0033	0.1629	0.001	0.1285	0.0038	0.6468	0.8568	0.0167
**AA Trp**	Interaction: P=0.6938, panel q=0.3796, F (2, 22) =	0.0014	0.1911	0.0028	0.0097	0.2582	0.0094	0.3088	0.9661	0.9491

	0.3717 Supplement factor: P=0.5539, panel q=0.2778, F (1, 22) = 0.3613 Genotype factor: P=0.0003, panel q<0.0001, F (2, 22) = 11.91									
**AA Tyr**	Interaction: P=0.2133, panel q=0.1483, F (2, 22) = 1.659 Supplement factor: P=0.0140, panel q=0.0121, F (1, 22) = 7.124 Genotype factor: P<0.0001, panel q<0.0001, F (2, 22) = 29.65	<0.0001	0.0641	<0.0001	0.0002	0.0206	0.0101	0.2829	0.0102	0.6419
**AA Val**	Interaction: P=0.8407, panel q=0.4414, F (2, 22) = 0.1748 Supplement factor: P=0.9877, panel q=0.4367, F (1, 22) =	<0.0001	<0.0001	0.3498	0.0002	<0.0001	0.1544	0.8759	0.6871	0.7867

	0.0002415 Genotype factor: P<0.0001, panel q<0.0001, F (2, 22) = 32.22									

Supplementary Table 6. Statistical data for short-chain acylcarnitines and related metabolites in blood of mice with mutations in the *Slc5a6* gene fed standard chow or biotin-supplemented chow. For each parameter, raw two-way ANOVA p-values and panel-wide BKY-adjusted q-values are reported for the genotype × supplementation interaction, supplementation, and genotype effects. Pairwise comparisons are reported as within-parameter BKY-adjusted q-values. Panel-wide correction was applied separately to each omnibus effect across all 76 analyzed metabolic parameters. Pairwise q-values are color-coded as follows: green — q < 0.05, orange — q < 0.01, red — q < 0.001.

		q values								
		**placebo**			**biotin**			**WT**	***Slc5a6$^{R280W/P437Q}$***	***Slc5a6^{P437Q}***
**Parameter**		WT vs. *Slc5a6$^{R280W/P437Q}$*	WT vs. *Slc5a6^{P437Q}*	*Slc5a6$^{R280W/P437Q}$* vs. *Slc5a6^{P437Q}*	WT vs. *Slc5a6$^{R280W/P437Q}$*	WT vs. *Slc5a6^{P437Q}*	*Slc5a6$^{R280W/P437Q}$* vs. *Slc5a6^{P437Q}*	placebo vs. biotin	placebo vs. biotin	placebo vs. biotin
**AC C0**	Interaction: P=0.0497, panel q=0.0449, F (2, 22) = 3.450 Supplement factor: P=0.0101, panel	>0.9999	0.3833	0.3833	0.0042	0.1373	0.0369	0.6929	0.0075	0.0402

	q=0.0092, F (1, 22) = 7.915 Genotype factor: P=0.0374, panel q=0.004, F (2, 22) = 3.830									
**AC C2**	Interaction: P=0.2037, panel q=0.1469, F (2, 22) = 1.712 Supplement factor: P=0.0260, panel q=0.0201, F (1, 22) = 5.700 Genotype factor: P=0.0139, panel q=0.0016, F (2, 22) = 5.224	0.0037	0.0733	0.0661	0.5795	0.5795	0.9519	0.8302	0.0144	0.3242
**AC C3**	Interaction: P=0.0469, panel q=0.0433, F (2, 22) = 3.529 Supplement factor: P=0.0096, panel q=0.009, F (1, 22) = 8.041	0.0015	0.0133	0.0642	0.0002	<0.0001	0.0563	0.7598	0.4621	0.0009

	Genotype factor: P<0.0001, panel q<0.0001, F (2, 22) = 22.04									
**AC C3DC**	Interaction: P=0.0015, panel q=0.0019, F (2, 22) = 8.877 Supplement factor: P=0.0016, panel q=0.0018, F (1, 22) = 12.97 Genotype factor: P<0.0001, panel q<0.0001, F (2, 22) = 23.31	0.4457	0.152	0.0714	0.0075	<0.0001	0.0006	0.288	0.0178	0.0001
**AC C4**	Interaction: P=0.0147, panel q=0.015, F (2, 22) = 5.144 Supplement factor: P=0.0067, panel q=0.0067, F (1, 22) = 8.947 Genotype factor: P<0.0001, panel	0.3317	0.4266	0.4266	<0.0001	0.0045	0.0062	0.0004	0.6394	0.0974

	q<0.0001, F (2, 22) = 15.69									
**AC C4DC**	Interaction: P=0.4716, panel q=0.2871, F (2, 22) = 0.7779 Supplement factor: P=0.5728, panel q=0.283, F (1, 22) = 0.3278 Genotype factor: P<0.0001, panel q<0.0001, F (2, 22) = 21.28	0.0022	0.2742	0.0022	<0.0001	0.111	0.0002	0.249	0.5934	0.6753
**AC C4OH**	Interaction: P<0.0001, panel q<0.0001, F (2, 22) = 29.01 Supplement factor: P=0.0003, panel q=0.0003, F (1, 22) = 18.98 Genotype factor: P<0.0001, panel q<0.0001,	0.1367	0.0034	0.0013	0.1703	<0.0001	<0.0001	0.5589	0.7217	<0.0001

	F (2, 22) = 86.37									
**AC C5**	Interaction: P=0.0152, panel q=0.0151, F (2, 22) = 5.096 Supplement factor: P=0.7952, panel q=0.366, F (1, 22) = 0.06903 Genotype factor: P=0.5439, panel q=0.0548, F (2, 22) = 0.6261	0.3214	0.8887	0.3214	0.0182	0.3471	0.0381	0.1581	0.0156	0.3481
**AC C5:1**	Interaction: P=0.9414, panel q=0.4812, F (2, 22) = 0.06055 Supplement factor: P=0.8885, panel q=0.398, F (1, 22) = 0.02013 Genotype factor: P=0.0371, panel q=0.004, F (2, 22) = 3.839	0.3002	0.1353	0.5319	0.5009	0.3023	0.5009	0.8817	0.8622	0.8817

**AC C5DC**	Interaction: P=0.0030, panel q=0.0037, F (2, 22) = 7.631 Supplement factor: P=0.0004, panel q=0.0005, F (1, 22) = 17.11 Genotype factor: P=0.0032, panel q=0.0004, F (2, 22) = 7.546	0.9017	>0.9999	0.9017	0.0209	<0.0001	0.0232	0.6586	0.015	<0.0001
**AC C5OH**	Interaction: P=0.3318, panel q=0.2113, F (2, 22) = 1.160 Supplement factor: P=0.2244, panel q=0.1347, F (1, 22) = 1.563 Genotype factor: P=0.0026, panel q=0.0003, F (2, 22) = 7.866	0.0015	0.3296	0.0015	0.2855	0.6465	0.3316	0.7968	0.0819	0.9473
**AC C6**	Interaction: P=0.0978,	0.4105	0.8807	0.4105	0.0035	0.0562	0.0003	0.2854	0.612	0.0113

	panel q=0.0792, F (2, 22) = 2.589 Supplement factor: P=0.0817, panel q=0.056, F (1, 22) = 3.329 Genotype factor: P=0.0022, panel q=0.0003, F (2, 22) = 8.218									
**AC C6DC**	Interaction: P<0.0001, panel q<0.0001, F (2, 22) = 16.79 Supplement factor: P=0.0001, panel q=0.0002, F (1, 22) = 21.23 Genotype factor: P<0.0001, panel q<0.0001, F (2, 22) = 44.38	0.0641	0.9046	0.0641	<0.0001	0.2417	<0.0001	0.9666	<0.0001	0.7836
**AC C6OH**	Interaction: P=0.1043, panel q=0.0827, F (2, 22) = 2.509	0.5781	0.1466	0.2847	0.4944	0.4944	>0.9999	0.3304	0.5601	0.0464

	Supplement factor: P=0.3254, panel q=0.1836, F (1, 22) = 1.012 Genotype factor: P=0.5841, panel q=0.058, F (2, 22) = 0.5510									
**AC C8**	Interaction: P<0.0001, panel q<0.0001, F (2, 22) = 19.43 Supplement factor: P<0.0001, panel q<0.0001, F (1, 22) = 38.27 Genotype factor: P<0.0001, panel q<0.0001, F (2, 22) = 18.85	0.9743	0.9743	0.9743	0.0002	<0.0001	0.0017	0.4791	0.0024	<0.0001
**AC C8:1**	Interaction: P=0.0090, panel q=0.01, F (2, 22) = 5.872 Supplement factor: P<0.0001,	0.7127	0.525	0.525	0.0005	0.001	0.1481	0.9276	0.0015	0.0001

	panel q=0.0001, F (1, 22) = 24.25 Genotype factor: P=0.0140, panel q=0.0016, F (2, 22) = 5.214									
**AC C8DC**	Interaction: P<0.0001, panel q<0.0001, F (2, 22) = 43.56 Supplement factor: P<0.0001, panel q<0.0001, F (1, 22) = 69.70 Genotype factor: P<0.0001, panel q<0.0001, F (2, 22) = 59.10	0.5814	0.9631	0.5814	<0.0001	0.1501	<0.0001	0.6858	<0.0001	0.1948
**AC C10**	Interaction: P<0.0001, panel q<0.0001, F (2, 22) = 18.86 Supplement factor: P<0.0001, panel q<0.0001, F (1, 22) = 45.91	0.8642	0.8642	0.8642	<0.0001	<0.0001	0.1239	0.3741	<0.0001	<0.0001

	Genotype factor: P<0.0001, panel q<0.0001, F (2, 22) = 24.81									
**AC C10:1**	Interaction: P<0.0001, panel q<0.0001, F (2, 22) = 20.48 Supplement factor: P=0.0002, panel q=0.0002, F (1, 22) = 20.74 Genotype factor: P<0.0001, panel q<0.0001, F (2, 22) = 34.71	0.4355	0.7622	0.4355	<0.0001	0.027	<0.0001	0.2953	<0.0001	0.2953
**AC C10:2**	Interaction: P=0.0348, panel q=0.033, F (2, 22) = 3.927 Supplement factor: P=0.0509, panel q=0.0372, F (1, 22) = 4.265 Genotype factor: P=0.0032,	0.5971	0.5971	0.5971	0.0002	0.0747	0.0016	0.4535	0.0063	0.2881

	panel q=0.0004, F (2, 22) = 7.566									
**AC C10DC**	Interaction: P<0.0001, panel q<0.0001, F (2, 22) = 18.34 Supplement factor: P<0.0001, panel q<0.0001, F (1, 22) = 42.09 Genotype factor: P<0.0001, panel q<0.0001, F (2, 22) = 38.00	0.135	0.8785	0.135	<0.0001	<0.0001	<0.0001	0.3761	<0.0001	0.0001

Supplementary Table 7. Statistical data for long-chain acylcarnitines and related metabolites in blood of mice with mutations in the *Slc5a6* gene fed standard chow or biotin-supplemented chow. For each parameter, raw two-way ANOVA p-values and panel-wide BKY-adjusted q-values are reported for the genotype × supplementation interaction, supplementation, and genotype effects. Pairwise comparisons are reported as within-parameter BKY-adjusted q-values. Panel-wide correction was applied separately to each omnibus effect across all 76 analyzed metabolic parameters. Pairwise q-values are color-coded as follows: green — $q < 0.05$, orange — $q < 0.01$, red — $q < 0.001$.

		q values								
		**placebo**			**biotin**			**WT**	***Slc5a6***$^{R280W/P437Q}$	***Slc5a6***^{P437Q}

**Parameter**		WT vs. *Slc5a6*$^{R280W/P437Q}$	WT vs. *Slc5a6*^{P437Q}	*Slc5a6*$^{R280W/P437Q}$ vs. *Slc5a6*^{P437Q}	WT vs. *Slc5a6*$^{R280W/P437Q}$	WT vs. *Slc5a6*^{P437Q}	*Slc5a6*$^{R280W/P437Q}$ vs. *Slc5a6*^{P437Q}	placebo vs. biotin	placebo vs. biotin	placebo vs. biotin
**AC C12**	Interaction: P<0.0001, panel q<0.0001, F (2, 22) = 17.25 Supplement factor: P<0.0001, panel q=0.0001, F (1, 22) = 24.83 Genotype factor: P<0.0001, panel q<0.0001, F (2, 22) = 30.39	0.5711	0.696	0.5711	<0.0001	0.0011	<0.0001	0.3205	<0.0001	0.033
**AC C12:1**	Interaction: P<0.0001, panel q<0.0001, F (2, 22) = 25.07 Supplement factor: P<0.0001, panel q<0.0001, F (1, 22) = 40.40	>0.9999	>0.9999	>0.9999	<0.0001	0.0427	<0.0001	>0.9999	<0.0001	0.1523

	Genotype factor: P<0.0001, panel q<0.0001, F (2, 22) = 25.72									
**AC C12DC**	Interaction: P<0.0001, panel q<0.0001, F (2, 22) = 18.75 Supplement factor: P<0.0001, panel q<0.0001, F (1, 22) = 30.19 Genotype factor: P<0.0001, panel q<0.0001, F (2, 22) = 17.08	>0.9999	>0.9999	>0.9999	<0.0001	0.0259	<0.0001	0.6395	<0.0001	0.0678
**AC C14**	Interaction: P<0.0001, panel q<0.0001, F (2, 22) = 19.99 Supplement factor: P=0.0005, panel q=0.0007, F (1, 22) = 16.33 Genotype factor:	0.7172	0.7172	0.7172	<0.0001	0.0012	<0.0001	0.0465	<0.0001	0.0531

	P<0.0001, panel q<0.0001, F (2, 22) = 24.41									
**AC C14:1**	Interaction: P<0.0001, panel q<0.0001, F (2, 22) = 41.98 Supplement factor: P<0.0001, panel q<0.0001, F (1, 22) = 44.34 Genotype factor: P<0.0001, panel q<0.0001, F (2, 22) = 40.65	>0.9999	>0.9999	>0.9999	<0.0001	0.0674	<0.0001	0.536	<0.0001	0.5606
**AC C14:2**	Interaction: P<0.0001, panel q<0.0001, F (2, 22) = 21.85 Supplement factor: P=0.0002, panel q=0.0003, F (1, 22) = 20.17 Genotype factor: P<0.0001,	0.9327	0.9327	0.9327	<0.0001	0.1822	<0.0001	0.7358	<0.0001	>0.9999

	panel q<0.0001, F (2, 22) = 25.16									
**AC C14OH**	Interaction: P=0.0129, panel q=0.0136, F (2, 22) = 5.334 Supplement factor: P=0.8127, panel q=0.369, F (1, 22) = 0.05753 Genotype factor: P=0.0002, panel q<0.0001, F (2, 22) = 13.06	0.52	0.0347	0.011	<0.0001	0.3077	0.0005	0.0198	0.0661	0.4418
**AC C16**	Interaction: P<0.0001, panel q<0.0001, F (2, 22) = 16.59 Supplement factor: P=0.0064, panel q=0.0065, F (1, 22) = 9.073 Genotype factor: P<0.0001, panel q<0.0001,	0.092	0.2879	0.2879	<0.0001	<0.0001	0.0017	0.0727	0.3221	<0.0001

	F (2, 22) = 30.45									
**AC C16:1**	Interaction: P<0.0001, panel q<0.0001, F (2, 22) = 27.31 Supplement factor: P<0.0001, panel q<0.0001, F (1, 22) = 31.55 Genotype factor: P<0.0001, panel q<0.0001, F (2, 22) = 23.82	0.9071	0.9071	0.9071	<0.0001	0.0329	<0.0001	0.4486	<0.0001	0.2723
**AC C16:1OH**	Interaction: P=0.0001, panel q=0.0002, F (2, 22) = 14.11 Supplement factor: P=0.0017, panel q=0.0018, F (1, 22) = 12.76 Genotype factor: P=0.0022, panel q=0.0003,	0.4176	0.2254	0.5543	<0.0001	0.0382	0.0005	0.0781	<0.0001	0.0367

	F (2, 22) = 8.188									
**AC C16OH**	Interaction: P=0.1282, panel q=0.0996, F (2, 22) = 2.258 Supplement factor: P=0.3044, panel q=0.1764, F (1, 22) = 1.106 Genotype factor: P=0.0003, panel q<0.0001, F (2, 22) = 12.21	0.2464	0.0019	0.0039	0.0114	0.0653	0.0011	0.0286	0.5879	0.8924
**AC C18**	Interaction: P<0.0001, panel q<0.0001, F (2, 22) = 129.1 Supplement factor: P<0.0001, panel q<0.0001, F (1, 22) = 108.0 Genotype factor: P<0.0001, panel q<0.0001,	0.9571	0.9571	0.9571	0.028	<0.0001	<0.0001	0.1636	0.5437	<0.0001

	F (2, 22) = 134.5									
**AC C18:1**	Interaction: P<0.0001, panel q<0.0001, F (2, 22) = 22.37 Supplement factor: P<0.0001, panel q<0.0001, F (1, 22) = 65.72 Genotype factor: P<0.0001, panel q<0.0001, F (2, 22) = 39.16	0.4622	0.2684	0.4622	<0.0001	<0.0001	0.2816	0.5203	<0.0001	<0.0001
**AC C18:1OH**	Interaction: P=0.0046, panel q=0.0052, F (2, 22) = 6.949 Supplement factor: P=0.0219, panel q=0.0179, F (1, 22) = 6.088 Genotype factor: P=0.0002, panel q<0.0001,	0.3399	0.0107	0.0107	<0.0001	0.1726	<0.0001	0.1686	0.0016	0.0803

	F (2, 22) = 13.10									
**AC C18:2**	Interaction: P=0.0003, panel q=0.0004, F (2, 22) = 12.22 Supplement factor: P=0.0050, panel q=0.0052, F (1, 22) = 9.742 Genotype factor: P<0.0001, panel q<0.0001, F (2, 22) = 24.62	0.4339	0.7881	0.4339	<0.0001	0.1334	<0.0001	0.5939	<0.0001	>0.9999
**AC C18OH**	Interaction: P=0.0002, panel q=0.0003, F (2, 22) = 12.81 Supplement factor: P=0.0001, panel q=0.0002, F (1, 22) = 21.95 Genotype factor: P<0.0001, panel q<0.0001,	0.2866	>0.9999	0.2866	<0.0001	<0.0001	0.2141	0.2073	0.0007	<0.0001

	F (2, 22) = 19.59									

Supplementary Table 8. Statistical data for organic acids and very-long-chain fatty acids in blood of mice with mutations in the *Slc5a6* gene fed standard chow or biotin-supplemented chow. For each parameter, raw two-way ANOVA p-values and panel-wide BKY-adjusted q-values are reported for the genotype × supplementation interaction, supplementation, and genotype effects. Pairwise comparisons are reported as within-parameter BKY-adjusted q-values. Panel-wide correction was applied separately to each omnibus effect across all 76 analyzed metabolic parameters. Pairwise q-values are color-coded as follows: green — q < 0.05, orange — q < 0.01, red — q < 0.001.

		q values								
		**placebo**			**biotin**			**WT**	***Slc5a6***$^{R280W/P437Q}$	***Slc5a6***^{P437Q}
**Parameter**		WT vs. *Slc5a6*$^{R280W/P437Q}$	WT vs. *Slc5a6*^{P437Q}	*Slc5a6*$^{R280W/P437Q}$ vs. *Slc5a6*^{P437Q}	WT vs. *Slc5a6*$^{R280W/P437Q}$	WT vs. *Slc5a6*^{P437Q}	*Slc5a6*$^{R280W/P437Q}$ vs. *Slc5a6*^{P437Q}	placebo vs. biotin	placebo vs. biotin	placebo vs. biotin
**OA 3-OMD**	Interaction: P=0.2137, panel q=0.1483, F (2, 22) = 1.657 Supplement factor: P=0.3345, panel q=0.1836, F (1, 22) = 0.9737 Genotype factor: P=0.0060, panel	0.0032	0.221	0.0047	0.447	0.3986	0.2147	0.4244	0.1237	0.4481

	q=0.0007, F (2, 22) = 6.525									
**OA SuAc**	Interaction: P=0.9348, panel q=0.4812, F (2, 22) = 0.06768 Supplement factor: P=0.0002, panel q=0.0002, F (1, 22) = 20.43 Genotype factor: P<0.0001, panel q<0.0001, F (2, 22) = 15.69	0.0104	0.036	0.0005	0.0082	0.0849	0.0012	0.0083	0.021	0.0254
**C20**	Interaction: P<0.0001, panel q<0.0001, F (2, 22) = 16.20 Supplement factor: P<0.0001, panel q<0.0001, F (1, 22) = 62.50 Genotype factor: P<0.0001, panel q<0.0001, F (2, 22) = 16.04	0.9861	0.9861	0.9861	<0.0001	<0.0001	0.0416	0.9415	<0.0001	<0.0001

**C22**	Interaction: P=0.0014, panel q=0.0019, F (2, 22) = 8.977 Supplement factor: P=0.0002, panel q=0.0003, F (1, 22) = 19.95 Genotype factor: P=0.0008, panel q=0.0001, F (2, 22) = 9.975	>0.9999	>0.9999	>0.9999	<0.0001	0.0322	0.0001	>0.9999	<0.0001	0.0983
**C24**	Interaction: P<0.0001, panel q<0.0001, F (2, 22) = 16.16 Supplement factor: P=0.0001, panel q=0.0002, F (1, 22) = 20.93 Genotype factor: P<0.0001, panel q<0.0001, F (2, 22) = 17.95	>0.9999	>0.9999	>0.9999	<0.0001	0.1924	<0.0001	>0.9999	<0.0001	0.636
**C26**	Interaction: P=0.0106,	0.584	0.8451	0.584	0.0021	0.0061	0.1298	0.0472	0.0206	0.4104

	panel q=0.0114, F (2, 22) = 5.630<br>Supplement factor: P=0.3953, panel q=0.2108, F (1, 22) = 0.7517<br>Genotype factor: P=0.1578, panel q=0.0163, F (2, 22) = 2.010									

Supplementary Table 9. Statistical data for ratios derived from acylcarnitine and amino acid blood levels in mice with mutations in the *Slc5a6* gene fed standard chow or biotin-supplemented chow. For each parameter, raw two-way ANOVA p-values and panel-wide BKY-adjusted q-values are reported for the genotype × supplementation interaction, supplementation, and genotype effects. Pairwise comparisons are reported as within-parameter BKY-adjusted q-values. Panel-wide correction was applied separately to each omnibus effect across all 76 analyzed metabolic parameters. Pairwise q-values are color-coded as follows: green — q < 0.05, orange — q < 0.01, red — q < 0.001.

		q values								
		**placebo**			**Biotin**			**WT**	***$Slc5a6^{R280W/P437Q}$***	***$Slc5a6^{P437Q}$***
**Parameter**		WT vs. $Slc5a6^{R280W/P437Q}$	WT vs. $Slc5a6^{P437Q}$	$Slc5a6^{R280W/P437Q}$ vs. $Slc5a6^{P437Q}$	WT vs. *$Slc5a6^{R280W/P437Q}$*	WT vs. *$Slc5a6^{P437Q}$*	*$Slc5a6^{R280W/P437Q}$* vs. *$Slc5a6^{P437Q}$*	placebo vs. biotin	placebo vs. biotin	placebo vs. biotin

**Ratio (C14+C14:1+C16:1)/C0**	Interaction: P<0.0001, panel q<0.0001, F (2, 22) = 62.02<br>Supplement factor: P<0.0001, panel q<0.0001, F (1, 22) = 75.72<br>Genotype factor: P<0.0001, panel q<0.0001, F (2, 22) = 62.69	0.9285	0.9285	0.9285	<0.0001	0.0144	<0.0001	0.2531	<0.0001	0.0548
**Ratio C0/(C16+C18)**	Interaction: P<0.0001, panel q<0.0001, F (2, 22) = 37.13<br>Supplement factor: P<0.0001, panel q=0.0001, F (1, 22) = 24.64<br>Genotype factor: P<0.0001, panel q<0.0001, F (2, 22) = 48.13	0.0536	0.9052	0.0536	<0.0001	<0.0001	0.0823	0.0013	0.0007	<0.0001

**Ratio C14:1/C12:1**	Interaction: P=0.3279, panel q=0.2113, F (2, 22) = 1.173 Supplement factor: P=0.0263, panel q=0.0201, F (1, 22) = 5.673 Genotype factor: P=0.8787, panel q=0.085, F (2, 22) = 0.1301	0.6235	0.5854	0.5518	0.9556	0.8732	0.8732	0.2719	0.7164	0.0146
**Ratio C14:1/C16**	Interaction: P<0.0001, panel q<0.0001, F (2, 22) = 58.36 Supplement factor: P<0.0001, panel q<0.0001, F (1, 22) = 50.60 Genotype factor: P<0.0001, panel q<0.0001, F (2, 22) = 49.42	0.8261	0.8261	0.8261	<0.0001	0.3284	<0.0001	0.5914	<0.0001	0.7343

**Ratio C14:1/C2**	Interaction: P<0.0001, panel q<0.0001, F (2, 22) = 50.73 Supplement factor: P<0.0001, panel q<0.0001, F (1, 22) = 59.91 Genotype factor: P<0.0001, panel q<0.0001, F (2, 22) = 44.28	0.8606	0.8606	0.8606	<0.0001	0.0546	<0.0001	0.5467	<0.0001	0.2788
**Ratio C3/C0**	Interaction: P=0.0639, panel q=0.0551, F (2, 22) = 3.125 Supplement factor: P=0.6724, panel q=0.3228, F (1, 22) = 0.1837 Genotype factor: P=0.0001, panel q<0.0001, F (2, 22) = 14.25	0.0023	0.0023	0.2371	0.1154	0.0003	0.003	0.4955	0.1217	0.0785

**Ratio C3/C2**	Interaction: P=0.0592, panel q=0.0522, F (2, 22) = 3.224 Supplement factor: P=0.2132, panel q=0.1327, F (1, 22) = 1.643 Genotype factor: P<0.0001, panel q<0.0001, F (2, 22) = 29.42	0.0001	0.0059	0.0489	0.0002	<0.0001	0.0755	0.8882	0.5421	0.0097
**Ratio C3/Met**	Interaction: P=0.2042, panel q=0.1469, F (2, 22) = 1.709 Supplement factor: P=0.0351, panel q=0.0262, F (1, 22) = 5.044 Genotype factor: P=0.0383, panel q=0.0041, F (2, 22) = 3.798	0.1387	0.2095	0.5383	0.2167	0.3619	0.0713	0.8076	0.4359	0.0104

**Ratio HADHA**	Interaction: P=0.0021, panel q=0.0027, F (2, 22) = 8.230 Supplement factor: P=0.0106, panel q=0.0094, F (1, 22) = 7.794 Genotype factor: P=0.0002, panel q<0.0001, F (2, 22) = 13.00	0.34	0.0146	0.0146	<0.0001	0.0812	<0.0001	0.1455	0.0007	0.0448
**Ratio MCAD**	Interaction: P=0.6341, panel q=0.3623, F (2, 22) = 0.4651 Supplement factor: P=0.0071, panel q=0.0068, F (1, 22) = 8.810 Genotype factor: P=0.2012, panel q=0.0205, F (2, 22) = 1.726	0.3177	0.3177	0.7297	0.759	0.759	0.759	0.2282	0.0296	0.1679

**Ratio Phe/Tyr**	Interaction: P=0.1420, panel q=0.1082, F (2, 22) = 2.136 Supplement factor: P=0.3374, panel q=0.1836, F (1, 22) = 0.9616 Genotype factor: P<0.0001, panel q<0.0001, F (2, 22) = 49.53	<0.0001	0.0749	<0.0001	<0.0001	0.1491	<0.0001	0.9869	0.0462	0.6213
**Ratio Met/Phe**	Interaction: P=0.2337, panel q=0.1565, F (2, 22) = 1.554 Supplement factor: P=0.6219, panel q=0.3028, F (1, 22) = 0.2502 Genotype factor: P<0.0001, panel q<0.0001, F (2, 22) = 31.47	<0.0001	0.0089	0.018	<0.0001	0.1208	<0.0001	0.3789	0.3361	0.2679

**Ratio (C16+C18:1)/C2**	Interaction: P<0.0001, panel q<0.0001, F (2, 22) = 37.60 Supplement factor: P<0.0001, panel q<0.0001, F (1, 22) = 76.61 Genotype factor: P<0.0001, panel q<0.0001, F (2, 22) = 30.43	0.3019	0.8442	0.3019	<0.0001	<0.0001	0.0071	0.0863	<0.0001	<0.0001

343 **Supplementary Table 10.** Resources

Antibodies		
anti-lipoic acid antibody	Sigma-Aldrich	437695-100UL
Str-HRP conjugate	Imtek	P-S Avs
HRP-conjugated secondary antibodies	Thermo Fisher Scientific	31460
QuickExtract™ DNA Extraction Solution	Lucigen	QE0905T
Taq DNA polymerase	Evrogen	PK113L
HS Taq DNA polymerase	Evrogen	PK017L

MEGAscript™ T7 Transcription Kit	Thermofisher	AM1334
GeneArt™ CRISPR Nuclease mRNA	Thermofisher	A29378
pGEM®-T Easy vector	Promega	A3600
Ion AmpliSeq™ Exome RDY Kit	Thermofisher	A38264
Experimental models: Organisms/strains		
C57Bl/6J	Federal Research Center Institute of Cytology and Genetics, Siberian Branch, Russian Academy of Sciences (ICG SB RAS)	
CBA	ICG SB RAS	
C57Bl/6J FLAD1$^{A418V/A418V}$	Lomonosov MSU	This work
C57Bl/6J FLAD1$^{WT/R542X}$	Lomonosov MSU	This work
C57Bl/6J FLAD1$^{A418V/R542X}$	Lomonosov MSU	This work
F1 hybrids C57Bl/6J × CBA	Lomonosov MSU	Inhouse breeding
Oligonucleotides		
CTTTAGGGAGCCCCTTCCCATC		Mouse *SLC5A6* R280W seq fwd

TCATACTTTCTGGACTTTGGCCTT		Mouse *SLC5A6* R280W seq rev
CACCAGTCCCCAGAAAGTCC		Mouse *SLC5A6* P437Q seq fwd
CAGCAGCCCAACTCTATCACA		Mouse *SLC5A6* P437Q seq fwd3
AGCCTGTGTGTTCCGTTCTT		Mouse *SLC5A6* P437Q seq rev
CTTCTTCCATCGACTCCAGGC		Mouse *SLC5A6* P437Q seq rev3
TGGAGGTGTCTTCATGATGCTCTCCTTGTATGGAGTGAATCAGGCTCAGGTGCAGTGGTACCTCAGTTCCCACTCAGAGAGAGCTGCTGTGCTGTGAGTGCAGGGACTGGAGCAAGGGATGGGAAGGGGCTCCCTAAAGGCAGTGGGGTGGGGACGGGGTTCAGGCTTCTCACCCCCTTACTGGGGACTTTCCGGAGGCT		SLC5A6_R280W2_HR
CTCTATCACATGCATATCTAGGGAGGCAGATAACTCACAAGAGGGTTGGCGCATGGGAAGAACAGTCCAAGGCAGAAGAGTCCCAGCAGTTGCCCTCCAACCATGCCAAAGATACTGAGTGCTGCCTGGAGTCGATGGAAGAAGAACGGAACACACAGGCTCAGAGACTCCAGATTCCAGC		SLC5A6_P437Q_HR
TGTAATACGACTCACTATAGGTATGGAGTGAATCAGGCTCGTTTTAGAGCTAGAAATAGCAAG		SLC5A6_R280W
TGTAATACGACTCACTATAGGAGGCAGAAGAGTCCCAGCAGGTTTTAGAGCTAGAAATAGCAAG		SLC5A6_P437Q

AAAAGCACCGACTCGGTGCC		CRISPR rev
Software and algorithms		
Web tool for selecting target sites for CRISPR/Cas9	chopchop.cbu.uib.no	version 3
Ion Torrent Suite 1	Thermo Scientific	version 5.4
ANNOVAR	https://annovar.openbioinformatics.org	ver. 2018Apr16

5. Supplementary references

Aleshin, V., Borisova, N., Artiukhov, A., Tagirov, K., Solovjeva, O., Lavrenteva, E., et al. (2025). Daytime-Dependent Effects of Thiamine on the Thiamine Pool and Pyruvate Dehydrogenase Regulation in the Brain and Heart. *Int J Mol Sci* 26, 8296. doi: 10.3390/ijms26178296

Angoa-Pérez, M., Kane, M. J., Briggs, D. I., Francescutti, D. M., and Kuhn, D. M. (2013). Marble Burying and Nestlet Shredding as Tests of Repetitive, Compulsive-like Behaviors in Mice. *JoVE*, 50978. doi: 10.3791/50978

Bailey, K. R., and Crawley, J. N. (2009). "Anxiety-Related Behaviors in Mice," in *Methods of Behavior Analysis in Neuroscience*, ed. J. J. Buccafusco (Boca Raton (FL): CRC Press/Taylor & Francis). Available at: http://www.ncbi.nlm.nih.gov/books/NBK5221/ (Accessed June 25, 2024).

Byrne, A. B., Arts, P., Polyak, S. W., Feng, J., Schreiber, A. W., Kassahn, K. S., et al. (2019). Identification and targeted management of a neurodegenerative disorder caused by biallelic mutations in SLC5A6. *NPJ Genom Med* 4, 28. doi: 10.1038/s41525-019-0103-x

Deacon, R. M. J. (2011). Hyponeophagia: A Measure of Anxiety in the Mouse. *JoVE*, 2613. doi: 10.3791/2613

Fahien, L. A., Davis, J. W., and Laboy, J. (1993). Interactions between pyruvate carboxylase and other mitochondrial enzymes. *J Biol Chem* 268, 17935–17942.

Fullerton, M. O., Phillips, L. C., Redgrave, R. E., Spray, L., Haufroid, V., Merces, G., et al. (2026). A Slc5a6-Deficient Mouse Model Reveals Metabolically Driven Cardiomyopathy with Therapeutic Potential for Vitamin-Based Intervention. *JCI Insight*, e200381. doi: 10.1172/jci.insight.200381

Hauth, I., Waterham, H. R., Wanders, R. J. A., van der Crabben, S. N., and van Karnebeek, C. D. M. (2022). A mild case of sodium-dependent multivitamin transporter (SMVT) deficiency illustrating the importance of treatment response in variant classification. *Cold Spring Harb Mol Case Stud* 8, a006185. doi: 10.1101/mcs.a006185

Holling, T., Nampoothiri, S., Tarhan, B., Schneeberger, P. E., Vinayan, K. P., Yesodharan, D., et al. (2022). Novel biallelic variants expand the SLC5A6-related phenotypic spectrum. *Eur J Hum Genet* 30, 439–449. doi: 10.1038/s41431-021-01033-2

Hsieh, C.-H., Lee, J., Sung, H.-H., Huang, Y.-F., Ding, Y.-S., Li, C.-Y., et al. (2023). Novel SLC5A6 mutations lead to B lymphocyte maturation defects with metabolic abnormality rescuable by biotin replenishment. *Clin Immunol* 257, 109855. doi: 10.1016/j.clim.2023.109855

https://benchling.com/ (n.d.).

Kononets, V., Zharmakhanova, G., Syrlybayeva, L., and Nurbaulina, E. (2024). SLC5A6 Gene Mutations Associated with Developmental Delay in a Child: Case Report. *EPHELS* 13, 82–87. doi: 10.55549/ephels.114

Mansour-Hendili, L., Gitiaux, C., Harion, M., Latouche, C., Heron, B., Stojkovic, T., et al. (2024). Recurrent “outsider” intronic variation in the SLC5A6 gene causes severe mixed axonal and demyelinating neuropathy, cyclic vomiting and optic atrophy in 3 families from Maghreb. *Front Genet* 15, 1352006. doi: 10.3389/fgene.2024.1352006

Montomoli, M., Vetro, A., Tubili, F., Donati, M. A., Daniotti, M., Pochiero, F., et al. (2023). A novel SLC5A6 homozygous variant in a family with multivitamin-dependent neurometabolic disorder: Phenotype expansion and long-term follow-up. *Eur J Med Genet* 66, 104808. doi: 10.1016/j.ejmg.2023.104808

Ogawa, E., Kosaki, K., and Takenouchi, T. (2025). Prenatal Brain Abnormalities in Sodium-Dependent Multivitamin Transporter Deficiency. *Am J Med Genet A* 197, e64102. doi: 10.1002/ajmg.a.64102

Perepelkina, O. V., and Poletaeva, I. I. (2022). Selection of Mice for Object Permanence Cognitive Task Solution. *Neurology International* 14, 696–706. doi: 10.3390/neurolint14030058

Pi, B. K., Lee, A. J., Nam, S. H., Chung, K. W., and Choi, B.-O. (2025). SLC5A6 Mutations in Axonal Sensorimotor Polyneuropathy Patients Concurrent With Sodium Dependent Multivitamin Transporter Deficiency and Improved Effects by Multivitamin Therapy. *J Peripher Nerv Syst* 30, e70030. doi: 10.1111/jns.70030

Riley, L. G., Sabui, S., Said, H. M., Niaz, A., Girisha, K. M., Radhakrishnan, P., et al. (2024). Genome sequencing enables diagnosis and treatment of SLC5A6 neuropathy. *Eur J Hum Genet* 32, 947–953. doi: 10.1038/s41431-024-01641-8

Schwantje, M., de Sain-van der Velden, M., Jans, J., van Gassen, K., Dorrepaal, C., Koop, K., et al. (2019). Genetic defect of the sodium-dependent multivitamin transporter: A treatable disease, mimicking biotinidase deficiency. *JIMD Rep* 48, 11–14. doi: 10.1002/jmd2.12040

Subramanian, V. S., Constantinescu, A. R., Benke, P. J., and Said, H. M. (2017). Mutations in SLC5A6 associated with brain, immune, bone, and intestinal dysfunction in a young child. *Hum Genet* 136, 253–261. doi: 10.1007/s00439-016-1751-x

Utsuno, Y., Hamada, K., Hamanaka, K., Miyoshi, K., Tsuchimoto, K., Sunada, S., et al. (2024). Novel missense variants cause intermediate phenotypes in the phenotypic spectrum of SLC5A6-related disorders. *J Hum Genet* 69, 69–77. doi: 10.1038/s10038-023-01206-5

Van Vyve, F.-X., Mercier, N., Papadopoulos, J., Heijmans, C., Dessy, H., Monestier, O., et al. (2024). A new case of sodium-dependent multivitamin transporter defect occurring as a life-threatening condition responsive to early vitamin supplementation and literature review. *Mol Genet Genomic Med* 12, e2388. doi: 10.1002/mgg3.2388

Walimbe, A. S., Waskow, E., Mackay, L., Miller, M., Gijavanekar, C., Difalco, C. R., et al. (2025). Expanded Clinical Phenotype and the Role of Untargeted Metabolomics Analysis in Confirming the Diagnosis of Sodium-Dependent Multivitamin Transporter Deficiency. *Am J Med Genet A* 197, e64014. doi: 10.1002/ajmg.a.64014

Warren, G. B., and Tipton, K. F. (1974). Pig liver pyruvate carboxylase. Purification, properties and cation specificity. *Biochem J* 139, 297–310. doi: 10.1042/bj1390297
